# Positional Isomer of P3HB by Stereoselective Polymerization of Racemic α‑Methyl-β-propiolactone Delivers Polyethylene-like Properties

**DOI:** 10.1021/jacs.6c04108

**Published:** 2026-06-16

**Authors:** Ruirui Li, Jun-Jie Tian, Jiyun Nam, Andrea L. Baer, Eugene Y.-X. Chen

**Affiliations:** Department of Chemistry, 3447 Colorado State University, Fort Collins, Colorado 80523−1872, United States

## Abstract

Biobased and biodegradable poly­(3-hydroxybutyrolactone) (P3HB) has long been considered as a more sustainable alternative to petroleum-based, nonbiodegradable polyolefins, but its properties are far off from its polyolefin (polyethylene, PE, and polypropylene, PP) counterpart’s unique set of thermal (low *T*_g_ below −20 °C and high *T*_m_ > 100 °C) and mechanical (high ductility >350%) characteristics. Here, we report that a positional isomer of P3HB, poly­(3-hydroxy-2-methylpropionate) (P3H2MP), synthesized by catalyst-controlled stereoselective polymerization of racemic α-methyl-β-propiolactone, can match that demanding set of thermomechanical properties of PE. In particular, isotactic-rich P3H2MP, readily synthesized under ambient conditions, reaches high *M*_n_ close to one million Da, *T*_g_ down to −25 °C, *T*_m_ up to 115 °C, tensile strength up to 64 MPa, and toughness up to 131 MJ m^–3^, thus mechanically outperforming PE while maintaining a comparable low *T*_g_ and high *T*_m_ combination. A P3H2MP-based triblock copolymer further widens the low-high temperature window from *T*_g_ = −29 °C to *T*_m_ = 186 °C, resembling those of PP. These results show that P3H2MP exhibits the unique combination of thermomechanical properties matching those of high-performance polyolefins.

## Graphical abstract

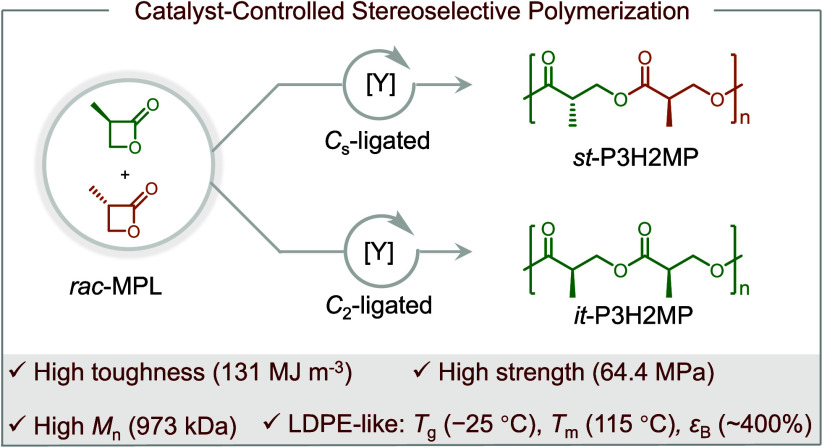

## Introduction

Robust, inexpensive, mass-produced commodity plastics, particularly polyolefins, have played indispensable roles in modern society and the global economy. However, their production relies on limited fossil resources, while their recalcitrance toward degradation has caused severe environmental consequences.^1–4^ Addressing these issues has stimulated increasing interest in searching for renewable and biodegradable alternatives.^5–10^ Among them, poly­(3-hydroxybutyrate) (P3HB), a microbial polyester and the most important member of the large polyhydroxyalkanoate (PHA) family, has emerged as a promising candidate, thanks to its renewable biosynthetic origin and inherent biodegradability.^11–18^ Nevertheless, bacterial (*R*)-P3HB suffers from high brittleness as well as poor thermal stability and melt-processability.^19–22^ Numerous strategies have been explored to address these limitations, including the formation of copolymers with flexible segments,^23–29^ physical blending,^30^ and catalytic stereomicrostructure control.^31,32^ However, these approaches cannot overcome the intrinsic limitations of P3HB, most notably its above-zero glass-transition temperature (*T*_g_ ≈ 0–10 °C). Consequently, P3HB is unsuitable for applications that demand toughness and flexibility under subzero conditions when the P3HB chains freeze into the glassy state, representing a fundamental barrier to its broader utility. In contrast, polyethylene (PE) materials, particularly low-density PE (LDPE), a widely used packaging material, exhibit a much lower *T*_g_, typically below – 20 °C, which enables excellent flexibility (elongation at break, *ε*_B_ > 350%) and toughness even in cold environments, the properties essential for packaging and consumer goods.^7,13,33–35^ On the other hand, LDPE’s low *T*_g_ for its low-temperature stability for application needs is reinforced by its moderately high melting-transition temperature (*T*_m_ > 100 °C), ensuring its robust mechanical performance is maintained before reaching its *T*_m_ and resulting in high-temperature stability as well for a wide temperature application window. This unique set of both low- and high-temperature stability of polyolefins, exemplified here by LDPE, highlights the stark contrast between PE and P3HB and thus the need to overcome both issues with stability at both low- and high-temperatures and with intrinsic brittleness of P3HB (Figure 1A).

**Figure 1:**
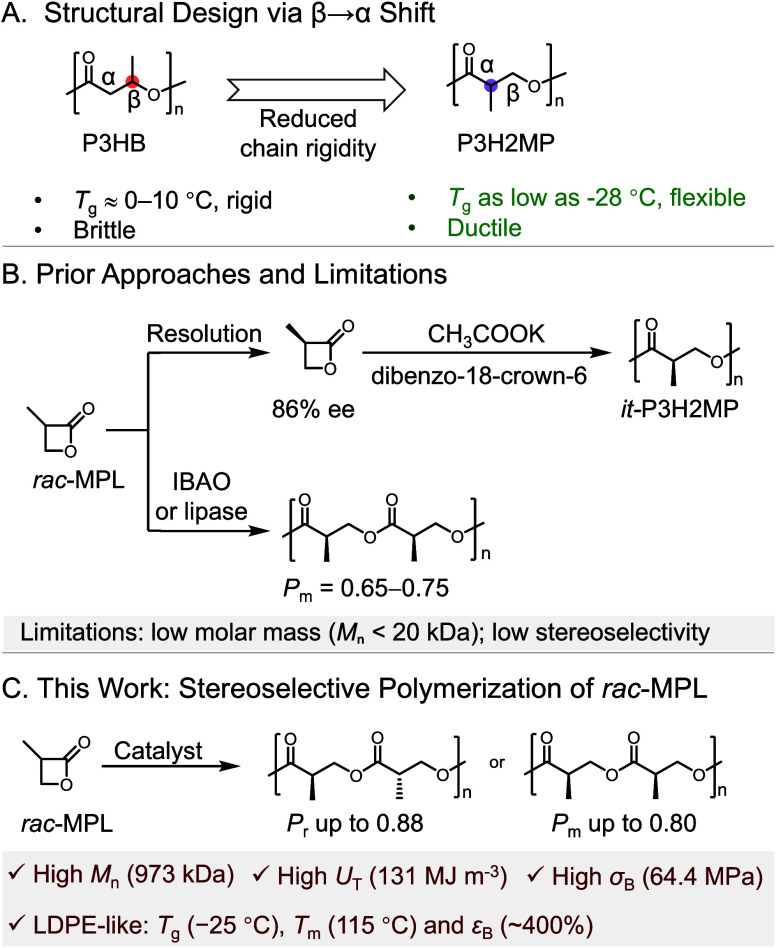
(A) Backbone substitution from β- to α-position improves flexibility and ductility. (B) Known polymerization strategies of *rac*-MPL and limitations.^38–40^ (C) Stereoselective polymerization of *rac*-MPL described in this work.

Current strategies to render PE-like PHA materials involve two common strategies: copolymerization to incorporate both rigid and flexible units, which requires compatible comonomer pairs and controlled compositions for desired thermomechanical properties;^16,26–28^ and modulation of the β-substituents of PHA to tune thermal transition temperatures, which often suffer from *T*_g_ and *T*_m_ trade-offs.^18,25,28,36^ Another approach addressing this challenge while retaining the sustainability and biodegradability aspects of P3HB is to design structurally related PHAs with improved low-temperature performance without compromising high-temperature reinforcement. One such strategy involves shifting the β-methyl group in the P3HB repeat unit to the α position.^37–40^ In this context, poly­(3-hydroxy-2-methylpropionate) (P3H2MP), a positional isomer of P3HB but bearing a methyl substituent at α position, exhibits markedly different thermal properties with *T*_g_ reaching – 20 °C to – 28 °C while keeping a relatively high *T*_m_ of 100–130 °C. This slight substitution’s positional change alters chain packing and mobility, leading to distinctly different crystallization behavior and enhanced mechanical performance (greater flexibility and toughness), rendering P3H2MP a desirable alternative to LDPE.

As P3HB, P3H2MP is also a biodegradable polyester,^41^ and its thermal properties and crystallinity are strongly dependent on tacticity. Except for stereospecific biosynthesis of (*R*)-P3H2MP,^37^ current stereoselective syntheses of P3H2MP have focused on ring-opening polymerization (ROP) of α-methyl-β-propiolactone (MPL): (i) ROP of enantiomerically enriched MPL and (ii) isoselective polymerization of racemic monomer *rac*-MPL (Figure 1B). The former approach requires the preparation of enantioenriched (*R*)-MPL (86% ee) via enzymatic resolution, yielding isotactic (*it*-) P3H2MP (*P*_m_ = 1, *P*_m_ is the probability of *meso* linkages between MPL units).^38^ The latter employed catalysts such as isobutylaluminoxane (IBAO) or lipases to polymerize *rac*-MPL, producing P3H2MP with moderate isotacticity (*P*_m_ = 0.65–0.75).^39,40^ However, both chemical approaches led to P3H2MP with relatively low number-average molar mass (*M*_n_ < 20 kDa), and when *rac*-MPL is used, the stereoselectivity of the ROP is relatively low. Furthermore, the studies to-date have focused on its isotactic forms, leaving the structure and properties of syndiotactic (*st*-) P3H2MP unexplored. As stereomicrostructures play a crucial role in polymer crystallinity, thermal transitions, and mechanical performance, to fully unlock the material potential of P3H2MP, the synthesis of *st*-P3H2MP is of fundamental interest.

Herein, we report the stereoselective synthesis of both *st*- (*P*_r_ up to 0.88) and isotactic-rich (*ir*-) (*P*_m_ up to 0.80) P3H2MP materials via metal-catalyzed stereoselective ROP of *rac*-MPL and subsequent investigation into the influence of tacticity on thermal and mechanical properties of P3H2MP. Through rational selection of the metal center with tailored ligand symmetry as well as steric and electronic environments, polymer tacticity switching is achieved. The resulting P3H2MP exhibits a *T*_g_ down to – 25 °C, a *T*_m_ up to 115 °C, a high molar mass (*M*_n_ up to 973 kDa), as well as high ductility (*ε*_B_ ∼ 400%) and exceptional toughness (up to 130 MJ·m^–3^), demonstrating the thermal and mechanical properties that are suited as a potential replacement for LDPE (Figure 1C). A P3H2MP-based PHA with an even broader application temperature window from *T*_g_ = – 29 °C to *T*_m_ = 186 °C has also been realized through triblock copolymer formation.

## Results and Discussion

### Catalyst-Controlled Stereoselective ROP of *rac*-MPL

As tetradentate alkoxy-amino-bis­(phenolate) yttrium catalysts have shown excellent performance in controlling the syndioselectivity in the ROP of β-butyrolactone (β-BL),^42–44^ such *C*_s_-ligated [Y] complexes (**Y1–Y3**, Figure 2B and Table 1), in combination with initiator [I] ^*i*^PrOH, were initially examined for the ROP of monomer [M] *rac*-MPL at ∼ 23 °C in toluene using a [M]/[Y]/[I] ratio of 200:1:1. Under these conditions, **Y1** with the least sterically hindered ligand produced P3H2MP with a low syndiotacticity, as measured by a low *P*_r_ (probability of *racemic* linkages between MPL units) of 0.53 (Table 1, run 1). A gradual increase in the steric hindrance on the phenolic *ortho*-position of the ligand led to a notable enhancement in stereoselectivity, with *P*_r_ rising to 0.72 (Table 1, runs 2–3). Next, *C*_2_-salen-ligated **Y4** and *C*_s_-salen-ligated **Y5**, which were previously shown to be highly effective in controlling stereochemistry in the ROP of eight-membered diolide monomers, were also examined,^45–47^ but they failed to deliver notable stereoselectivity in the ROP of *rac*-MPL (Table 1, runs 4–5). These results highlight the challenge in controlling stereoselectivity in the ROP of *rac*-MPL, which is far more difficult than the ROP of racemic β-BL or diolides. Given the higher reactivity of *rac*-MPL relative to β-BL with these [Y]-based catalysts, we reasoned that lowering the polymerization temperature may improve syndioselectivity. Indeed, decreasing the polymerization temperature from 23 °C to – 78 °C led to a progressive increase in syndioselectivity (*P*_r_ from 0.72 to 0.86) while maintaining high conversion (>99%) and narrowing dispersity (*Đ* from 1.66 to 1.03) (Table 1, runs 3, 6–8). This trend is consistent with a chain-end control mechanism, in which lower temperatures more effectively discriminate diastereomeric transition states for monomer insertion, thereby amplifying the preference for syndiotactic enchainment. When the polymerization was performed at – 78 °C with a [M]/[Y]/[I] ratio of 1600:0.7:1, **Y3** afforded *st*-P3H2MP with both higher syndioselectivity (*P*_r_ = 0.88) and molar mass (*M*_n_ = 175 kDa, *Đ* = 1.09) (Table 1, run 10). The concurrent narrowing of dispersity at lower temperatures further suggests that chain transfer or other side reactions are suppressed under these conditions. Further optimization of conditions by varying monomer concentration (1 to 5 M) and solvent (from toluene to dichloromethane (DCM)) resulted in no further improvement in syndiotacticity (Table 1, run 9; Table S1, runs 6, 9, 19)

**Figure 2:**
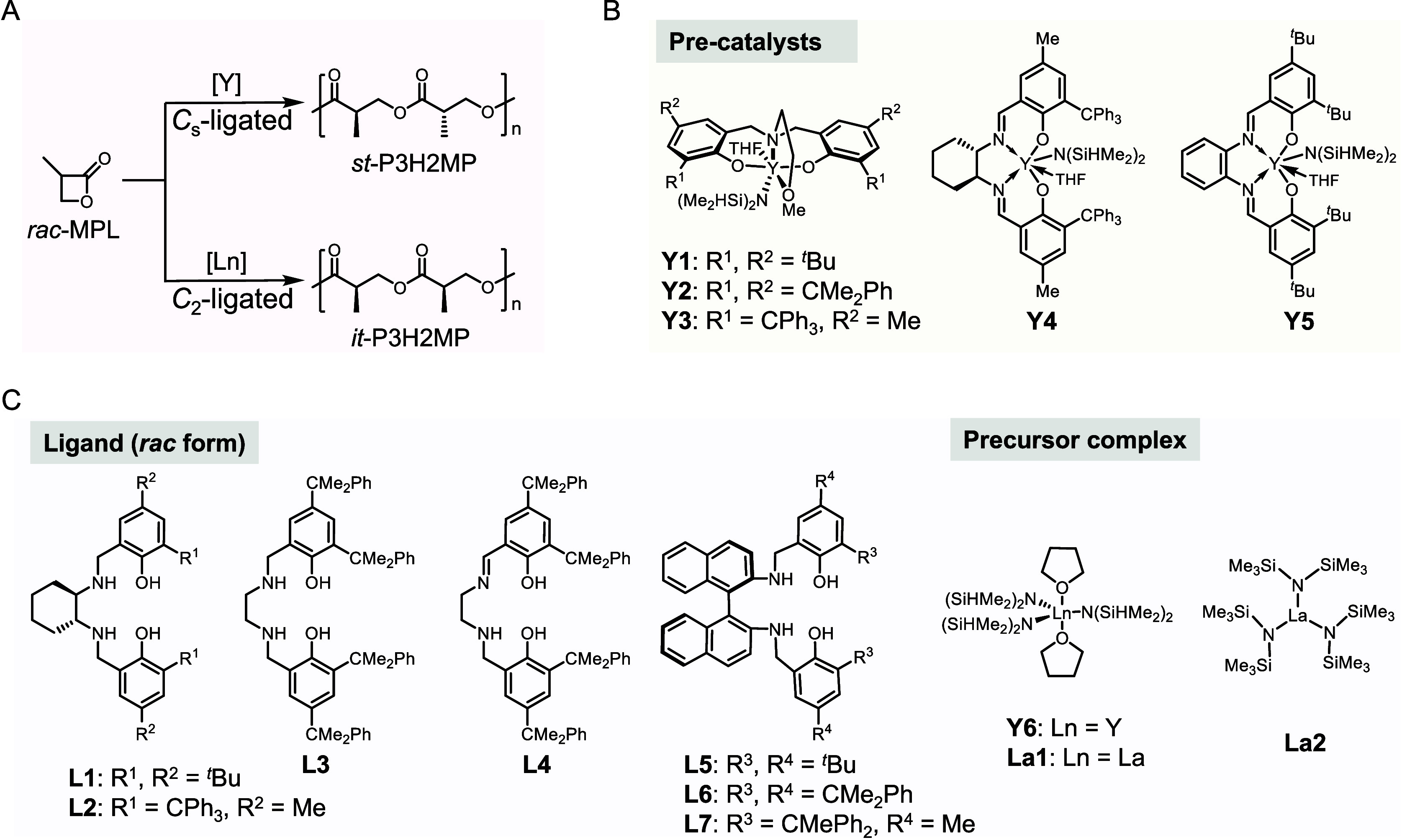
(A) Stereoselective polymerization of *rac*-MPL mediated by *C*_s_- and *C*_2_-symmetric yttrium [Y] and lanthanide [Ln] catalysts. (B) Structures of the yttrium complexes (**Y1**–**Y5**) employed for syndioselective ROP of *rac*-MPL. (C) Structures of the salan or salalen-pro-ligands (racemic form, **L1**–**L7**) and precursor complexes (Ln = Y or La) used for isoselective ROP of *rac*-MPL.

**Table 1:** Selected Results of the Syndioselective ROP of *rac*-MPL^a^

Run	[M]/[Y]/[I]	[Y]	T (°C)	Solvent	*t* (h)	Conv. [%]	*M*_n_ [kDa]^b^	*Đ*	*P*_r_
1	200:1:1	Y1	23	Tol (1 M)	4	>99	22.0	1.16	0.53
2	200:1:1	Y2	23	Tol (1 M)	4	>99	43.9	1.20	0.61
3	200:1:1	Y3	23	Tol (1 M)	4	>99	39.1	1.66	0.72
4	200:1:1	Y4	23	Tol (1 M)	4	>99	29.9	1.19	0.52
5	200:1:1	Y5	23	DCM (2 M)	4	>99	16.8	1.55	0.48
6	200:1:1	Y3	–30	Tol (1 M)	4	>99	43.1	1.34	0.76
7	200:1:1	Y3	–50	Tol (1 M)	4	>99	19.3	1.25	0.80
8	200:1:1	Y3	–78	Tol (1 M)	5	>99	40.9	1.03	0.86
9^c^	1600:0.7:1	Y3	–50	Tol (5 M)	23	>99	189	1.07	0.70
10^c^	1600:0.7:1	Y3	–78	Tol (1 M)	28	51	175	1.09	0.88

a Monomer conversion (Conv.) determined by ^1^H NMR in CDCl_3_. Initiator = ^*i*^PrOH, Tol = toluene, DCM = dichloromethane, *rac*-MPL (1.0 mmol, 86.0 mg).

b Weight-average (*M*_w_) and number-average (*M*_n_) molar mass and dispersity (*Đ* = *M*_w_/*M*_n_) determined via size exclusion chromatography (SEC) at 40 °C in CHCl_3_ coupled with a Wyatt DAWN HELEOS II multi (18)-angle light scattering detector and a Wyatt Optilab TrEX dRI detector for absolute molar mass.

c *rac*-MPL (16.0 mmol, 1.38 g).

We next endeavored to synthesize P3H2MP with a varied degree of isotacticity from possible isoselective polymerization of *rac*-MPL. Rieger and co-workers^32,48^ recently reported that the catalysts prepared *in situ* by combining metal precursor Y­[N­(SiHMe_2_)_2_]_3_(THF)_2_ (**Y6**) with salan or salalen pro-ligands are highly effective in catalyzing stereoselective ROP of *rac*-β-BL. Their high stereoselectivity was attributed to hydrogen-bond donors (NH) and noncovalent interactions (NCIs) introduced by the NH moieties in the ligand. Inspired by this strategy, racemic ligands **L1**–**L4** were prepared to explore their potential for controlling the stereoselectivity of the polymerization of *rac*-MPL at 23 °C. When combined with precursor **Y6**, **L1** yielded essentially atactic (*at-*) P3H2MP (*P*_r_ = 0.51), whereas **L2** afforded syndio-rich (*sr*-) P3H2MP (*P*_r_ = 0.62) (Table 2, runs 1–2). In contrast, salan ligand **L3** produced *ir*-P3H2MP with a moderate isoselectivity (*P*_m_ = 0.66). Notably, salalen pro-ligand **L4** further improved the isoselectivity (*P*_m_ = 0.72; Table 2, run 4), which is presumably attributable to enhanced NCIs between the NH moieties and the propagating chain end, favoring isotactic enchainment.^48^ To further probe the influence of the ligand backbone structure on stereochemical control, an alternative ligand backbone was investigated, which was coupled by systematic tuning of the steric bulk of the substituents on the phenoxy ring directly bonded to the metal center. Specifically, a binaphthyl-derived ligands (**L5**–**L7**) bearing a rigid framework was introduced to replace the more flexible −CH_2_–CH_2_– or cyclohexyl-based backbone linker, with the aim of further improving stereocontrol. Among these, **L6** emerged as the most effective ligand, benefiting from two key structural features: the rigid binaphthyl backbone, which enforces a well-defined chiral environment around the yttrium center; and the bulky CMe_2_Ph substituents on the phenolate rings, which create a sterically demanding coordination sphere that effectively discriminates between the two prochiral faces of the incoming monomer. For example, when comparing the catalysts with the same phenoxy ring substituents (CMe_2_Ph group), the isotacticity by **L6** was noticeably higher, *P*_m_ = 0.80 (Table 2, run 6), relative to that by **L4**, *P*_m_ = 0.72 (Table 2, run 4), demonstrating that the rigid binaphthyl backbone provides superior stereocontrol over flexible ethylene-bridged analogues, consistent with the above analysis. Significantly, **L6** enabled the synthesis of *ir*-P3H2MP with a high molar mass of *M*_n_ = 868 kDa, which was further enhanced to 973 kDa by increasing the [M]-to-[Y] ratio (Table 2, run 9). However, further increasing the steric bulk of the ligand (**L7**) actually lowered isoselectivity (Table 2, run 10), likely due to excessive steric hindrance impeding any effective NCIs between the enchaining and last-inserted monomer units and the catalyst center, indicating that **L6** represents the optimal balance between steric demand and catalytic accessibility in this ligand. In contrast to the above-discussed syndioselective ROP, which responds well to increased syndiotacticity with reduced temperature, here the isoselectivity is primarily governed by the ligand structure. Lowering the polymerization temperature failed to improve isoselectivity but resulted in diminished monomer conversion (Table 2, run 7). To probe the effect of the metal center, lanthanum-based catalysts were also examined. Compared to the yttrium analogue **L6**+**Y6** (*P*_m_ = 0.80, Table 2, run 6), the *in situ* generated catalyst system by combining **L6** with **La1** or **La2** gave P3H2MP with reduced isotacticity (*P*_m_ = 0.74 and 0.59, Table 2, runs 11–12) and lower molar mass (*M*_n_ = 161 and 119 kDa vs 868 kDa), indicating that La^3+^ compromises both stereoselectivity and polymerization control. This comparative result is presumably due to the larger ionic radius and lower Lewis acidity of La^3+^ relative to Y^3+^, which led to a more flexible coordination environment and diminished the ability of the ligand framework to enforce precise monomer insertion geometry.

**Table 2:** Selected Results of the Isoselective ROP of *rac*-MPL^a^

Run	[M]/[Ln]	[Cat.]	T (°C)	Solvent	t (h)	Conv. [%]	*M*_n_ [kDa]^b^	*Đ*	*P*_m_
1	200:1	L1+Y6	23	Tol (1 M)	3	96	100	1.39	0.49
2	200:1	L2+Y6	23	Tol (2 M)	3	>99	87.8	1.07	0.38
3	200:1	L3+Y6	23	Tol (1 M)	3	>99	64.0	1.46	0.66
4	200:1	L4+Y6	23	Tol (1 M)	3	95	44.7	1.75	0.72
5	200:1	L5+Y6	23	Tol (1 M)	18	68	93.4	2.63	0.60
6	200:1	L6+Y6	23	Tol (1 M)	2	>99	868	1.93	0.80
7	200:1	L6+Y6	–30	Tol (2 M)	24	60	601	1.40	0.73
8^c^	500:1	L6+Y6	23	Tol (1 M)	5	>99	851	1.46	0.80
9^d^	500:1	L6+Y6	23	Tol (1 M)	16	91	973	1.42	0.80
10	200:1	L7+Y6	23	Tol (2 M)	17	91	623	1.11	0.56
11	200:1	L6+La1	23	Tol (2 M)	12	>99	119	1.91	0.59
12	200:1	L6+La2	23	Tol (2 M)	4	>99	161	2.90	0.74
13^c^	500:1	L6+La2	23	Tol (2 M)	18	>99	345	2.85	0.75
14^d^	500:1	L6+La2	23	Tol (2 M)	18	>99	287	2.49	0.72

a Monomer conversion (Conv.) determined by ^1^H NMR in CDCl_3_. Tol = toluene, *rac*-MPL (1.0 mmol, 86.0 mg).

b Weight-average (*M*_w_) and number-average (*M*_n_) molar mass and dispersity (*Đ* = *M*_w_/*M*_n_) determined via size exclusion chromatography (SEC) at 40 °C in CHCl_3_ coupled with a Wyatt DAWN HELEOS II multi (18)-angle light scattering detector and a Wyatt Optilab TrEX dRI detector for absolute molar mass.

c *rac*-MPL (1.25 mmol, 107 mg).

d *rac*-MPL (12.5 mmol, 1.07 g).

### P_3_H_2_MP Stereomicrostructures and Stereochemical Control

Figure 3A shows the partially expanded ^1^H NMR spectra of P3H2MP with different tacticities in the region corresponding to the methylene protons adjacent to the ester moiety. The stereosequence sensitivity of these resonances is clearly evident, as the splitting patterns vary significantly with tacticity. ^13^C NMR spectroscopy was employed to determine the stereoregularity of P3H2MP (Figure 3B). In the carbonyl region, two groups of peaks arising from diad sequences were observed. According to previous assignments,^38^ the resonance at chemical shift (δ) = 173.3 ppm was assigned to the *m* diad (*meso* linkage), while the methyl resonances at δ = 13.86 ppm also corresponded to *m* diad. In contrast to the diad-level information from the carbonyl and methyl regions, the methine region (∼ 39 ppm) displayed three well-resolved signals arising from triad sequences. The resonance at δ = 39.18 ppm was assigned to *rr* triads, while the peak at δ = 39.10 ppm was attributed to *mm* triads. The intermediate resonance at δ = 39.14 ppm was assigned to heterotactic (*mr* + *rm*) triads. For the *ir*-P3H2MP (*P*_m_ = 0.80), the spectra exhibit dominant signals corresponding to *m* diads and *mm* triads, indicating a largely *it*-microstructure. The P3H2MP with *P*_m_ = 0.52 shows a more complex pattern with comparable intensities of *r* and *m* diads, reflecting reduced stereoregularity. In contrast, the *st*-P3H2MP (*P*_r_ = 0.88) displays pronounced *r* signals with the *m* peaks nearly disappearing, demonstrating the formation of the *st*-microstructure.

**Figure 3:**
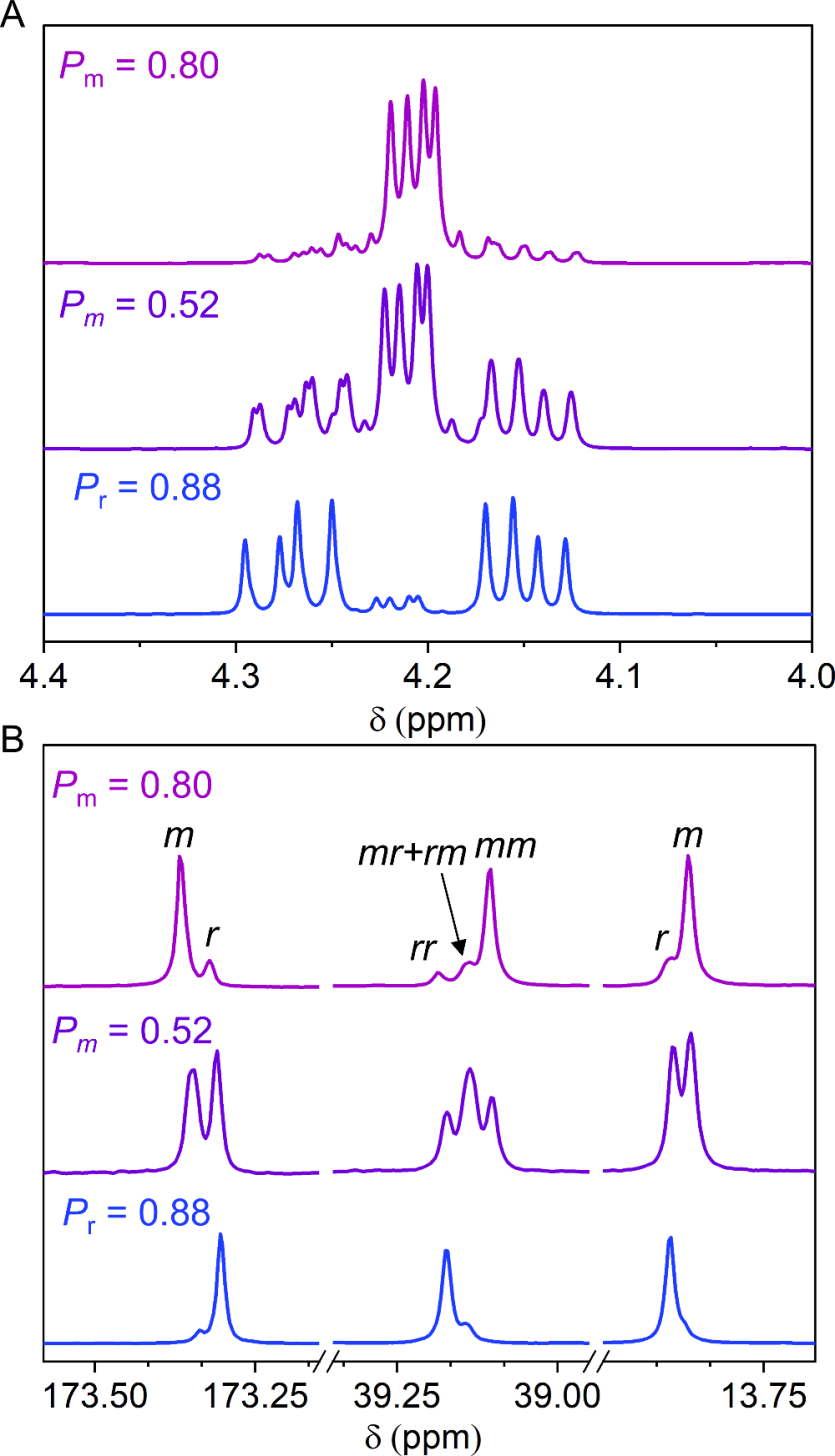
Expanded regions of the ^1^H and ^13^C NMR spectra of P3H2MP with different tacticities. (A) β-Methylene region of the ^1^H NMR spectrum. (B) Carbonyl, methine, and methyl regions of the ^13^C NMR spectrum.

To gain insight into the stereochemical control mechanism for the ROP of P3H2MP, the methine triad distributions were analyzed using a Markovian statistical model (Table S9).^49^ For syndioselective polymerization (*P*_r_ ≥ 0.61), the enantiomorphic model parameter *E*_1_ decreases progressively from 0.81 to 0.24 as *P*_r_ increases from 0.61 to 0.76. Such a systematic decrease of *E*_1_ indicates that monomer insertion becomes increasingly dependent on the configuration of the last inserted unit, which is inconsistent with an enantiomorphic site-control mechanism (for which *E*_1_ ≈ 1 is expected). Meanwhile, the corresponding Bernoulli test parameters remain close to unity {*B* = 4­(*mm*)­(*rr*)/[(*rm*)+(*mr*)][Bibr ref2] = 0.81–1.32}, suggesting that the stereosequence distribution follows first-order Markovian statistics (i.e., the enantio-facial insertion of a new monomer to the growing chain is influenced by the configuration of the last added monomer unit) rather than a Bernoullian process.

In contrast, the isoselective polymerization (*P*_m_ ≥ 0.60) exhibits markedly larger deviations from Bernoullian statistics. The observed *mm* triad fractions increasingly exceed the Bernoullian expectation (*mm* > *P*_m_^2^) at higher *P*_m_ values, indicating systematic enrichment of homotactic sequences. The Bernoulli parameters are significantly larger than unity (*B* = 1.69–5.48), thereby excluding a Bernoullian insertion model and indicating strong correlations between successive monomer insertion events. On the other hand, for *P*_m_ = 0.60 – 0.75 most of the *E*_1_ values were close to unit for predominately an enantiomorphic-site control mechanism. However, for the highest isoselectivity of the series, *P*_m_ = 0.80 produced by **L6** + **Y6**, the pronounced divergence between *E*_1_ (0.61) and *E*_2_ (4.47), Table S9, reveals asymmetric stereochemical reinforcement during propagation, inconsistent with a single, constant stereochemical bias imposed by an enantiomorphic catalyst site. To quantitatively assess this propagation bias, the conditional probabilities *P*_*R*/*SS*_ (the probability of adding an *R* repeating unit to an existing *SS* diad) and *P*_*R*/*SR*_ (the probability of adding an *R* repeating unit to an existing *SR* diad) were evaluated (Figure 4). *P*_*R*/*SS*_ decreases progressively with increasing *P*_m_ and reaches as low as 0.14 at high isotacticity, indicating strong suppression of stereochemical inversion once a homotactic *SS* sequence is established. Notably, *P*_*R*/*SS*_ and *P*_*R*/*SR*_ are statistically inequivalent (Figure 4B), indicating a strong dependence of monomer insertion on stereochemical configuration of the growing chain end. Moreover, the second-order Markovian parameter *Q*_2_ is greater than 1, suggesting a pronounced blockiness in the stereochemical sequence. Furthermore, the *Q*_2_ values closely match 1/(*P*_*R*/*SS*_+*P*_*R*/*SR*_), implying that the polymerization process follows racemic second-order Markovian statistics, in which the insertion of a new monomer is influenced by the configurations of the two preceding units along the growing polymer chain.

**Figure 4:**
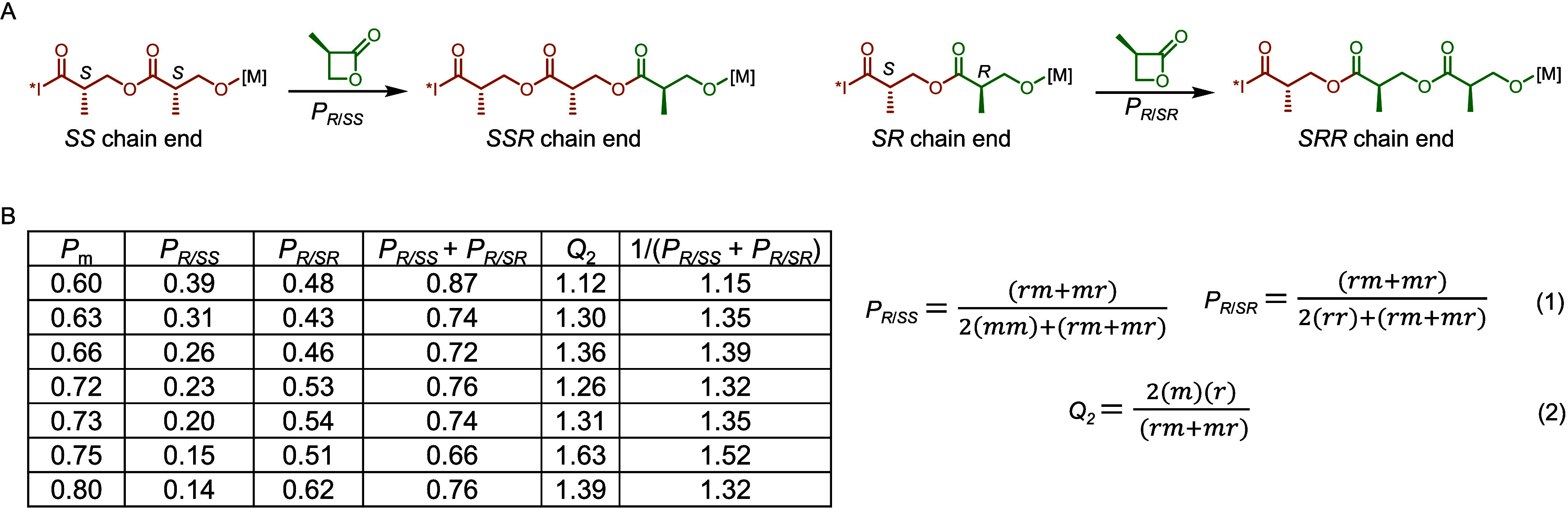
Second-order Markovian parameters for isoselective polymerization of *rac*-MPL.

### Thermal and Mechanical Properties

Thermogravimetric Analysis (TGA) revealed that *st*-P3H2MP (*P*_r_ = 0.88, *M*_n_ = 175 kDa) exhibited a thermal degradation temperature (*T*_d,5%_, corresponding to 5% weight loss) of 261 °C, while the *ir*-P3H2MP (*P*_m_ = 0.80, *M*_n_ = 868 kDa) showed a higher *T*_d,5%_ of 276 °C (Figure 5A). These values noticeably surpass that of bio-P3HB (∼250 °C),^19,45^ indicating that α-methyl substitution in PHA enhanced thermal stability compared to P3HB with β-methyl substitution.^50,51^

**Figure 5:**
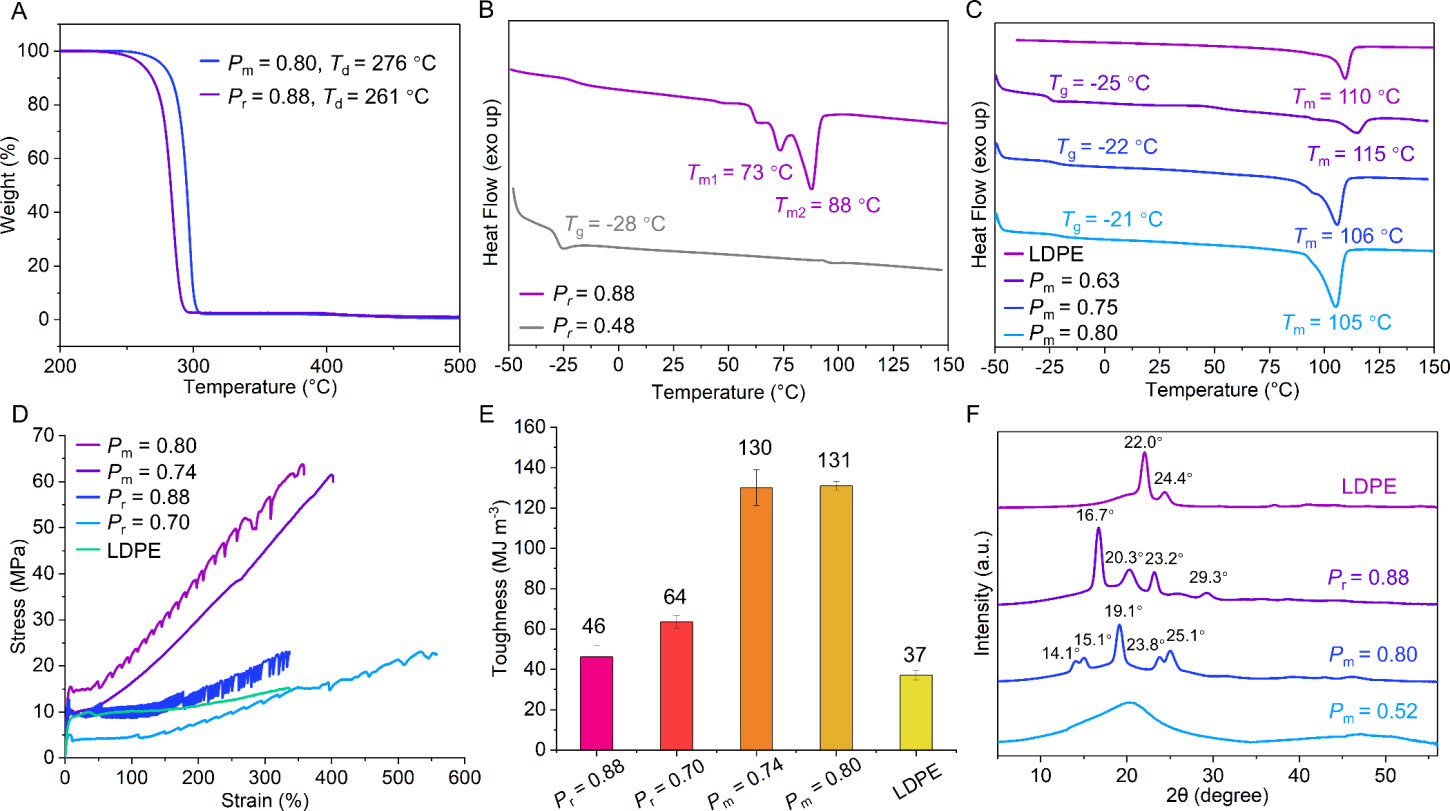
(A) Thermogravimetric analysis (TGA) curves of P3H2MP. (B) First heating DSC curves of *st*-P3H2MP (*P*_r_ = 0.88) and second heating DSC curves of P3H2MP (*P*_r_ = 0.48). Heating rate = 10 °C/min. (C) Second heating DSC curves of P3H2MP with different degrees of isotacticity compared with LDPE. Heating rate = 10 °C/min. (D) Stress–strain curve overlays of P3H2MP with different tacticities compared with LDPE. Strain rate: 5.0 mm/min, ambient temperature. (E) Toughness of P3H2MP with different tacticities compared with LDPE. (F) WAXS profiles of *st*-P3H2MP (*P*_r_ = 0.88), atactic P3H2MP (*P*_m_ = 0.52), *ir*-P3H2MP (*P*_m_ = 0.80), and LDPE.

Differential scanning calorimetry (DSC) analysis showed that P3H2MP with *P*_r_ = 0.48 is amorphous, exhibiting a *T*_g_ of – 28 °C (Figure 5B). For *st*-P3H2MP, the *T*_m_ gradually increased with the higher degree of syndiotacticity, and extrapolation of the fitting of the plot of *T*_m_ *vs P*_r_ for P3H2MP suggested a theoretical *T*_m_ of 109.8 °C at *P*_r_ = 1.0 (Figure S53). Specifically, *st*-P3H2MP (*P*_r_ = 0.88) exhibited two endothermic peaks with *T*_m_ = 73 and 88 °C (heat of fusion (Δ*H*_f_) = 33.5 J/g), which was notably lower than that of *ir*-P3H2MP (*P*_m_ = 0.80, *T*_m_ = 105 °C, Δ*H*_f_ = 40.4 J/g) (Figure 5C). Furthermore, *ir*-P3H2MP displayed faster crystallization, as evidenced by the crystallization exotherm observed during the first DSC cooling scan but no cold crystallization exotherm on the second heating scan (Figure S58). Notably, *ir*-P3H2MP materials exhibit LDPE-like *T*_g_ and *T*_m_ values (Figure 5C). For example, *ir*-P3H2MP (*P*_m_ = 0.63) showed a unique combination of a low *T*_g_ (−25 °C) and a high *T*_m_ (115 °C), which are comparable to those of commercial LDPE (Goodfellow, *M*_w_ = 80.5 kDa) (*T*_m_ = 110 °C, Figure 5C). As expected, the degree of crystallinity continued to improve, as reflected in the rising Δ*H*_f_ values (*P*_m_ = 0.63, Δ*H*_f_ = 14.5 J/g; *P*_m_ = 0.75, Δ*H*_f_ = 34.1 J/g; *P*_m_ = 0.80, Δ*H*_f_ = 40.4 J/g). These results suggest that increased isotacticity enhances the crystalline fraction but does not significantly alter the intrinsic thermal stability of the crystalline phase.

Tensile tests revealed that the stereoregularity of P3H2MP significantly influences its mechanical performance (Figure 5D). Specifically, *st*-P3H2MP (*P*_r_ = 0.88, *M*_n_ = 175 kDa) exhibited a good ductility (*ε*_B_ = 330.7 ± 26.1%) as well as a medium Young’s modulus (*E* = 507 ± 9.3 MPa), yield stress (σ_*y*_ = 13.1 ± 0.3 MPa), and ultimate strength (σ_B_ = 23.1 ± 1.3 MPa). Notably, it displayed a characteristic stress-oscillation (SO) phenomenon which can be influenced by factors such as testing temperature, strain rate, and intrinsic material properties.^52–56^ Rate-dependent tests (Figure S72) showed that the amplitude of SO diminished with increasing strain rate, accompanied by a decrease in yield stress. Likewise, P3H2MP with a lower syndiotacticity (*P*_r_ = 0.70) showed reduced SO, suggesting diminished contribution of crystalline domains to deformation and increased orientation of amorphous chains, thereby suppressing stress fluctuations. Compared with *st*-P3H2MP, *ir*-P3H2MP (*P*_m_ = 0.80, *M*_n_ = 973 kDa) displayed less oscillated tensile curves, improved ductility (*ε*_B_ = 358.6 ± 6.2%) and yield stress (σ_*y*_ = 15.7 ± 0.6 MPa), and superior ultimate strength (σ_B_ = 64.4 ± 1.1 MPa), leading to an exceptional toughness (*U*_T_ = 131 ± 2.1 MJ·m^–3^). In comparison, *st*-P3H2MP (*P*_r_ = 0.88) exhibited the lowest toughness (*U*_T_ = 46 ± 5.5 MJ·m^–3^) (Figure 5E), indicating that isotacticity in P3H2MP promotes both strength and energy absorption, likely due to more effective strain hardening and crystalline reinforcement. Similarly, *ir*-P3H2MP (*P*_m_ = 0.74, *M*_n_ = 419 kDa) displayed smoother tensile profiles, further confirming that crystallinity strongly influences the deformation behavior of P3H2MP during stretching. Importantly, *ir*-P3H2MP consistently outperformed *st*-P3H2MP in tensile strength (Figure 5D and Figure S74), highlighting the ability of isotactic sequences to promote efficient chain packing and robust crystalline reinforcement, thereby increasing load-bearing capacity. For comparison, *ir*-P3H2MP exhibits markedly superior mechanical performance to LDPE (σ_B_ = 13.7 ± 0.7 MPa, *ε*_B_ = 321.8 ± 17.9%, *U*_T_ = 37 MJ·m^–3^),^57^ reaching toughness values up to 131 MJ·m^–3^ (>3.5-fold higher). These results demonstrate that the *ir*-P3H2MP not only rival but significantly outperform commercial LDPE (Figures 6D and 6E), while retaining comparable thermal properties (*T*_m_ = 105–115 °C vs 110 °C for LDPE), establishing it as a suitable alternative to LDPE.

**Figure 6:**
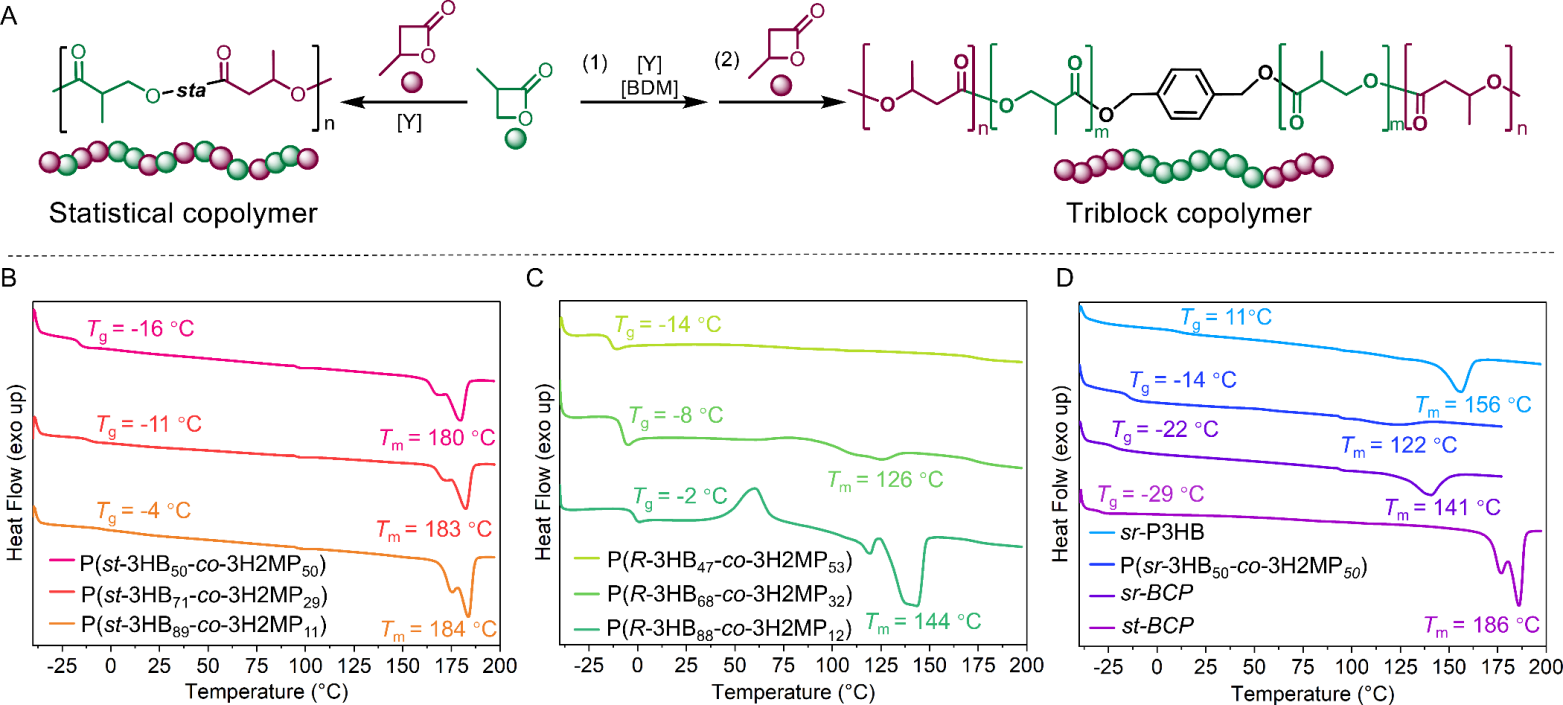
(A) Schematic illustration for the synthesis of statistical copolymers and triblock copolymers. (B) Overlayed DSC curves of syndiotactic statistical copolymers P3HB-*co*-P3H2MP with varying *rac*-MPL incorporation ratios. (C) Overlayed DSC curves of isotactic statistical copolymers P­(*R*-3HB_m_-*co*-3H2MP_n_) with varying *rac*-MPL incorporation ratios. (D) Overlayed DSC curves of homopolymer *sr*-P3HB, statistical copolymer P­(*sr*-3HB_50_-*co*-3H2MP_50_), triblock copolymer *sr*-P3HB_50_-*at*-P3H2MP_100_-*sr*-P3HB_50_ (*sr*-BCP) and triblock copolymer *st*-P3HB_50_-*sr*-P3H2MP_100_-*st*-P3HB_50_ (*st*-BCP).

The wide-angle X-ray scattering (WAXS) profiles were investigated to assess the crystallinity and diffraction characteristics of P3H2MP samples (Figure 5F). As expected, the amorphous P3H2MP (*P*_m_ = 0.52) exhibited only a broad diffuse peak, indicative of a largely disordered morphology. In contrast, the *ir*-P3H2MP (*P*_m_ = 0.80) displayed sharp diffraction peaks at 2θ ≈ 14.1°, 15.1°, 19.1°, 23.8°, and 25.1°, while *st*-P3H2MP (*P*_r_ = 0.88) featured characteristic peaks at 16.7°, 20.3°, 23.2°, and 29.3°. These distinctive diffraction patterns clearly demonstrate that chain tacticity governs the crystal packing motif, as the different stereoregular sequences impose distinct geometric constraints on chain packing in the crystalline lattice. For comparison, commercial LDPE exhibits characteristic diffraction peaks at 22.0° and 24.4°, consistent with its well-known semicrystalline orthorhombic structure. The distinct peak positions of P3H2MP relative to LDPE reflect different crystalline packing geometries arising from the pendant methyl group and tacticity-dependent chain microstructures The calculated degrees of crystallinity were 31% for *ir*-P3H2MP (*P*_m_ = 0.80) and 27% for *st*-P3H2MP­(*P*_r_ = 0.88) (Figures S78 and S81), indicating that isotactic sequences promoted a slightly higher degree of crystalline order, despite the fact that the isotacticity is about 10% lower than the syndiotacticity. Furthermore, the calculated degrees of crystallinity followed a clear tacticity dependence: among syndiotactic samples, crystallinity increased from 18% (*P*_r_ = 0.70) to 27% (*P*_r_ = 0.88), while among isotactic samples it increased progressively from 22% (*P*_m_ = 0.63) to 27% (*P*_m_ = 0.72) and 31% (*P*_m_ = 0.80) (Figure S83). These results collectively establish that higher stereoregularity consistently promotes greater crystalline order, and that the two tacticity types give rise to fundamentally distinct crystal packing motifs, providing a molecular-level rationale for the different thermal and mechanical properties observed between *ir*- and *st*-P3H2MP.

### Statistical and Triblock Copolymers

Syndiotactic and perfectly isotactic (*R*)-P3HB materials exhibit a high *T*_m_ (170–190 °C),^25,28,45^ but their high crystallinity and *T*_g_ (0–10 °C) restrict chain mobility at ambient temperature, resulting in high brittleness. Owing to the low *T*_g_ of P3H2MP, their copolymers with P3HB should provide an effective strategy to balance the *T*_m_ and *T*_g_ values for a broader temperature application window. As shown in Figure 6A, *st*-statistical copolymer P­(*st*-3HB_50_–*co*–3H2MP_50_) with 50% *rac*-MPL incorporation exhibits a low *T*_g_ of – 16 °C together with a high *T*_m_ of 180 °C, indicating the ability for cocrystallization of the syndiotactically placed 3HB and 3H2MP units (isomorphic crystallization). As expected, both the *T*_g_ and *T*_m_ increase systematically with increasing the 3HB content. In contrast, isotactic statistical copolymer P­(*R*-3HB_47_–*co*–3H2MP_53_) is amorphous and displays only a *T*_g_ of – 14 °C, and crystallinity is regained only after the 3HB content become predominate; for example, the copolymer with 88% 3HB content is semicrystalline with *T*_m_ = 144 °C (Figure 6B). These intriguing results highlight pronounced differences in thermal behavior between the two diastereomeric copolymer systems arises primarily from their distinct stereochemical nature and crystallization capability.

Triblock copolymers (tri-BCPs) prepared via sequential monomer addition using 1,4-benzenedimethanol (BDM) as the initiator exhibit thermal characteristics consistent with triblock architectures. Specifically, in sharp contrast to the statistical copolymer of the same comonomer composition, P­(*sr*-3HB_50_–*co*–3H2MP_50_), both the low *T*_g_ (−22 °C) and high *T*_m_ (141 °C) values corresponding to the soft *at*-P3H2MP and hard *sr*-P3HB domain in the tri-BCP *sr*-P3HB_50_–*at*-P3H2MP_100_–*sr*-P3HB_50_ (*sr*-BCP) remain intact, providing strong evidence for the formation of the block copolymer architecture (Figure 6C). This was further supported by SEC analysis, which shows a clear shift to higher molar mass from the *at*-P3H2MP macroinitiator (*M*_n_ = 19.8 kDa, *Đ* = 1.03) to the triblock copolymer (*M*_n_ = 29.6 kDa, *Đ* = 1.19), confirming successful chain extension in this one-pot sequential polymerization (Figure S31). In addition, DOSY NMR analysis revealed a single diffusion coefficient, further supporting this assignment, by excluding the possibility of a physical blend (Figures S23–S26). To further increase the separation between the *T*_g_ and *T*_m_ values and thus broaden the application’s low and high temperature window, we synthesized a *st*-tri-BCP (*st*-BCP), *st*-P3HB_50_–*sr*-P3H2MP_100_–*st*-P3HB_50_. Impressively, this *st*-BCP displays a low *T*_g_ of – 29 °C and a high *T*_m_ of 186 °C. Overall, these results highlight the effectiveness of incorporating low *T*_g_ *at*-P3H2MP segments to tailor the thermal properties of P3HB-P3H2MP copolymer materials.

## Conclusions

In summary, with a goal of developing a PHA performing much like LDPE both thermally and mechanically, we have developed catalyst-controlled stereoselective ROP of *rac*-MPL toward stereoregular P3H2MP materials and investigated their tacticity-dependent materials properties. Depending on catalyst’s ligand symmetry and finely tuned reaction conditions, P3H2MP materials across syndiotactic, atactic, and isotactic-rich stereomicrostructures have been synthesized. Both *st*- and *ir*-P3H2MP PHAs exhibit a desired low *T*_g_ (−28 to – 21 °C), extending their high chain mobility and flexibility to subzero temperatures. On the other hand, *ir*-P3H2MP gives a higher *T*_m_ than its *st*-counterpart (105 – 115 °C vs 88 °C), indicative of more efficient chain packing. Tensile tests also demonstrated tacticity-dependent behavior: While both tactic samples are ductile, *ir*-P3H2MP possesses superior elongation at break (*ε*_B_ = 358.6 ± 6.2%), tensile strength (σ_B_ = 64.4 ± 1.1 MPa), and toughness (*U*_T_ = 131 ± 2.1 MJ·m^–3^), relative to *st*-P3H2MP. Overall, *ir*-P3H2MP, by exhibiting the unique combination of low *T*_g_ and high *T*_m_ resembling LDPE but outperforming LDPE mechanically, has been identified as a suitable PHA alternative to LDPE. In addition, the copolymerization study not only yielded an interesting finding of isomorphic crystallization of syndiotactically (but not isotactically) placed 3HB and 3H2MP units in the statistical copolymer, it also realized a tri-BCP with an even widened *T*_g_ and *T*_m_ window (*T*_g_ = – 29 °C, *T*_m_ = 186 °C), resembling that of PP.

In our prior study on the positional isomer P3HB, we showed that highly isotactic and syndiotactic P3HB materials are both extremely brittle, while *ir*- and *sr*-P3HBs with a moderate degree of stereoregularity are much more ductile and far superior in toughness.^30,31^ Those findings inspired the current study to focus on accessing P3H2MP with similarly moderate tacticities, instead of highly stereoregular forms. A future study will be directed at addressing the question of whether *ir*-P3H2MP is a better material than the P3H2MP with a higher or perfect tacticity. The initial results in this study indicate that the methyl group substituted at the α-position of the β-propiolactone (MPL) is far less effective in rendering stereoselection by the chiral catalyst than the methyl-substitution at the β-position (β-BL). Two potential approaches could address this lower stereoselectivity issue: increasing the steric hindrance of the α-substituents (i.e., modifying the lactone structure) and identifying more stereoselective catalysts (designing more potent catalysts). While the former is more readily achievable (in fact, while this manuscript was in review, Zhu et al. reported the ROP of α-benzyl and *n*-butyl-substituted β-propiolactones into isotactic PHAs),^58^ the resulting PHA may not exhibit thermomechanical properties analogous to LDPE (e.g., a more flexible long-chain alkyl would give a PHA with a desired low *T*_g_ but also with a diminished *T*_m_, or a more rigid group would give a higher *T*_m_, but also a higher *T*_g_, that is, the common *T*_g_ and *T*_m_ trade-offs). The latter approach is preferred in this context but will require more extensive investigation to discover a highly stereoselective catalyst system unique to the ROP of *rac*-MPL, which is currently underway.

## Supplementary Material



## Notes

Experimental details, materials and methods, supporting figures (NMR spectra; SEC traces; TGA and DSC curves; WAXS profiles; tensile plots) and tables (additional polymerization and characterization data). (PDF)
