# Supplementary material for: Positional Isomer of P3HB by Stereoselective Polymerization of Racemic α‑Methyl-β-propiolactone Delivers Polyethylene-like Properties
Source: J Am Chem Soc. 2026 Jun 16;148(25):25877–86. doi: 10.1021/jacs.6c04108 (PMC13339147; doi:10.1021/jacs.6c04108)
Supplement: Supplementary file 1 [file ja6c04108_si_001.pdf]

Supporting Information

Positional Isomer of P3HB by Stereoselective Polymerization of Racemic α-Methyl-β-Propiolactone Delivers Polyethylene-Like Properties

Ruirui Li, Jun-Jie Tian, Jiyun Nam, Andrea L. Baer, and Eugene Y.-X. Chen*

Department of Chemistry, Colorado State University, Fort Collins, CO 80523–1872, United States

Table of Contents

Materials and General Methods **3**

General Polymerization Procedures **3**

Polymer Fractionation **4**

Spectroscopic Characterizations **5**

Thermal Analysis **5**

Mechanical Analysis **6**

Preparation of *rac*-MPL **6**

Preparation of the ligands **7**

Supplementary Figures **12**

Figure S1. ^{1}H and ^{13}C NMR spectra of MPL. 12

Figure S2–S12. ^{1}H and ^{13}C NMR spectra ligands **L1–L11**. 13

Figure S13–S26. ^{1}H and ^{13}C NMR spectra of P3H2MP and copolymers. 24

Figure S27–S37. SEC traces of P3H2MP and copolymers.. 36

Figure S38–S43. TGA and DTG curves of P3H2MP and copolymers 42

Figure S44–S70. DSC curves of P3H2MP and copolymers 45

Figure S71–S76. Tensile curves of P3H2MP........ 58

Figure S77–S83. Wide-angle X-ray scattering (WAXS) profiles… 61

Supplementary Tables **65**

Table S1. Syndioselective ring-opening polymerization of *rac*-MPL. 65

Table S2. Isoselective ring-opening polymerization of *rac*-MPL. 66

Table S3. Ring-opening polymerization of *rac*-MPL with initiator 68

Table S4. Ring-opening polymerization of *rac*-MPL with aluminum complexes 68

Table S5 Synthesis of Triblock Copolymer from *rac*-β-Butyrolactone and *rac*-MPL 69

Table S6 Synthesis of Statistical Copolymer from *rac*-β-Butyrolactone and *rac*-MPL 69

Table S7 Synthesis of Statistical Copolymer of (*R*)-β-Butyrolactone and *rac*-MPL 70

Table S8 Ring-opening polymerization of *rac*-β-butyrolactone 71

Table S9. Diad and triad statistical parameters for evaluating Bernoullian and Markov propagation models. 71

Table S10–S14. Tensile stress-strain data of P3H2MP dog-bone-shaped specimens. 72

References 74

Materials and General Methods

All synthesis and manipulations requiring a dry inert atmosphere were performed under a nitrogen atmosphere using standard Schlenk techniques or in a N_2-supplied glovebox. HPLC-grade organic solvents were first sparged extensively with nitrogen during filling 20 L solvent reservoirs and then dried by passage through activated alumina (for tetrahydrofuran (THF), dichloromethane (DCM)), followed by passage through Q-5 supported copper catalyst (for toluene and hexanes) stainless steel columns. For the toluene and THF used in polymerization reactions, HPLC-grade toluene was degassed and dried over sodium for 12 h, followed by distillation, and stored over activated Davison 4 Å molecular sieves. For the DCM used in polymerization reactions, HPLC-grade DCM was degassed and dried over CaH_2 for 12 h, followed by distillation, and stored over activated Davison 4 Å molecular sieves. Benzyl alcohol (BnOH) and isopropyl alcohol (^{i}PrOH) purified by distillation over CaH_2 and stored over activated Davison 4 Å molecular sieves.

The following reagents were used as received: 3-bromo-2-methylpropionic acid and tetrabutylammonium bromide were purchased from Fisher Scientific Co; $La[N(SiMe_3)_2]_3$ and $Zn[N(SiMe_3)_2]_2$ were purchased from Sigma-Aldrich Chemical Co. Catalyst precursors $Y[N(SiHMe_2)_2]_3(THF)_2$[1] and $La[N(SiHMe_2)_2]_3(THF)_2$,[2] as well as yttrium complexes **Y1-Y3**,[3,4] **Y4-Y5**,[5] and aluminum complexes **Al-1**,[6] **Al-2**[7] used in this study were synthesized according to previous literature procedures. Monomer *rac*-BBL was purchased from Sigma-Aldrich Chemical Co. and purified by distillation over CaH_2 and stored over activated Davison 4 Å molecular sieves before use. Monomer (*R*)-β-butyrolactone was synthesized according to previous literature procedures,[8,9] and purified by distillation over CaH_2 and stored over activated Davison 4 Å molecular sieves before use. Polymer specifications: Thermal and mechanical properties of LDPE (Goodfellow, M_w = 80.5 kDa) were obtained from literature reports.[10]

General Polymerization Procedures

Syndioselective ROP of rac-MPL: In an inert glovebox, a dried flask was charged with the yttrium catalyst and an initiator (benzyl alcohol or isopropanol). The mixture was stirred in dichloromethane or toluene at ambient temperature for 15 min, after which a predetermined amount of monomer was added. The sealed flask was removed from the glovebox and stirred at room temperature. For reactions conducted below room temperature, the premixed catalyst–initiator solution was first removed from the glovebox, cooled to the desired temperature under

nitrogen, and then the monomer was added. After the desired time, the polymerization was quenched by the addition of benzoic acid in $CHCl_3$ (10 mg/mL) and dissolved with $CHCl_3$, followed by precipitation in methanol for 2–3 times. After filtration, the white polymer solid was dried in a vacuum oven at 60 °C to a constant weight.

Isoselective ROP of rac-MPL: In an inert glovebox, a dried flask was charged with the ligand (1.0 equiv.) and yttrium or lanthanum catalyst precursor (1.0 equiv.). The mixture was stirred in toluene at ambient temperature for 3-6 h, after which a predetermined amount of monomer was added. The sealed flask was removed from the glovebox and stirred at room temperature. After the desired time, the polymerization was quenched by the addition of benzoic acid in $CHCl_3$ (10 mg/mL) and dissolved with $CHCl_3$, followed by precipitation in methanol for 2–3 times. After filtration, the white polymer solid was dried in a vacuum oven at 60 °C to a constant weight.

Synthesis of triblock copolymers: In an inert glovebox, a dried flask was charged with the yttrium catalyst and initiator 1,4-benzenedimethanol (BDM). The mixture was stirred in toluene at ambient temperature for 15 min, after which a predetermined amount of monomer MPL was then added. After stirring for 3 h at room temperature, monomer BBL was added sequentially. The flask was sealed, removed from the glovebox, and the reaction mixture was stirred at room temperature for the desired time. The polymerization was quenched by the addition of benzoic acid in $CHCl_3$ (10 mg/mL) and dissolved with $CHCl_3$, followed by precipitation in methanol for 2–3 times. After filtration, the white polymer solid was dried in a vacuum oven at 60 °C to a constant weight.

Synthesis of statistical copolymers: In an inert glovebox, a dried flask was charged with the ligand (1.0 equiv.) and yttrium catalyst precursor (1.0 equiv.). The mixture was stirred in toluene at ambient temperature for 3 h, after which a predetermined amount of monomer *rac*-MPL and *rac*-BBL were added simultaneously. The sealed flask was removed from the glovebox and stirred at room temperature. After the desired time, the polymerization was quenched by the addition of benzoic acid in $CHCl_3$ (10 mg/mL) and dissolved with $CHCl_3$, followed by precipitation in methanol for 2–3 times. After filtration, the white polymer solid was dried in a vacuum oven at 60 °C to a constant weight.

Polymer Fractionation[11]

P3H2MP (M_n = 287 KDa, $Đ$ = 2.49, P_m = 0.72) prepared with catalyst [**L6** + **La2**] was fractionated using acetone as the solvent by Soxhlet extraction for 48 h at 110 °C. The acetone-insoluble

fraction remaining in the thimble was dried in vacuo at 60 °C to a constant weight before the study of thermal and mechanical properties. After fractionation, the polymer exhibited P_m = 0.74.

Spectroscopic Characterizations

Absolute Molar Mass Measurements: Measurements of polymer absolute weight-average molecular weight (M_w), number-average molecular weight (M_n), and molecular weight dispersity ($Đ = M_w/M_n$) were performed via size exclusion chromatography (SEC). The SEC instrument consisted of an Agilent HPLC system equipped with one guard column and two PLgel 5 μm mixed-C gel permeation columns and coupled with a Wyatt DAWN HELEOS II multi (18)-angle light scattering detector and a Wyatt Optilab TrEX dRI detector. The analysis was performed at 40 °C using $CHCl_3$ as the eluent at a flow rate of 1.0 mL min^{-1}, using Wyatt ASTRA v.7.1.2 molar mass characterization software. Wyatt Technology Astra software was used for data analysis.

NMR spectra were recorded on a Varian Inova or Bruker AV-III 400 MHz spectrometer (400 MHz, ^{1}H; 101 MHz, ^{13}C) at 298 K. Chemical shifts (δ) are reported in ppm with the solvent resonance employed as the internal standard (chloroform-d_1 at 7.26 ppm for ^{1}H NMR and 77.2 ppm for ^{13}C NMR Signals are reported as integration, multiplicity (s = singlet, d = doublet, t = triplet, q = quartet, m = multiple, br = broad signal), coupling constant (s) in Hz, and assignment.

WAXS was performed with Xenocs Xeuss 3.0 SAXS/WAXS. The X-ray beam energy was 8,048 keV (Cu Kα, 1.54 Å) with a beam size of 0.7 (horizontal) × 0.7 (vertical) mm2 for the slits closest to the sample. The images were taken with an Eiger2 R 1 M (Dectris) area detector comprising (1,028 × 1,062) pixels with a pixel size of 75 μm2 in transmission geometry. The sample-to-detector distance was 42.5 mm downstream of the sample. Lanthanum hexaboride (LaB6) was used as the standard to calibrate the sample-to-detector distance. The specimen chips are prepared by compression molding using a Carver Bench Top Laboratory Press (Model 4386) equipped with a two-column hydraulic unit (Carver, Model 3912, maximum force 24,000 psi). Degree of crystallinity (χ_c) was calculated as (A_{total}-$A_{amorphous}$)/A_{total}, which indicated the ratio of the area of the crystalline fraction ($A_{crystalline} = A_{total}$-$A_{amorphous}$) to the total area ($A_{total} = A_{amorphous} + A_{crystalline}$) based on peak-area deconvolution of the WAXS profile.

Thermal Analysis

Melting transition (T_m) and glass transition (T_g) temperatures were measured by differential scanning calorimetry (DSC) on an Auto Q20, TA Instrument. All T_m and T_g values were obtained from the second scan unless indicated otherwise. Both heating rate and cooling rate were 10 °C/min unless indicated otherwise. Decomposition temperatures ($T_{d,5\%}$, defined by the temperature of 5% weight loss) and maximum rate decomposition temperatures (T_{max}) of the polymers were measured by thermal gravimetric analysis (TGA) on a Q50 TGA Analyzer, TA Instrument. Polymer samples were heated from ambient temperature to 700 °C at a heating rate of 10 °C/min. Values of T_{max} were obtained from derivative (wt %/°C) *vs.* temperature (°C) plots.

Mechanical Analysis

Tensile stress/strain testing was performed by an Instron 5966 universal testing system (10 kN load cell) on dog-bone-shaped test specimens (ASTM D638 standard; Type V) prepared via compression molding using a Carver Bench Top Laboratory Press (Model 4386) equipped with a two-column hydraulic unit (Carver, Model 3912, maximum force 24000 psi) unless indicated otherwise. Isolated polymer materials were loaded between non-stick Teflon paper sheets into a stainless-steel mold with inset dimensions 30 × 73.5 × 0.38 mm fabricated in-house and compressed between two 6" × 6" steel electrically heated platens, clamp force 3000 psi, at a temperature 10 °C higher than each material's respective T_m. Specimens for analysis were generated via compression molding and cut using an ASTM D638-5-IMP cutting die (Qualitest) to standard dimensions. Mechanical behavior was averaged for all the specimens measured for each individual species investigated. Thickness (0.33-0.37 mm), width (3.18 mm), and grip length (26.4 ± 0.2 mm) of the measured dog-bone specimens were measured for normalization of data by the Bluehill measurement software (Instron). Test specimens were affixed to the screw-tight grip frame. Tensile stress and strain were measured to the point of material break at a grip extension speed of 5.0 mm min^{-1} at ambient conditions.

Synthesis of *rac*-MPL

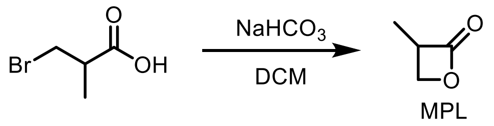

Literature procedures[12] were adopted. 3-Bromo-2-methylpropionic acid (75 g, 449 mmol) was dissolved in distilled water (50 mL) in an ice bath. With the mixture maintained at 0 °C, saturated

$NaHCO_3$ solution was added until the pH reached 7.4–7.8. Dichloromethane (250 mL) and tetrabutylammonium bromide (1.447 g, 1 mol %) were then added, and the biphasic mixture was stirred vigorously at 15 °C for 12 h. The organic phase was separated, washed twice with distilled water (50 mL), dried over anhydrous Na_2SO_4, and concentrated under reduced pressure. To minimize yield loss, the crude product was immediately distilled using a short-path distillation head (room temperature, 200 mTorr) to afford a colorless liquid. For polymerization reactions, the monomer was further purified by fractional distillation from CaH_2 under nitrogen, giving the monomer in 19 % yield. (7.3 g. Note: it's easy to pump off and undergo self-polymerization). ^{1}H NMR (400 MHz, $CDCl_3$) δ 4.38 (dd, J = 6.3, 5.2 Hz, 1H), 3.95 (t, J = 4.8 Hz, 1H), 3.79 – 3.69 (m, 1H), 1.41 (d, J = 7.6 Hz, 3H). ^{13}C NMR (101 MHz, $CDCl_3$) δ 172.5, 66.6, 46.8, 13.0.

Preparation of the ligands

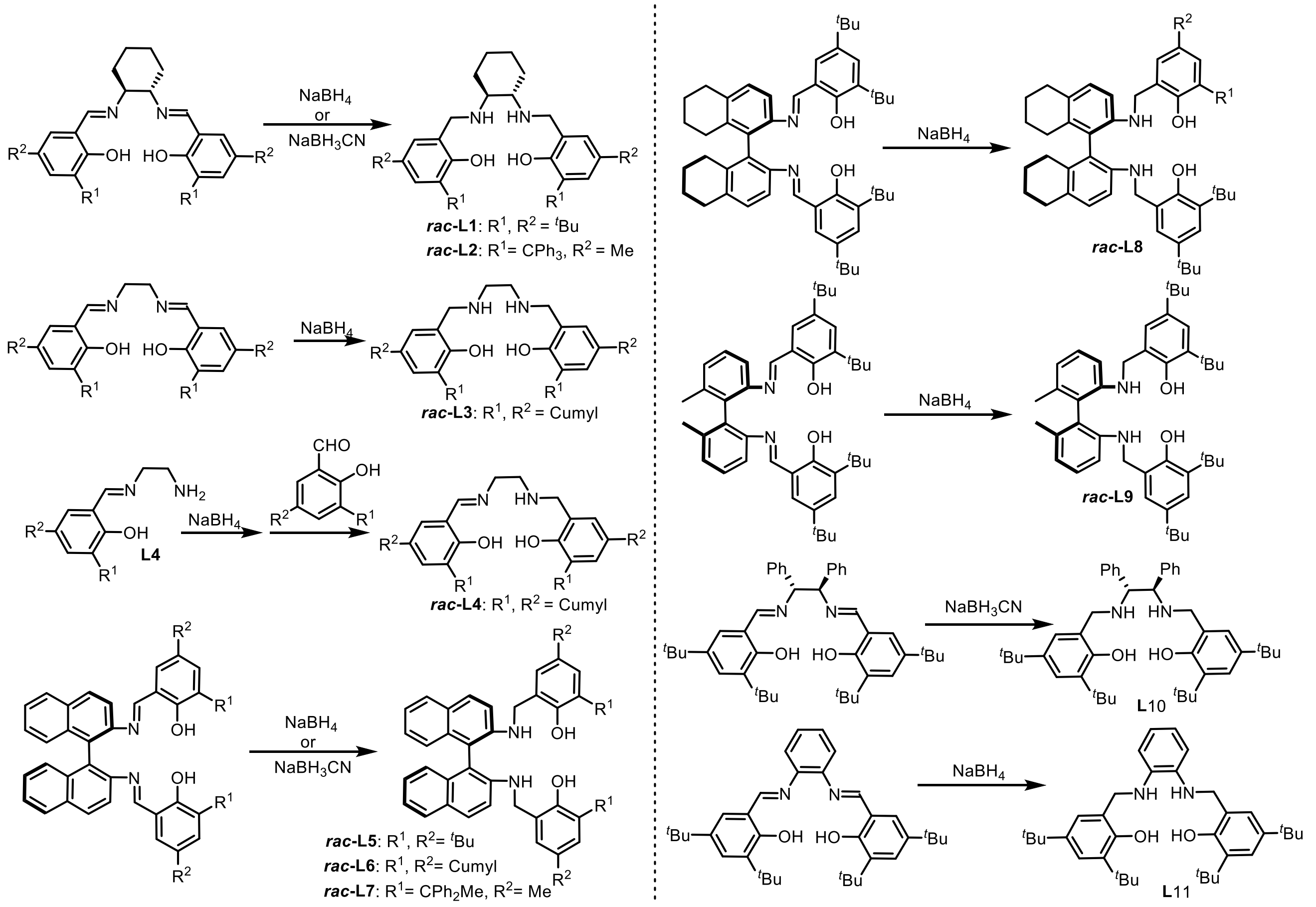

General procedure for the synthesis of salen ligands:

Literature procedures were adopted:[5] A mixture of 3,5-substituted-2-hydroxybenzaldehyde (2.0

equiv.) and corresponding diamine (1.0 equiv.) was dissolved in methanol, and a catalytic amount of formic acid was added to the solution. The reaction was then heated to reflux for 6 h. Upon cooling, the yellow precipitate was collected by filtration and dried under vacuum.

General procedure for preparation of salan and salalen pro-ligands:

Procedure I: literature procedures were adopted:[13] The salen ligand (3.0 mmol, 1.0 equiv.) was dissolved in 10 mL of tetrahydrofuran and 10 mL of methanol. The reaction mixture was cooled to 0 °C and sodium borohydride ($NaBH_4$, 30.0 mmol, 10 equiv.) was added portion-wise. Subsequently, the reaction mixture was allowed to warm to room temperature and stirred for 3 h at this temperature. The solvent was removed under reduced pressure, the residue dissolved in dichloromethane (125 mL), and water (125 mL) was added. The phases were separated, and the organic layer was washed with water (2 × 30 mL) and brine (30 mL). The organic layer was dried over Na_2SO_4, the solvent removed under reduced pressure, and the residue was recrystallized from methanol/dichloromethane.

Procedure II: The salan ligand (2.4 mmol, 1.0 equiv.) was dissolved in a mixture of ethanol/dichloromethane/acetic acid (20/20/1 mL). The solution was cooled to 0 °C, and sodium cyanoborohydride ($NaBH_3CN$, 24.0 mmol, 10 equiv.) was added portion-wise. The reaction mixture was then heated to reflux and stirred until the reaction mixture turned colorless. After cooling to room temperature, the solvent was removed under reduced pressure. The residue dissolved in dichloromethane (125 mL) and water (125 mL) was added. The phases were separated, and the organic layer was washed with water (2 × 30 mL) and brine (30 mL). The organic layer was dried over Na_2SO_4, and the solvent was evaporated under reduced pressure. The crude product was purified by recrystallization from methanol/dichloromethane.

Procedure III: The salalen ligand was prepared following the literature procedure with modification.[14] Dicumylsalicylaldehyde (1.25 g, 3.5 mmol, 1.0 equiv.) was dissolved in 25 mL MeOH:THF (2:1), and ethylendiamine (2.1 g, 35 mmol, 10 equiv.) diluted in MeOH (20 mL) was added dropwise to the reaction mixture and was stirred overnight at room temperature. Subsequently, THF was added (20 mL), and $NaBH_4$ (1.0 g, 26 mmol) was added portion-wise at 0 °C. The reaction mixture was stirred for 2 h, and then allowed to rise to room temperature and subsequently stirred for another 2 h. The solvent was removed in vacuo, and water (50 mL) and DCM (50 mL) were added. The phases were separated, and the organic phase was washed with water (2 × 50 mL), brine (50 mL) and dried over $NaSO_4$. The solution was removed in vacuo, and

the side product was recrystallized in EtOH. The solvent of the supernatant was removed in vacuo, resulting in the crude product L4′ (1.27 g, 3.15 mmol, 90%) as a colorless oil. ^{1}H NMR (400 MHz, $CDCl_3$) δ 7.30-7.26 (m, 4H), 7.25-7.09 (m, 7H), 6.73 (d, *J* = 2.4 Hz, 1H), 3.83 (s, 2H), 2.69 (dd, *J* = 6.4, 5.1 Hz, 2H), 2.55 (dd, *J* = 6.4, 5.1 Hz, 2H), 1.68 (s, 6H), 1.65 (s, 6H).

Dicumylsalicylaldehyde (1.25 g, 3.5 mmol, 1.1 equiv.) and L4' (1.27 g, 3.15 mmol, 1 equiv.) were dissolved in MeOH (30 mL) at room temperature. The reaction mixture was stirred for 24 h, and the resulting suspension was filtered and washed with MeOH (30 mL). The resulting crude product was recrystallized in MeOH:THF, resulting in the salalen pro-ligand *rac*-L4 (1.6 g, 69%) as a slightly yellow solid.

Ligand *rac*-**L1** was prepared according to procedure I. 80% yield, white solid. ^{1}H NMR (400 MHz, $CDCl_3$) δ 10.68 (s, 2H), 7.24 (d, *J* = 2.4 Hz, 2H), 6.89 (d, *J* = 2.4 Hz, 2H), 4.07 (d, *J* = 13.3 Hz, 2H), 3.93 (d, *J* = 13.3 Hz, 2H), 2.53-2.47 (m, 2H), 2.20 (d, *J* = 11.7 Hz, 2H), 1.78-1.71 (m, 2H), 1.41 (s, 18H), 1.32-1.24 (m, 22H). ^{13}C NMR (101 MHz, $CDCl_3$) δ 154.5, 140.8, 136.1, 123.3, 123.2, 122.5, 60.0, 51.0, 35.0, 34.3, 31.8, 30.9, 29.8, 24.3. (It has been characterized in previous literature[11])

Ligand *rac*-**L2** was prepared according to procedure II. 50% yield, white solid. ^{1}H NMR (400 MHz, $CDCl_3$) δ 10.42 (s, 2H), 7.24-7.10 (m, 30H), 6.86 (d, *J* = 2.1 Hz, 2H), 6.69 (s, 2H), 3.81 (d, *J* = 14.0 Hz, 2H), 3.65 (d, *J* = 14.0 Hz, 2H), 2.16 (s, 6H), 1.80 (d, *J* = 12.6 Hz, 2H), 1.52 (d, *J* = 9.1 Hz, 2H), 1.41 (d, *J* = 10.1 Hz, 2H), 0.85 (t, *J* = 10.4 Hz, 2H), 0.50 (d, *J* = 10.9 Hz, 2H). ^{13}C NMR (101 MHz, $CDCl_3$) δ 154.3, 146.3, 133.9, 131.4, 130.5, 128.5, 127.0, 126.4, 125.4, 122.3, 63.2, 57.2, 48.5, 29.6, 24.7, 21.1.

Ligand *rac*-**L3** was prepared according to procedure I. 60% yield, white solid. ^{1}H NMR (400 MHz, $CDCl_3$) δ 10.36 (s, 2H), 7.31-7.26 (m, 8H), 7.22 (d, *J* = 2.4 Hz, 2H), 7.20-7.12 (m, 10H), 7.10-7.04 (m, 2H), 6.68 (d, *J* = 2.4 Hz, 2H), 3.70 (s, 4H), 2.42 (s, 4H), 1.68 (s, 12H), 1.63 (s, 12H). ^{13}C NMR (101 MHz, $CDCl_3$) δ 154.0, 151.7, 151.5, 140.0, 135.4, 128.0, 127.7, 126.9, 125.8, 125.6, 125.5, 124.9, 124.9, 121.9, 53.1, 47.6, 42.6, 42.1, 31.2, 29.6. (It has been characterized in previous literature[13])

Ligand *rac*-**L4** was prepared according to procedure I. 69% yield, white solid. ^{1}H NMR (400 MHz, $CDCl_3$) δ 13.02 (s, 1H), 10.49 (s, 1H), 8.12 (s, 1H), 7.36 (d, *J* = 2.4 Hz, 1H), 7.33-7.06 (m, 22H), 7.00 (d, *J* = 2.2 Hz, 1H), 6.72 (d, *J* = 2.3 Hz, 1H), 3.79 (s, 2H), 3.44 (t, *J* = 5.7 Hz, 2H), 2.75 (t, *J*

= 5.6 Hz, 2H), 1.72 (s, 6H), 1.68 (s, 6H), 1.66 (s, 6H), 1.64 (s, 6H). ^{13}C NMR (101 MHz, $CDCl_3$) δ 167.3, 157.6, 154.2, 151.6, 151.4, 150.8, 150.7, 140.1, 140.0, 136.2, 135.5, 129.3, 128.2, 128.1, 128.0, 128.0, 127.8, 126.9, 126.9, 125.8, 125.7, 125.7, 125.5, 125.5, 125.2, 125.1, 125.0, 122.0, 118.0, 59.1, 52.8, 48.1, 42.6, 42.6, 42.3, 42.2, 31.2, 31.1, 29.7, 29.6. (It has been characterized in previous literature[14])

Ligand *rac*-**L5** was prepared according to procedure I. 66% yield, white solid. ^{1}H NMR (400 MHz, $CDCl_3$) δ 8.33 (s, 2H), 7.98 (d, *J* = 8.9 Hz, 2H), 7.86 (dd, *J* = 8.1, 1.4 Hz, 2H), 7.54 (d, *J* = 9.0 Hz, 2H), 7.37-7.26 (m, 4H), 7.25 (d, *J* = 2.4 Hz, 2H), 7.07 (d, *J* = 8.8 Hz, 2H), 6.95 (d, *J* = 2.4 Hz, 2H), 4.52 (d, *J* = 13.4 Hz, 2H), 4.44 (d, *J* = 13.4 Hz, 2H), 3.86 (s, 2H), 1.36 (s, 18H), 1.28 (s, 18H). ^{13}C NMR (101 MHz, $CDCl_3$) δ 153.6, 143.7, 141.6, 136.6, 133.4, 130.6, 129.7, 128.5, 127.5, 124.2, 124.0, 123.8, 122.1, 116.6, 116.4, 50.3, 35.1, 34.4, 31.8, 29.8.

Ligand *rac*-**L6** was prepared according to procedure I with a modification. The residue was recrystallized from methanol. 73% yield, white solid. ^{1}H NMR (400 MHz, $CDCl_3$) δ 7.78 (d, *J* = 8.8 Hz, 4H), 7.26-7.20 (m, 10H), 7.20-7.05 (m, 18H), 6.87 (dd, *J* = 8.5, 1.1 Hz, 2H), 6.81 (d, *J* = 2.3 Hz, 2H), 6.24 (s, 2H), 4.18-3.99 (m, 4H), 3.77 (s, 2H), 1.63 (s, 12H), 1.53 (d, *J* = 7.6 Hz, 12H). ^{13}C NMR (101 MHz, $CDCl_3$) δ 151.3, 151.2, 149.9, 143.7, 141.4, 135.2, 133.6, 129.7, 128.5, 128.2, 128.1, 128.0, 127.0, 126.8, 126.0, 125.8, 125.7, 125.6, 124.5, 124.4, 124.2, 122.8, 115.3, 114.2, 46.5, 42.7, 42.0, 31.2, 31.1, 30.1, 29.4.

Ligand *rac*-**L7** was prepared according to procedure II with modification. The residue was recrystallized from methanol. 70% yield, white solid. ^{1}H NMR (400 MHz, $CDCl_3$) δ 7.76 (d, *J* = 9.0 Hz, 2H), 7.72 (d, *J* = 7.0 Hz, 2H), 7.19-7.09 (m, 11H), 7.08-6.98 (m, 16H), 6.84 (d, *J* = 8.5 Hz, 2H), 6.68-6.64 (m, 3H), 6.34 (d, *J* = 2.1 Hz, 2H), 4.22-4.12 (m, 2H), 3.99 (d, *J* = 15.0 Hz, 2H), 3.78 (s, 2H), 2.06 (s, 6H), 1.99 (s, 6H). ^{13}C NMR (101 MHz, $CDCl_3$) δ 151.7, 147.7, 147.7, 143.7, 135.3, 133.5, 129.9, 129.6, 128.7, 128.4, 128.4, 128.2, 128.2, 128.1, 127.9, 127.1, 126.3, 126.2, 125.0, 124.2, 123.0, 115.5, 114.5, 51.5, 46.5, 28.7, 21.0.

Ligand *rac*-**L8** was prepared according to procedure I. 77 % yield, white solid. ^{1}H NMR (400 MHz, $CDCl_3$) δ 8.82 (s, 2H), 7.30 (d, *J* = 2.4 Hz, 2H), 7.08 (d, *J* = 8.3 Hz, 2H), 7.04 (d, *J* = 2.4 Hz, 2H), 6.90 (d, *J* = 8.3 Hz, 2H), 4.41-4.35 (m, 4H), 3.50 (s, 2H), 2.84-1.71 (m, 4H), 2.62-2.14 (m, 4H), 1.81-1.69 (m, 8H), 1.44 (s, 18H), 1.34 (s, 18H). ^{13}C NMR (101 MHz, $CDCl_3$) δ 154.0, 141.9, 141.3,

136.4, 136.4, 130.9, 130.1, 124.4, 123.7, 123.7, 122.3, 112.5, 49.7, 35.1, 34.4, 31.8, 29.8, 29.5, 27.5, 23.4, 23.2.

Ligand *rac*-**L9** was prepared according to procedure I. 75 % yield, white solid. ^{1}H NMR (400 MHz, $CDCl_3$) δ 8.46 (s, 2H), 7.30-7.21 (m, 4H), 7.02 (d, *J* = 2.4 Hz, 2H), 6.95-6.89 (m, 4H), 4.38 (q, *J* = 13.4 Hz, 4H), 3.60 (s, 2H), 1.96 (s, 6H), 1.41 (s, 18H), 1.31 (s, 18H). ^{13}C NMR (101 MHz, $CDCl_3$) δ 153.7, 144.7, 141.5, 137.9, 136.4, 129.4, 124.5, 123.8, 123.7, 123.1, 122.2, 112.2, 49.6, 35.1, 34.4, 31.8, 29.8, 19.7.

Ligand **L10** was prepared according to procedure II. 58 % yield, white solid. ^{1}H NMR (400 MHz, $CDCl_3$) δ 7.26-7.22 (m, 6H), 7.21 (d, *J* = 2.4 Hz, 2H), 6.95 (dd, *J* = 6.6, 2.9 Hz, 4H), 6.63 (d, *J* = 2.4 Hz, 2H), 4.05 (s, 2H), 3.79 (d, *J* = 13.3 Hz, 2H), 3.62 (d, *J* = 13.2 Hz, 2H), 1.43 (s, 18H), 1.22 (s, 18H). ^{13}C NMR (101 MHz, $CDCl_3$) δ 154.4, 140.8, 137.6, 136.2, 128.6, 128.4, 128.1, 123.6, 123.3, 121.8, 66.7, 51.3, 35.1, 34.3, 31.8, 29.8 (It has been characterized in previous literature[15])

Ligand **L11** was prepared according to procedure I. 75 % yield, white solid. ^{1}H NMR (400 MHz, $CDCl_3$) δ 8.03 (s, 2H), 7.32 (d, *J* = 2.4 Hz, 2H), 7.09 (d, *J* = 2.4 Hz, 2H), 7.06-7.00 (m, 4H), 4.41 (s, 4H), 3.64 (s, 2H), 1.43 (s, 18H), 1.34 (s, 18H). ^{13}C NMR (101 MHz, $CDCl_3$) δ 153.3, 142.0, 136.8, 136.4, 124.2, 124.0, 122.2, 121.9, 114.6, 49.0, 35.1, 34.4, 31.8, 29.9. (It has been characterized in previous literature[16])

Supplementary Figures

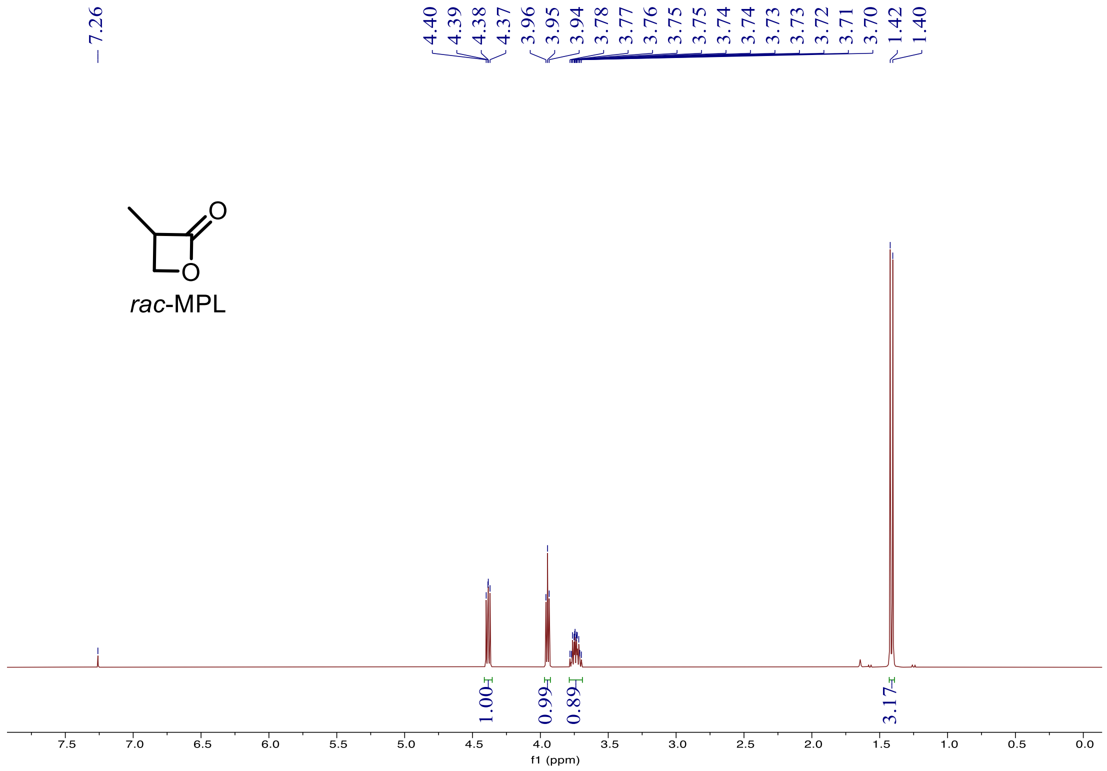

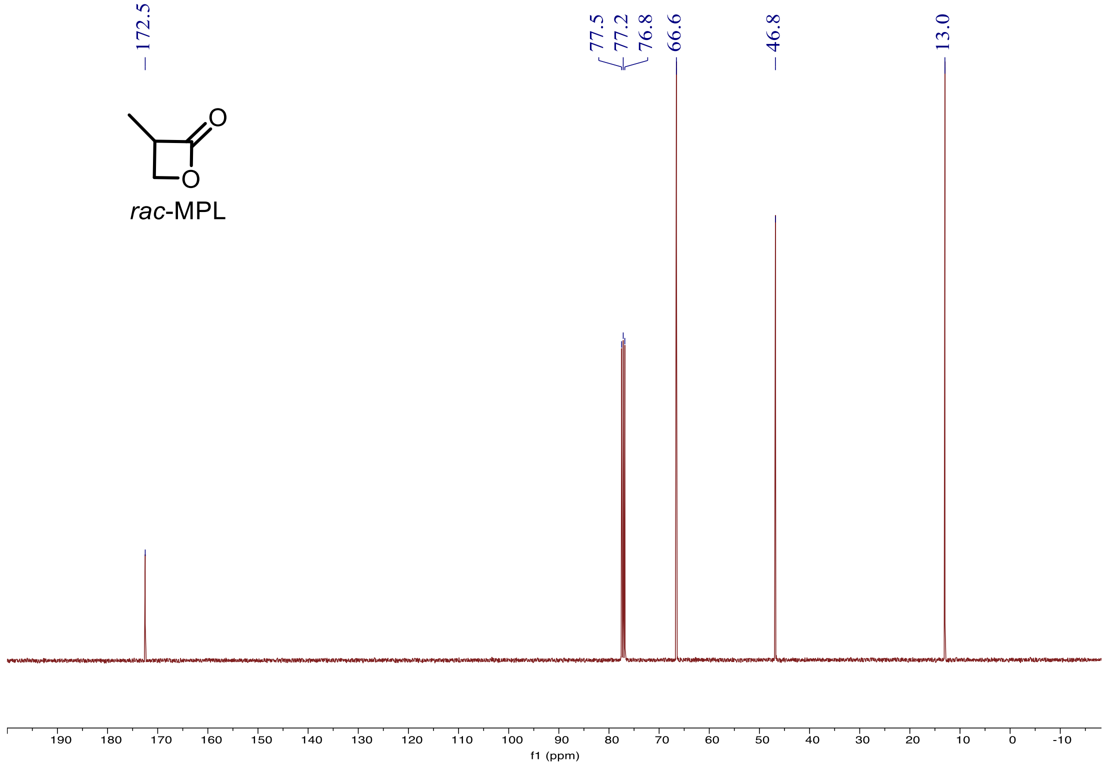

Figure S1. ^{1}H NMR and ^{13}C NMR ($CDCl_3$, 25 °C) spectra of *rac*-MPL.

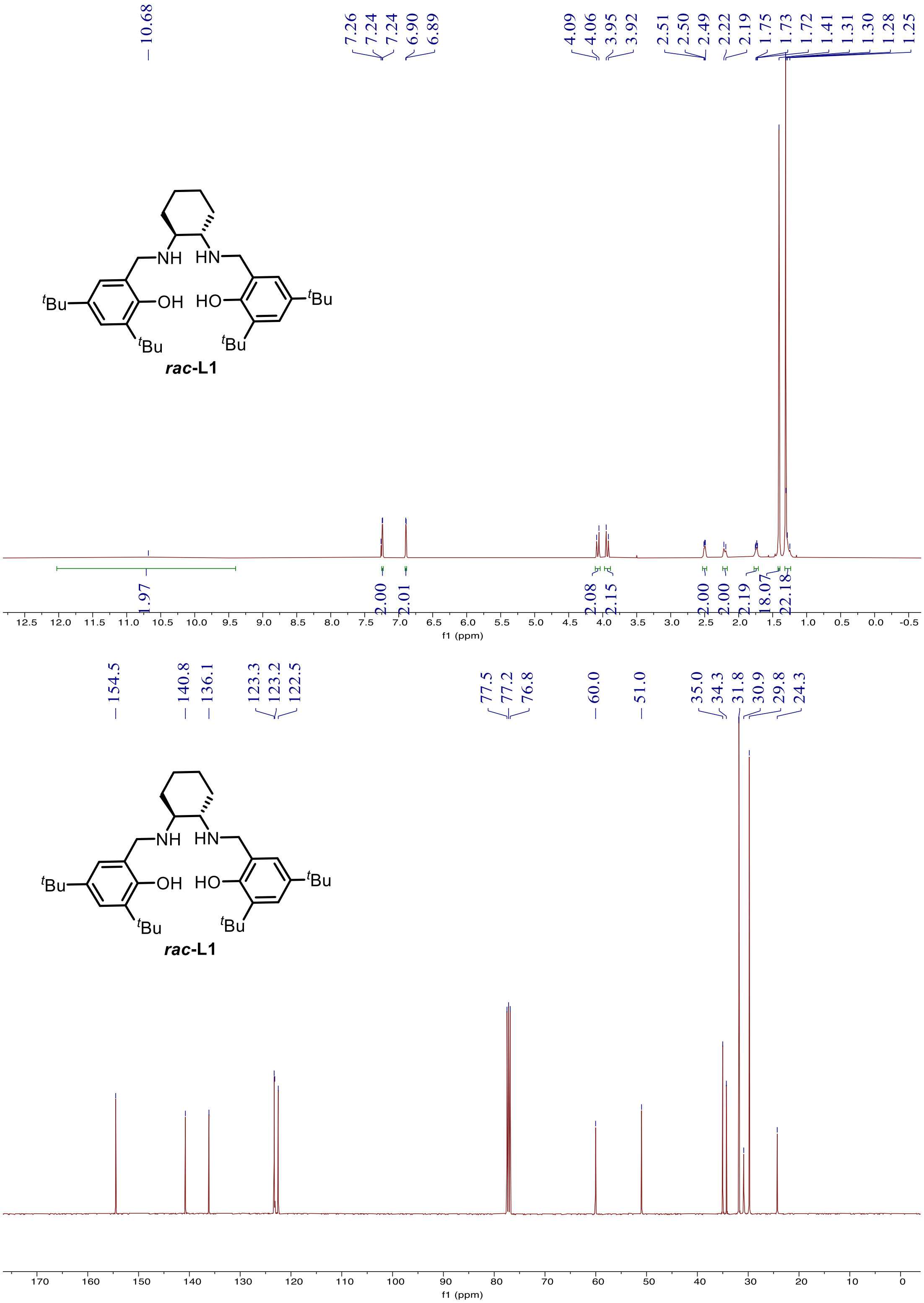

Figure S2. ^{1}H NMR and ^{13}C NMR ($CDCl_3$, 25 °C) spectra of ligand *rac*-**L1**.

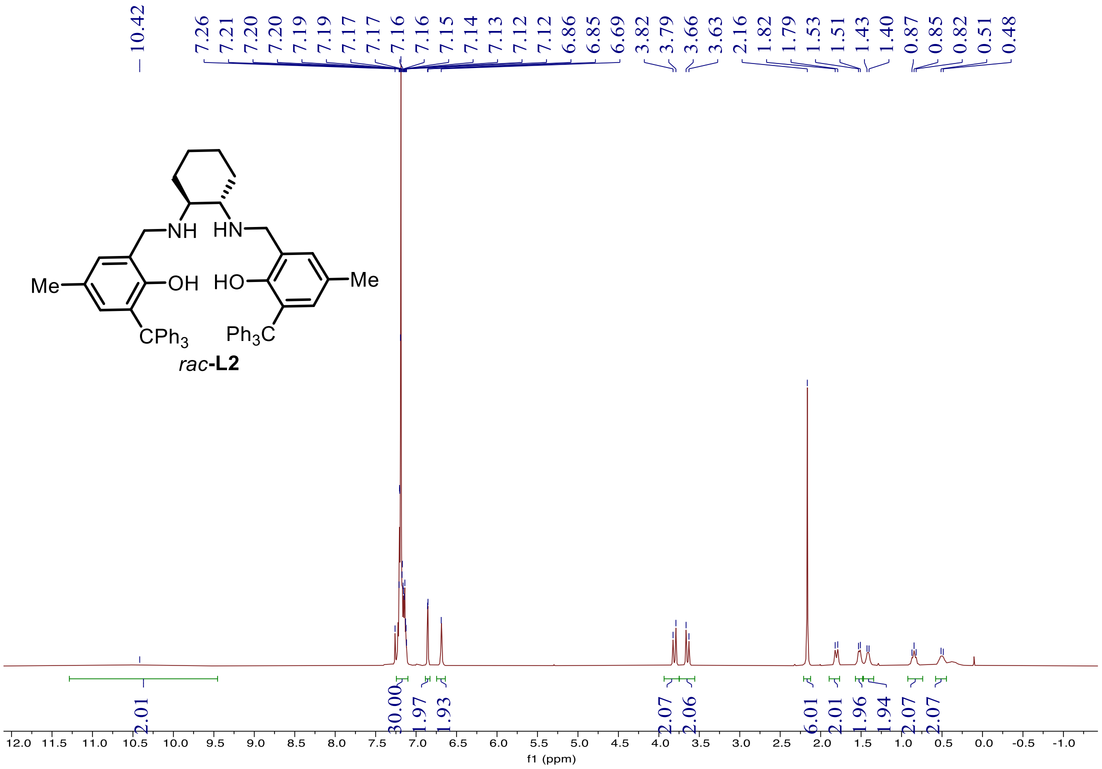

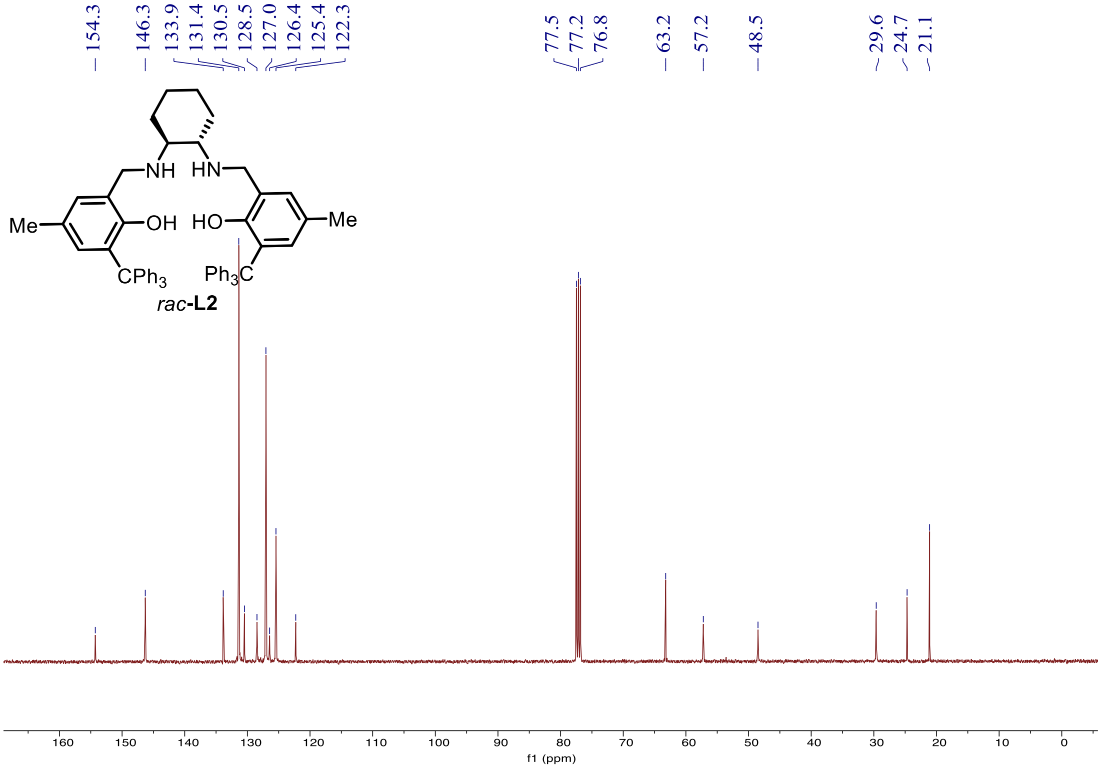

Figure S3. ^{1}H NMR and ^{13}C NMR ($CDCl_3$, 25 °C) spectra of ligand *rac*-**L2**.

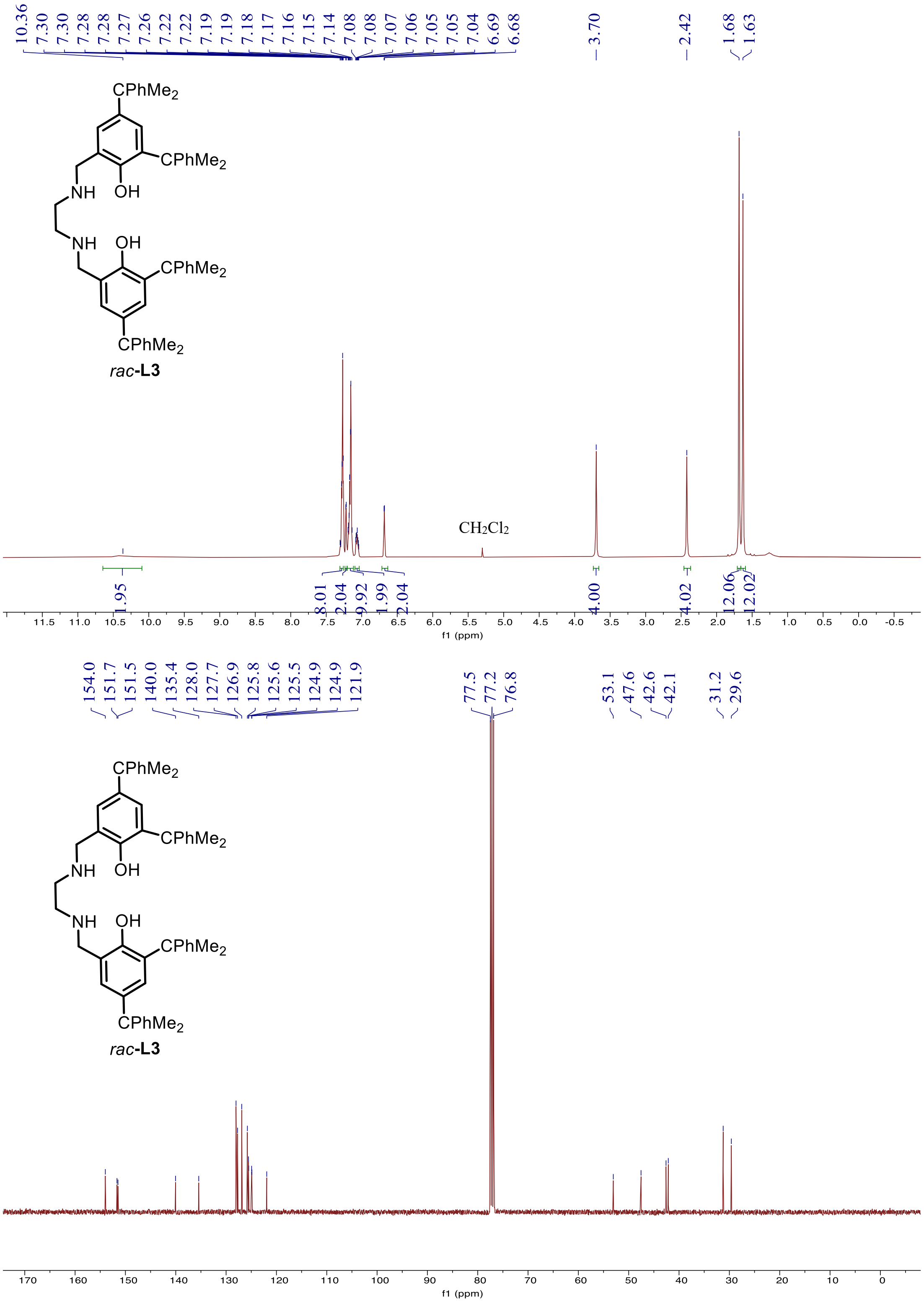

Figure S4. ^{1}H NMR and ^{13}C NMR ($CDCl_3$, 25 °C) spectra of ligand *rac*-**L3**.

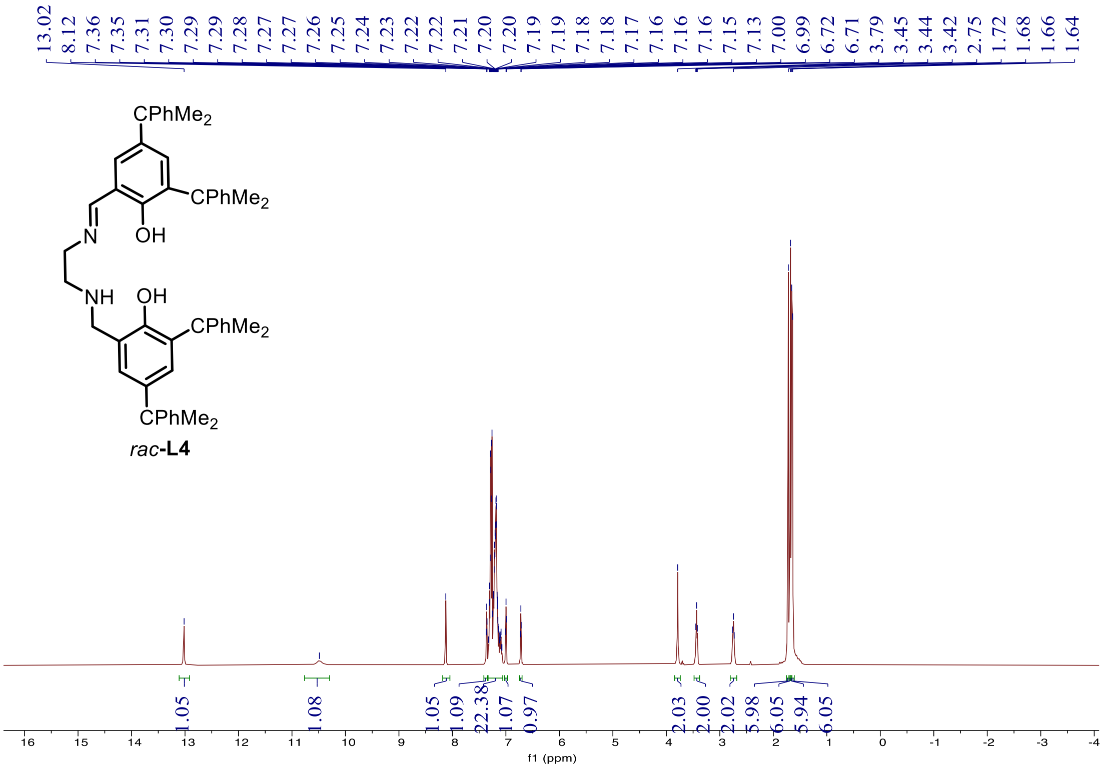

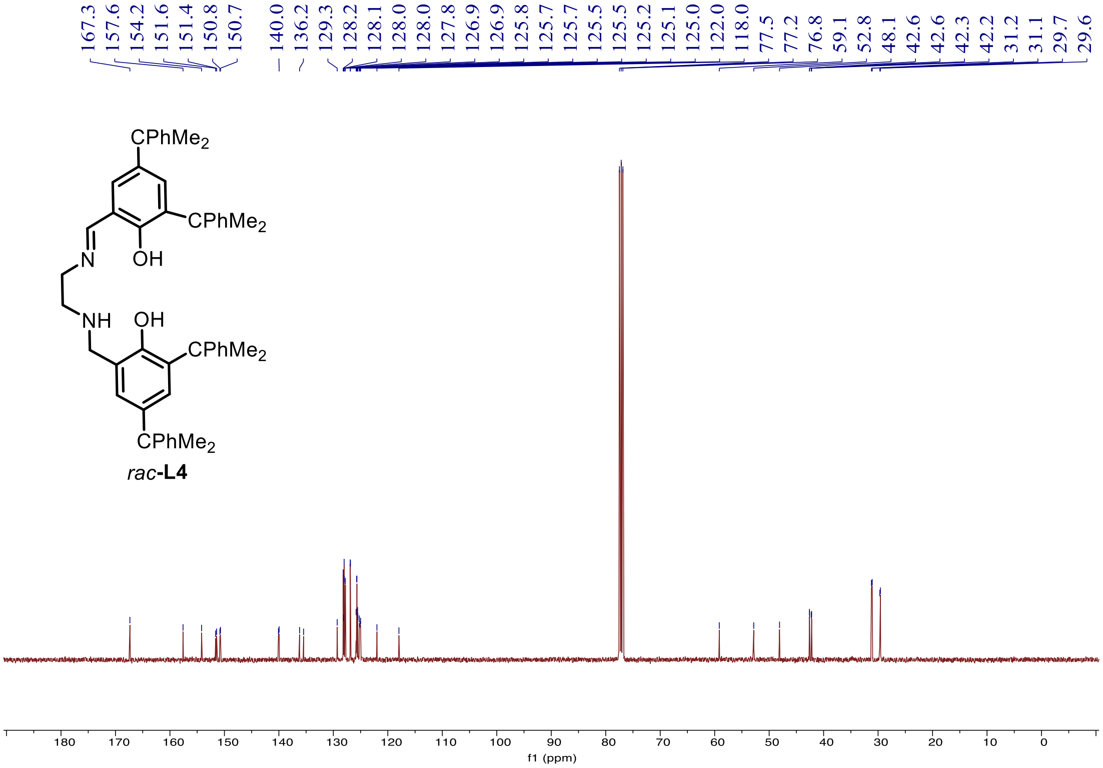

Figure S5. ^{1}H NMR and ^{13}C NMR ($CDCl_3$, 25 °C) spectra of ligand *rac*-**L4**.

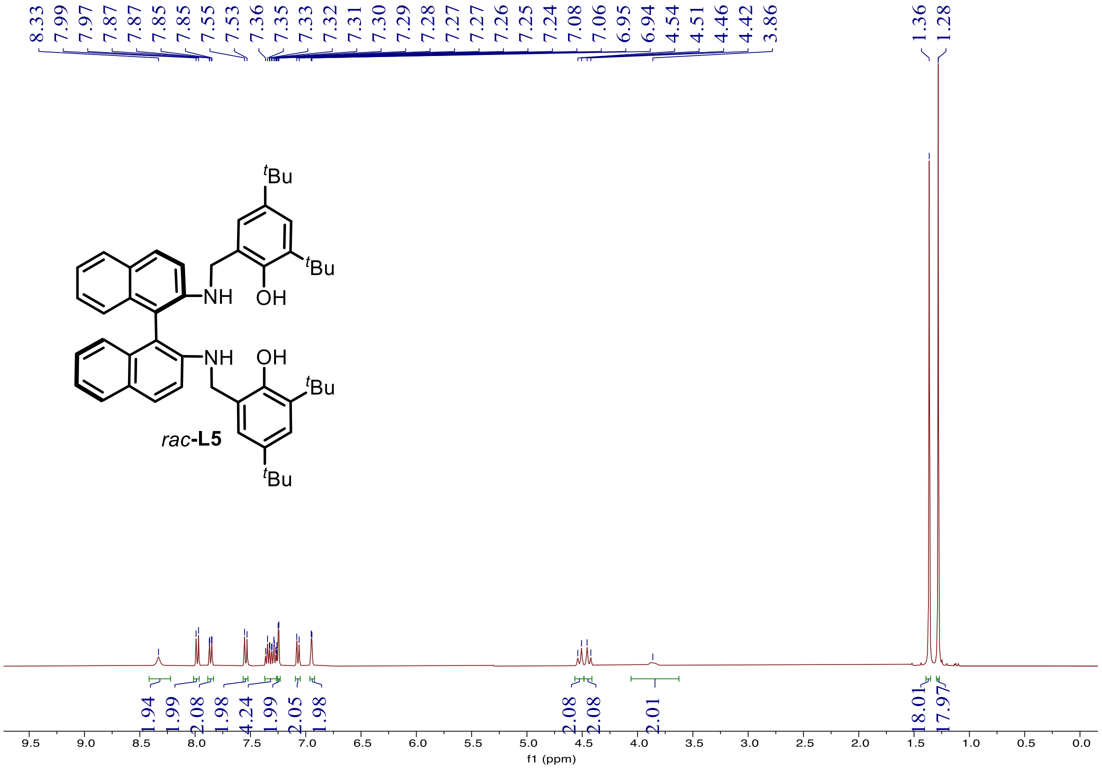

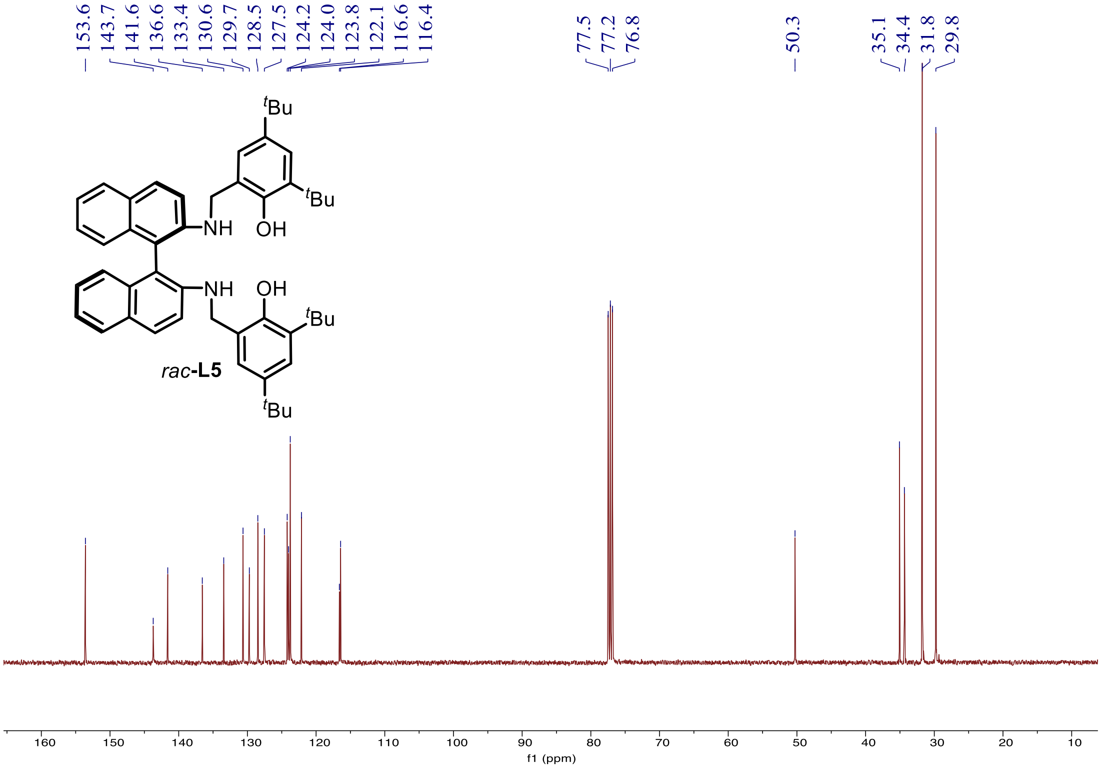

Figure S6. ^{1}H NMR and ^{13}C NMR ($CDCl_3$, 25 °C) spectra of ligand *rac*-**L5**.

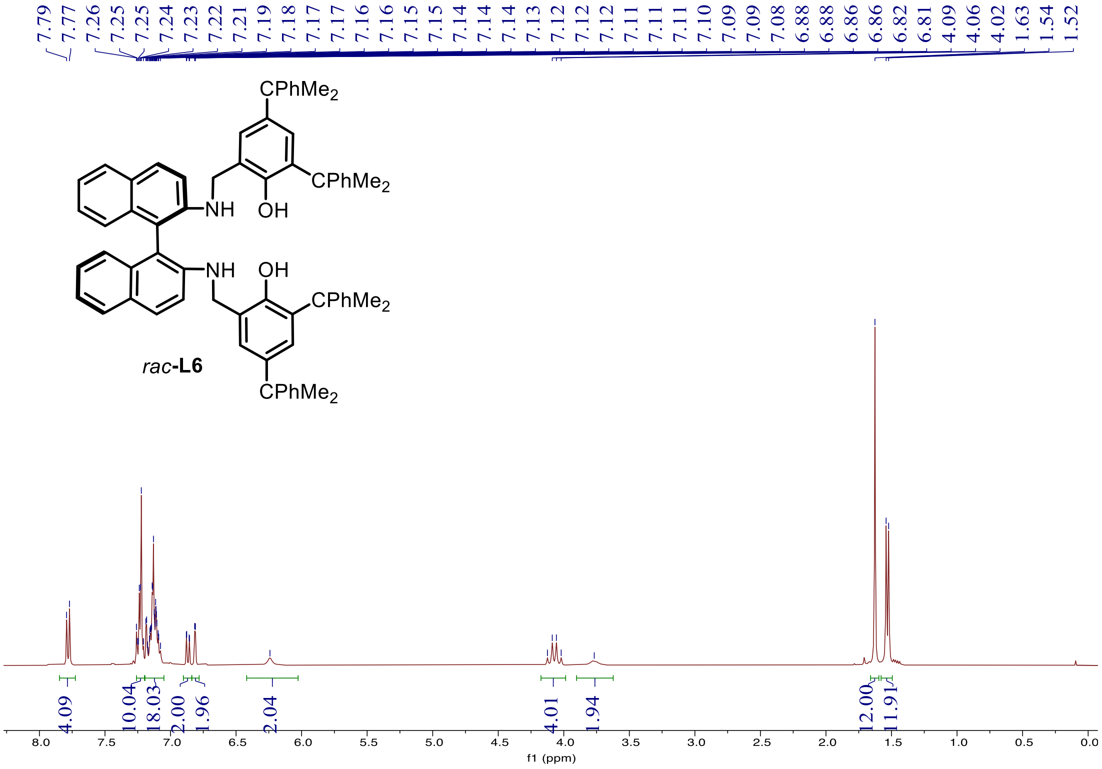

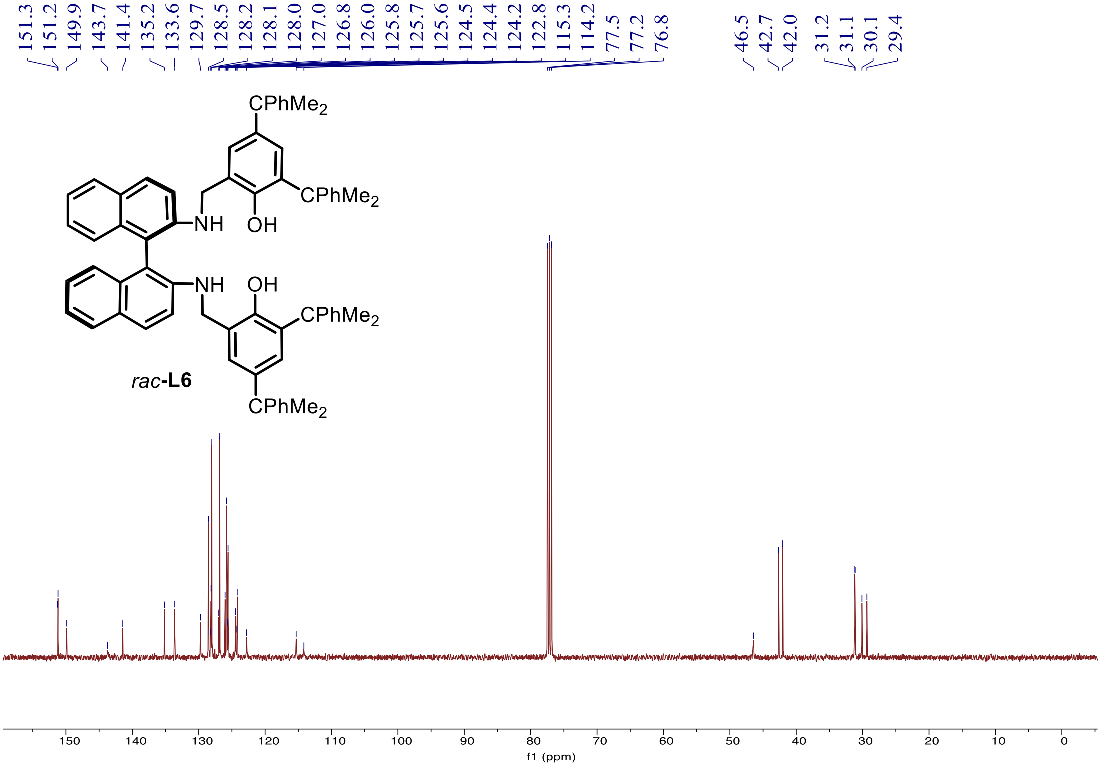

Figure S7. ^{1}H NMR and ^{13}C NMR ($CDCl_3$, 25 °C) spectra of ligand *rac*-**L6**.

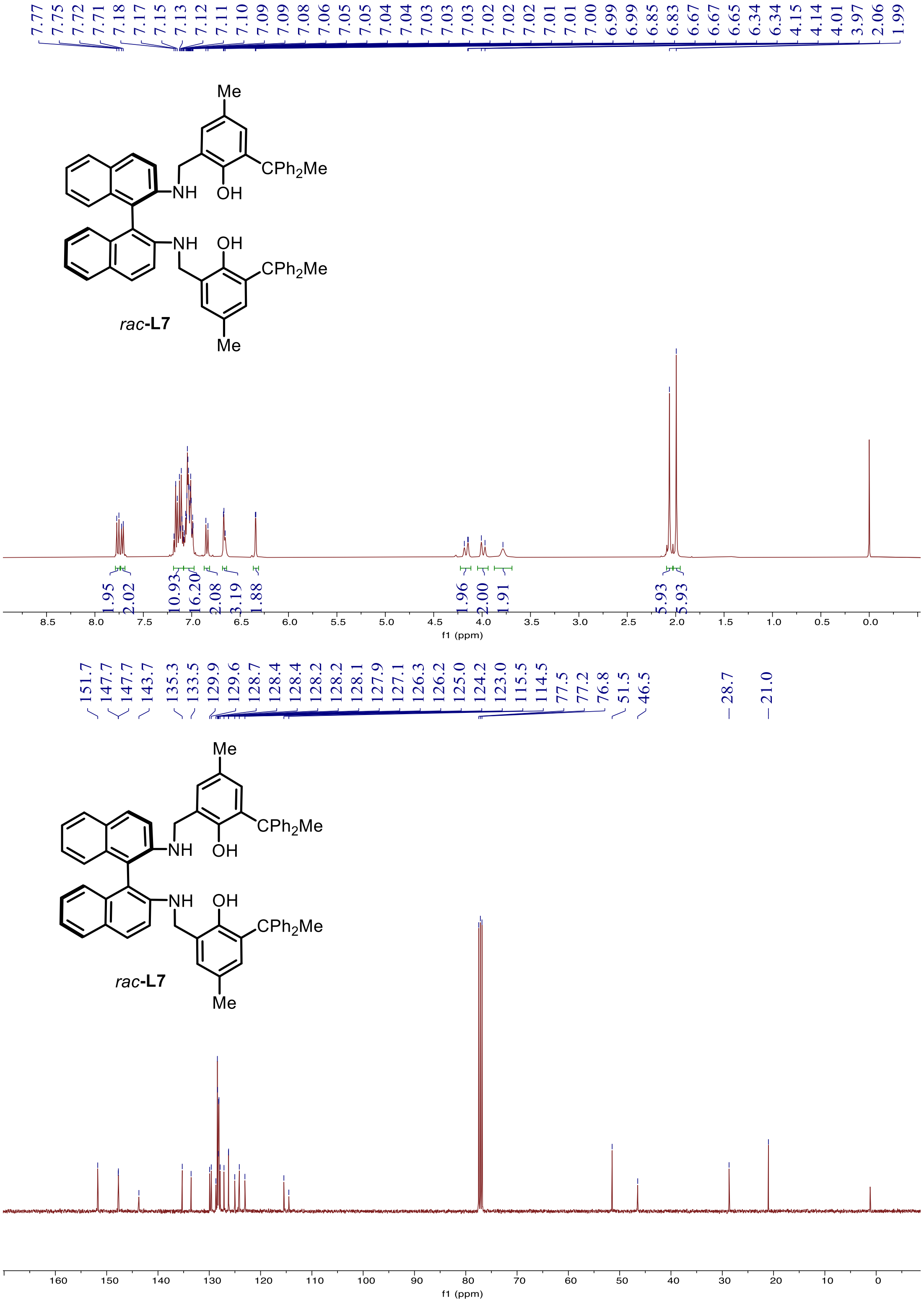

Figure S8. ^{1}H NMR and ^{13}C NMR ($CDCl_3$, 25 °C) spectra of ligand *rac*-**L7**.

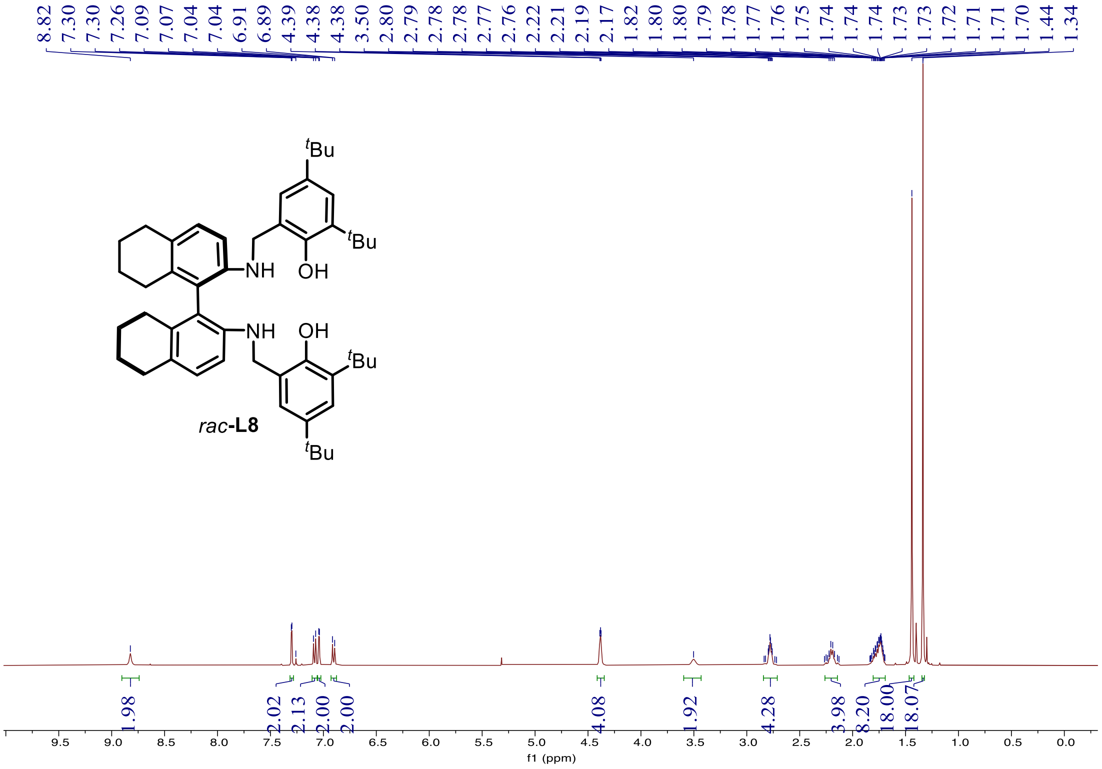

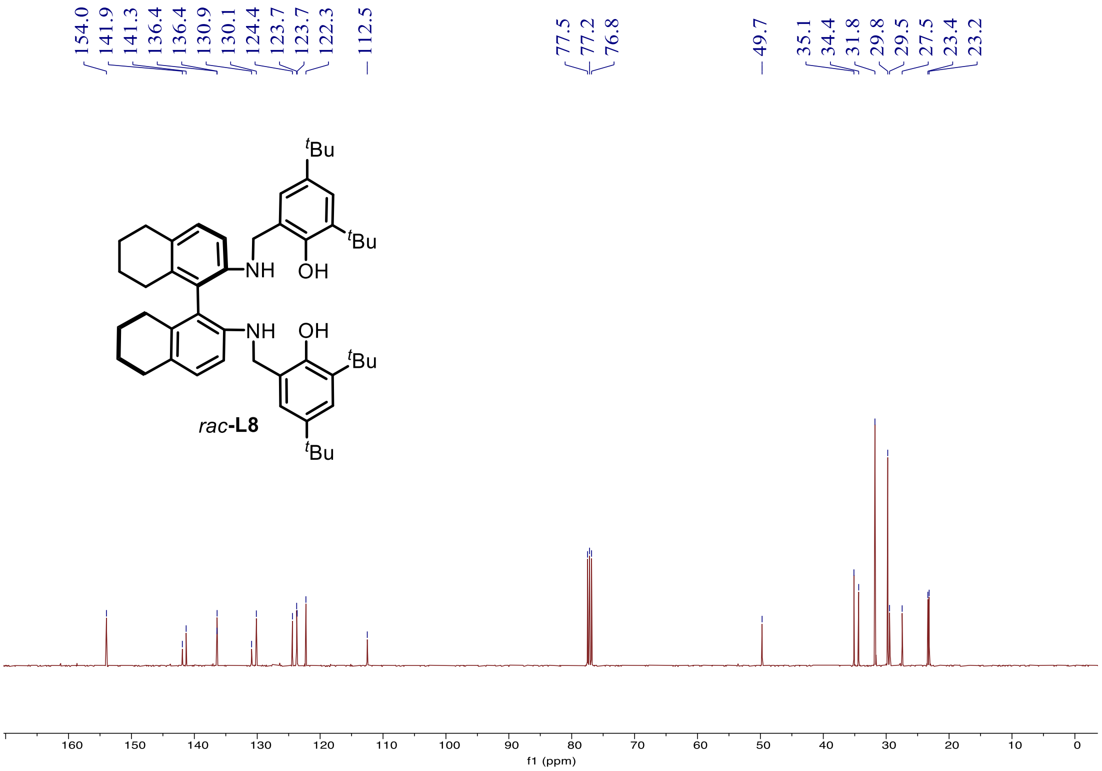

Figure S9. ^{1}H NMR and ^{13}C NMR ($CDCl_3$, 25 °C) spectra of ligand *rac*-**L8**.

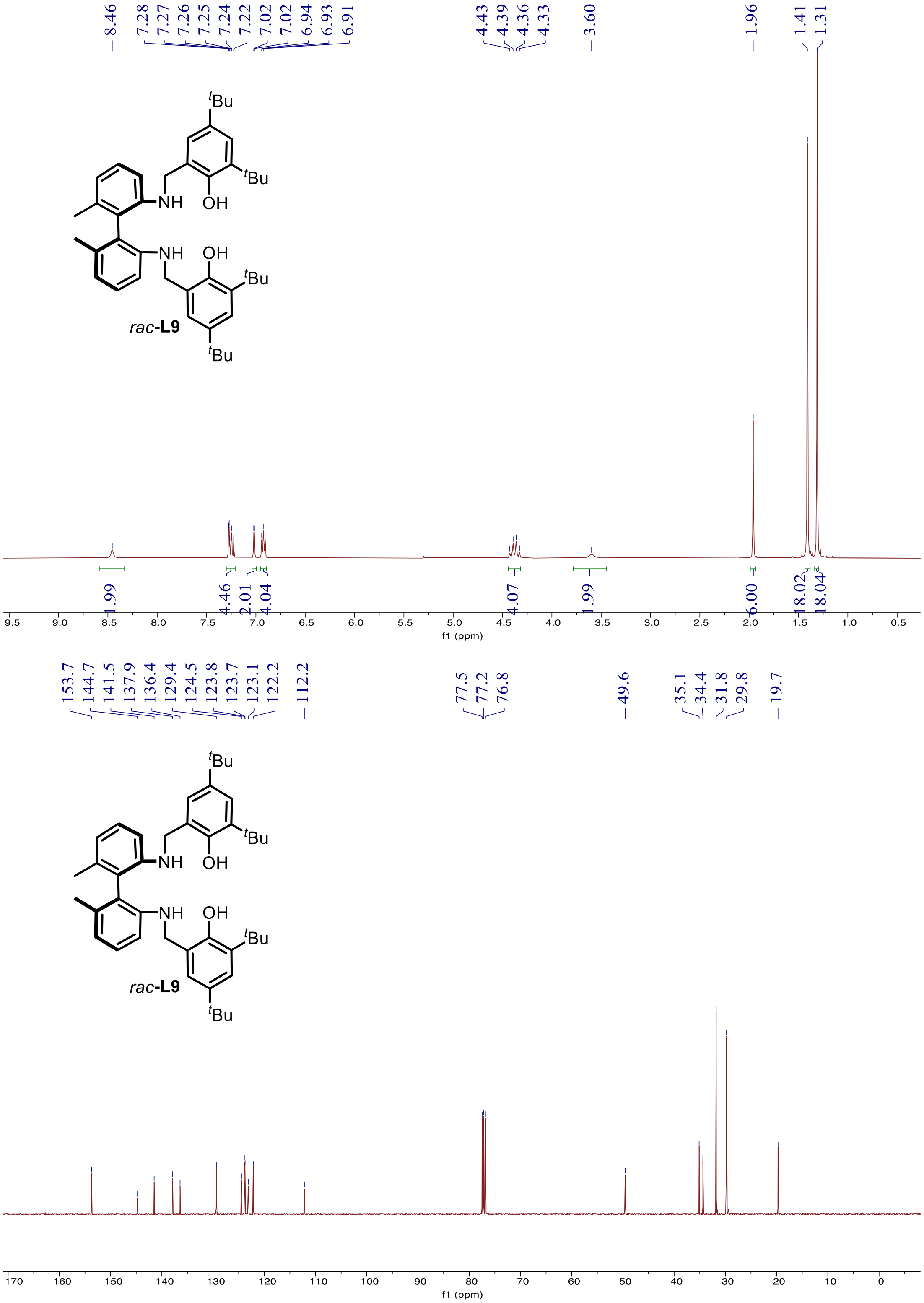

Figure S10. ^{1}H NMR and ^{13}C NMR ($CDCl_3$, 25 °C) spectra of ligand *rac*-**L9**.

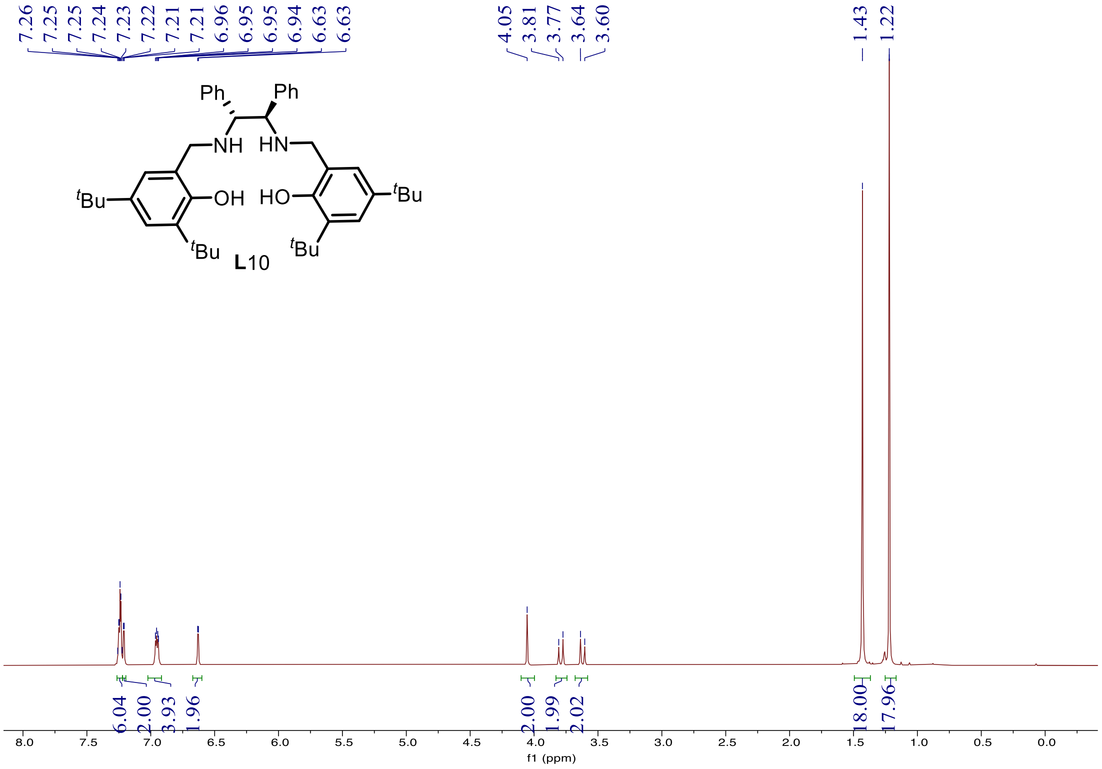

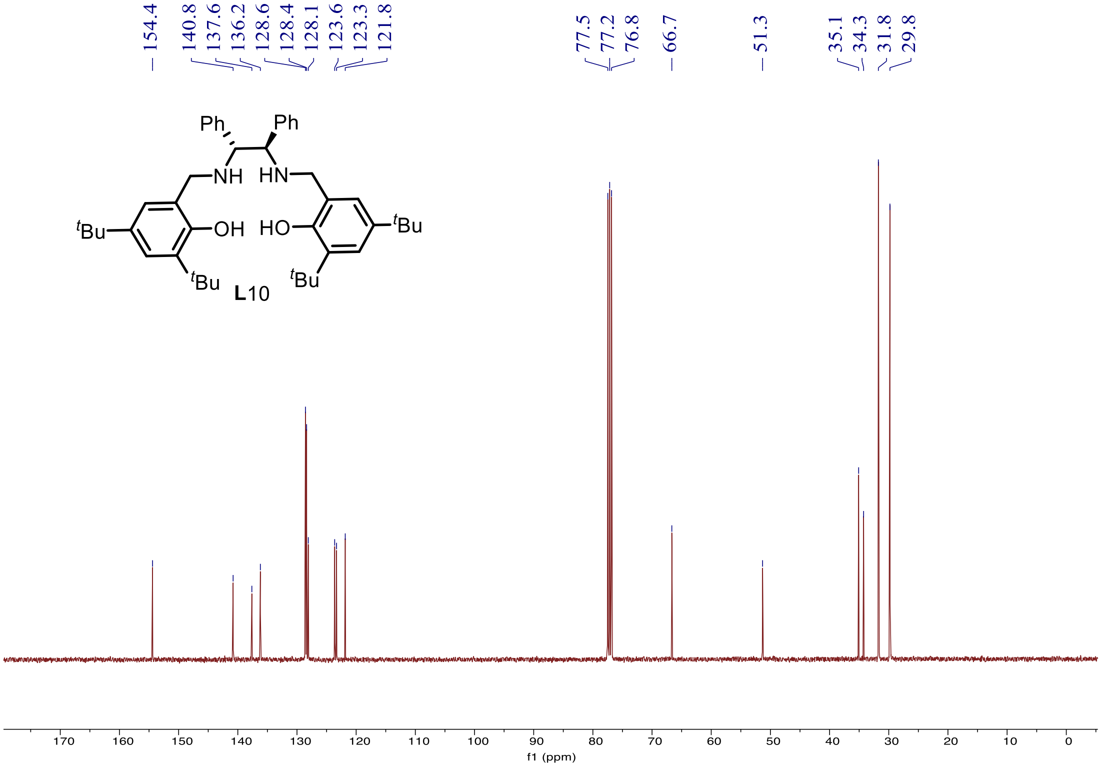

Figure S11. ^{1}H NMR and ^{13}C NMR ($CDCl_3$, 25 °C) spectra of ligand **L10**.

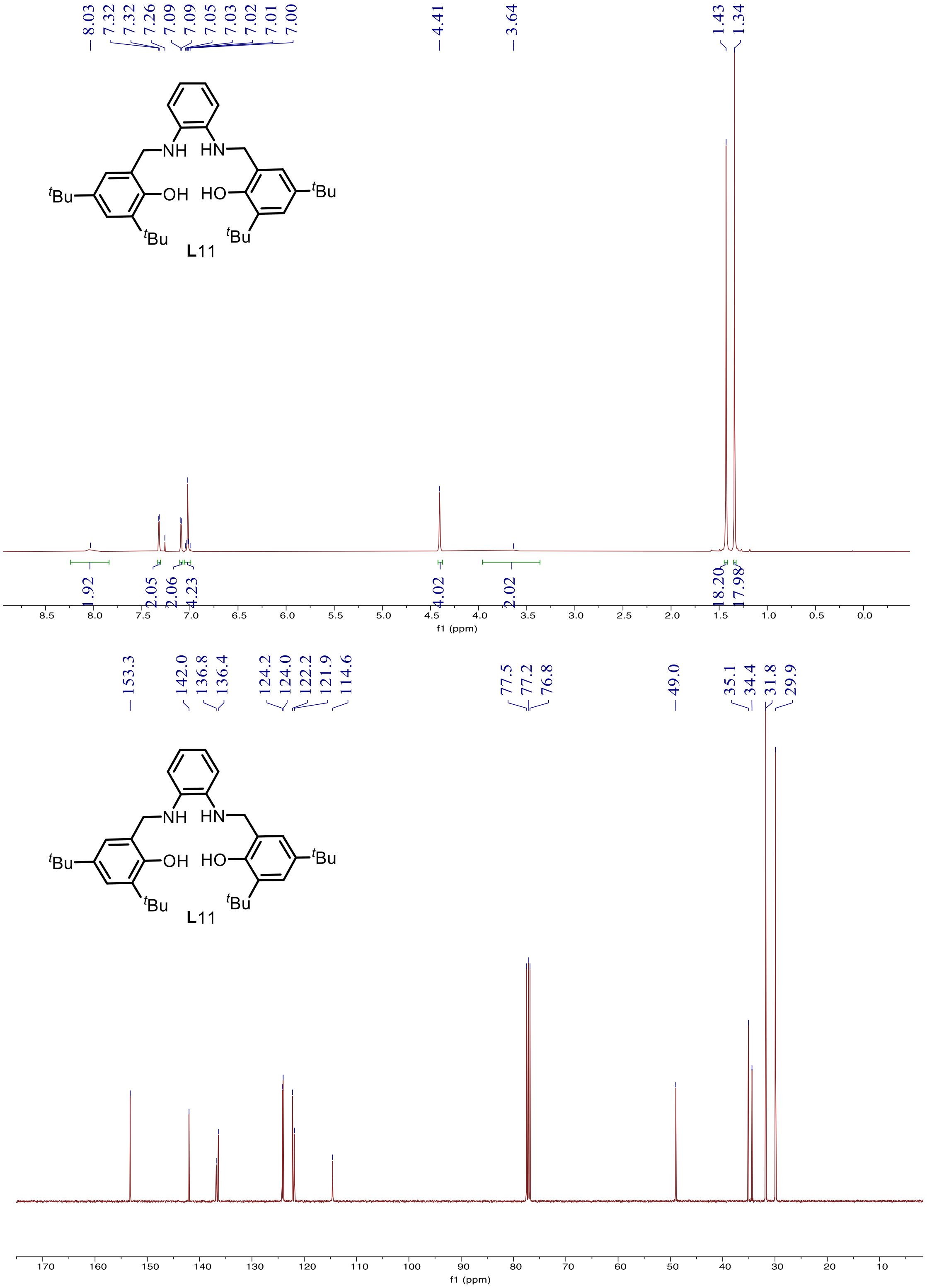

Figure S12. ^{1}H NMR and ^{13}C NMR ($CDCl_3$, 25 °C) spectra of ligand **L11**.

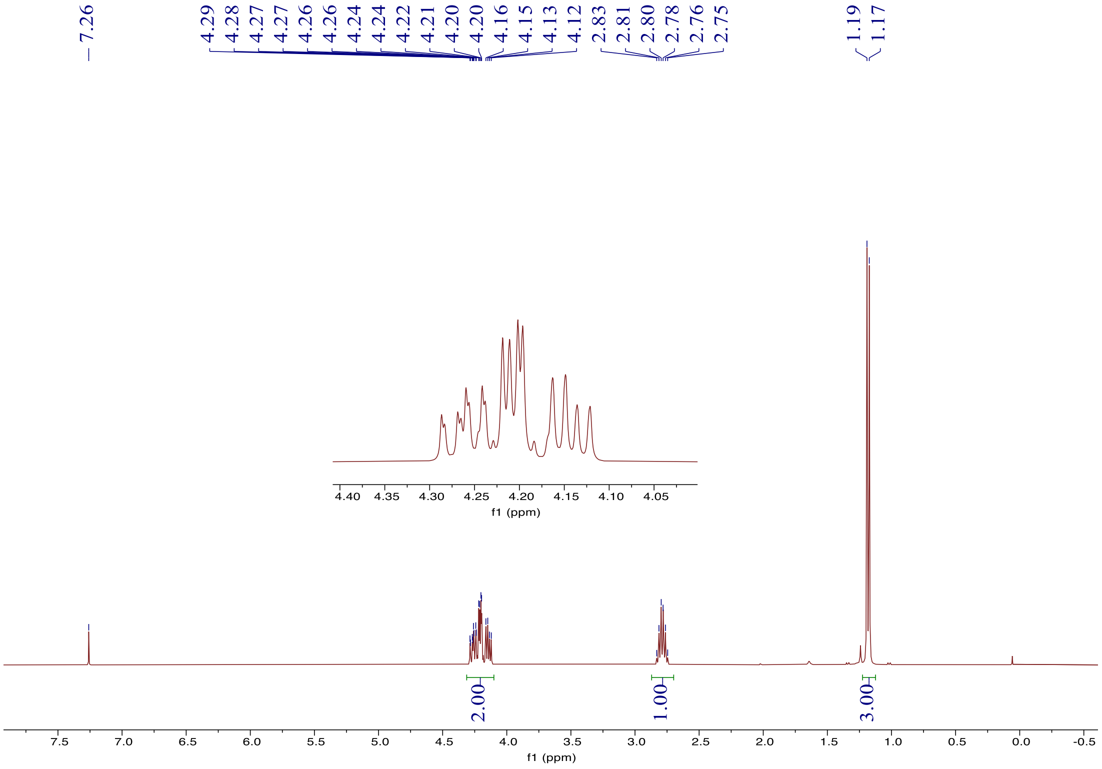

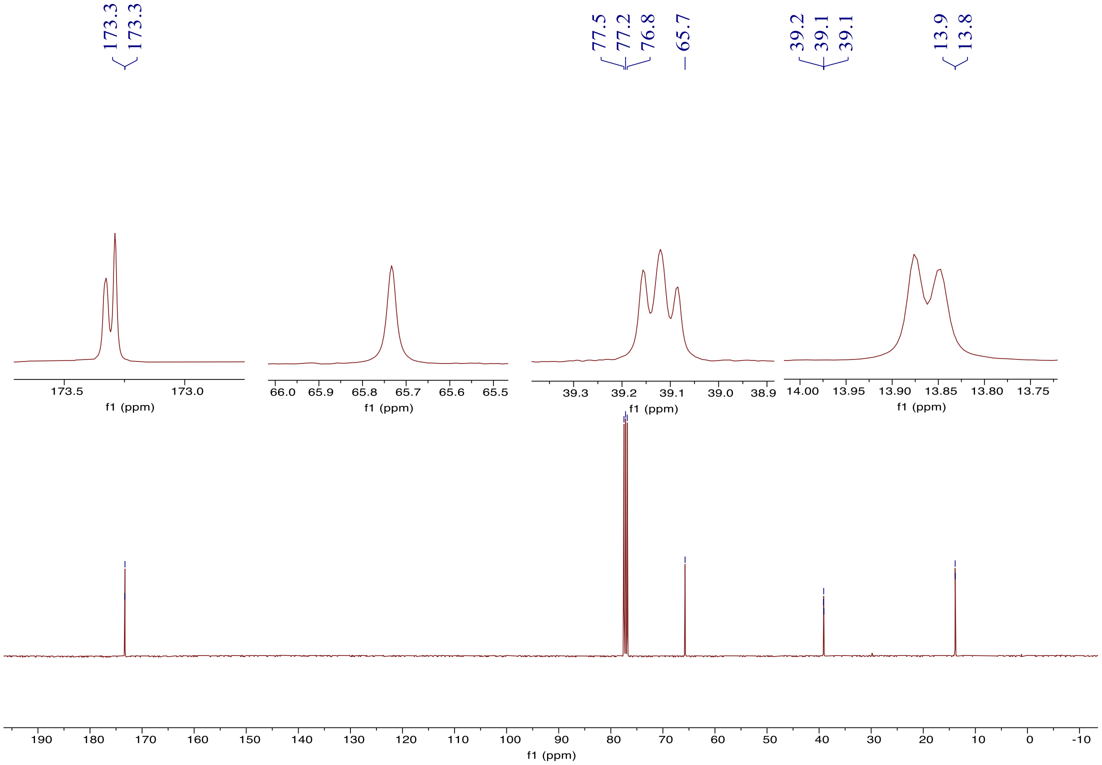

Figure S13. ^{1}H NMR and ^{13}C NMR ($CDCl_3$, 25 °C) spectra of P3H2MP ($P_m = 0.51$).

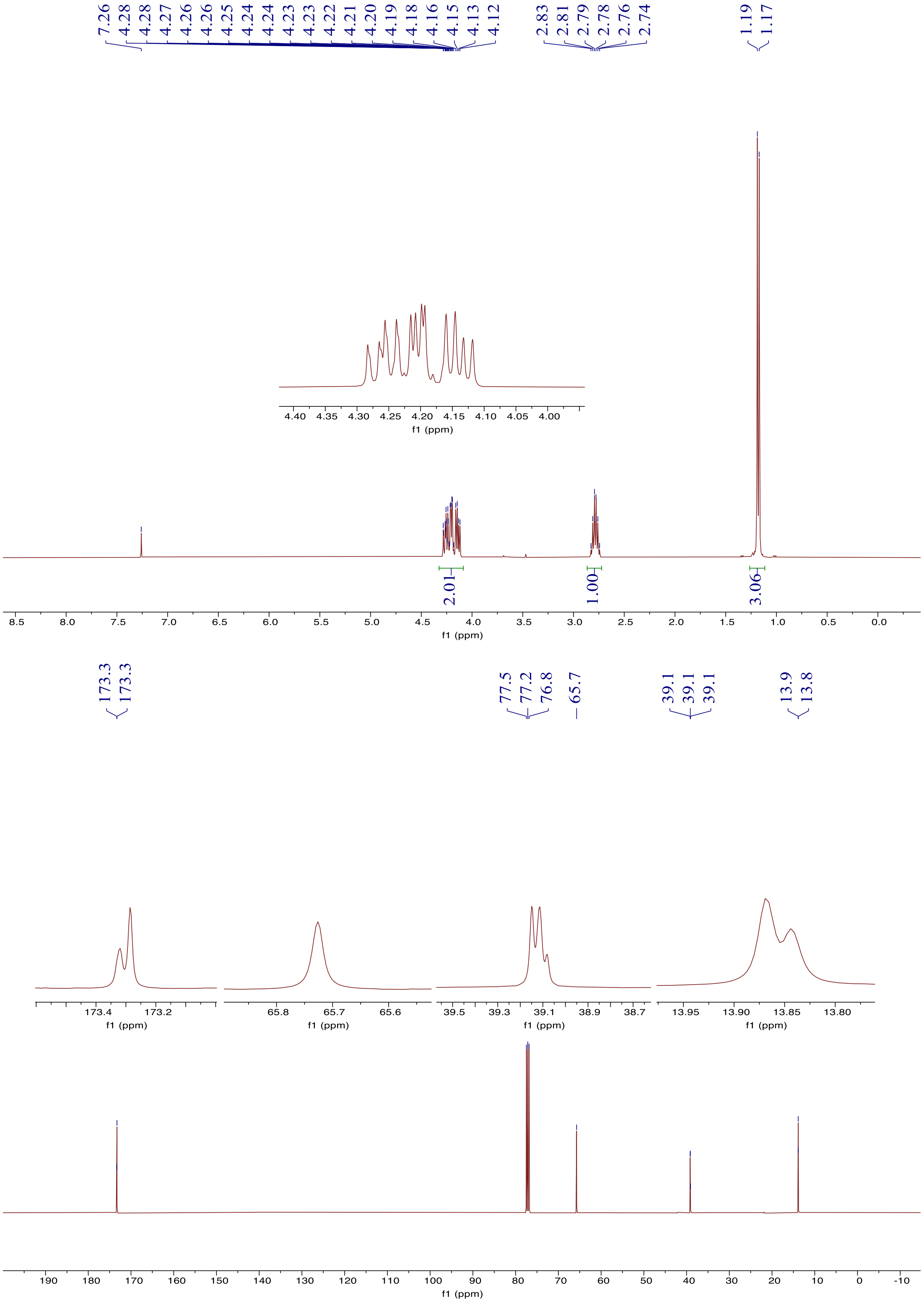

Figure S14. ^{1}H NMR and ^{13}C NMR ($CDCl_3$, 25 °C) spectra of P3H2MP (P_r = 0.61).

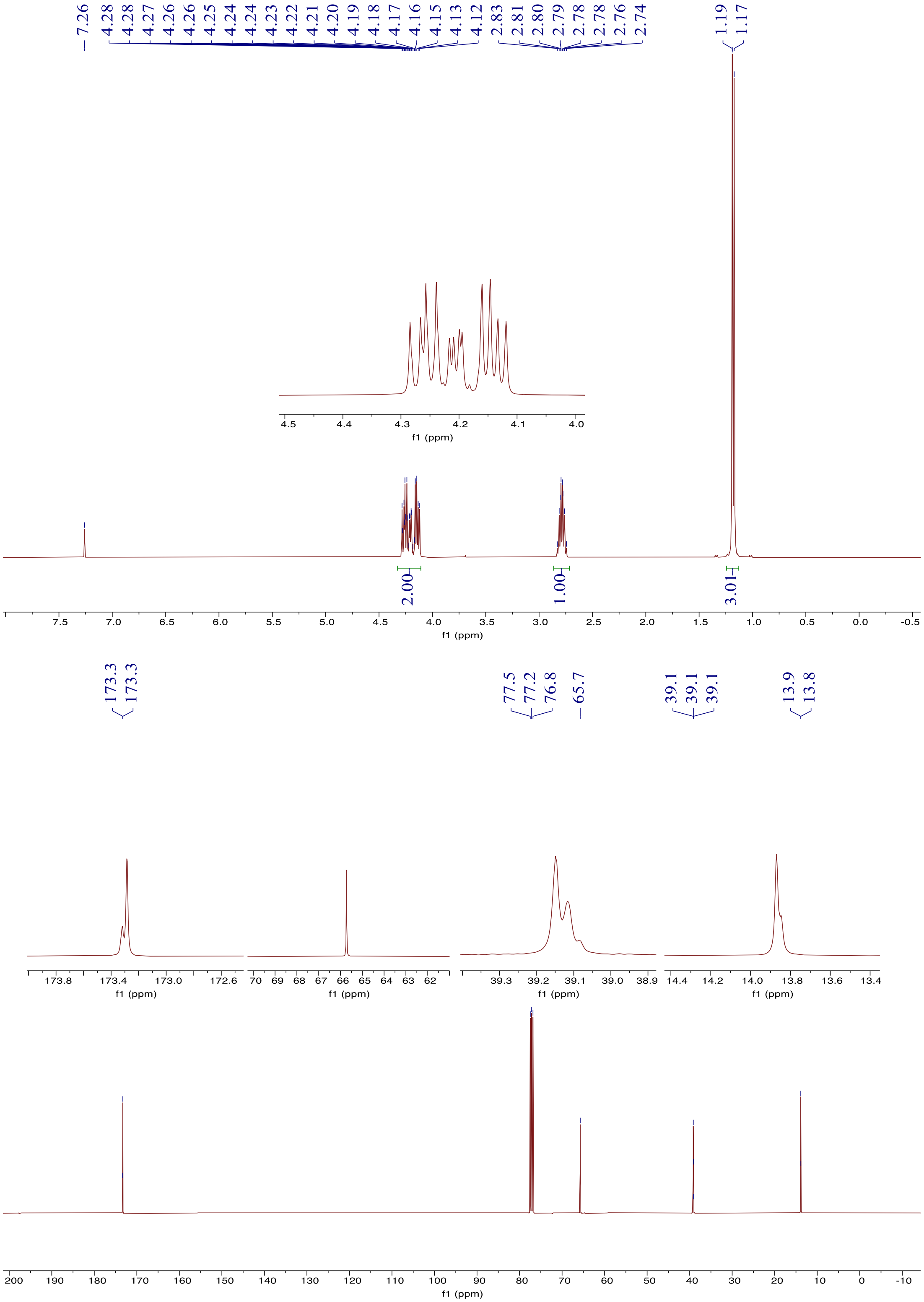

Figure S15. ^{1}H NMR and ^{13}C NMR ($CDCl_3$, 25 °C) spectra of P3H2MP ($P_r = 0.72$).

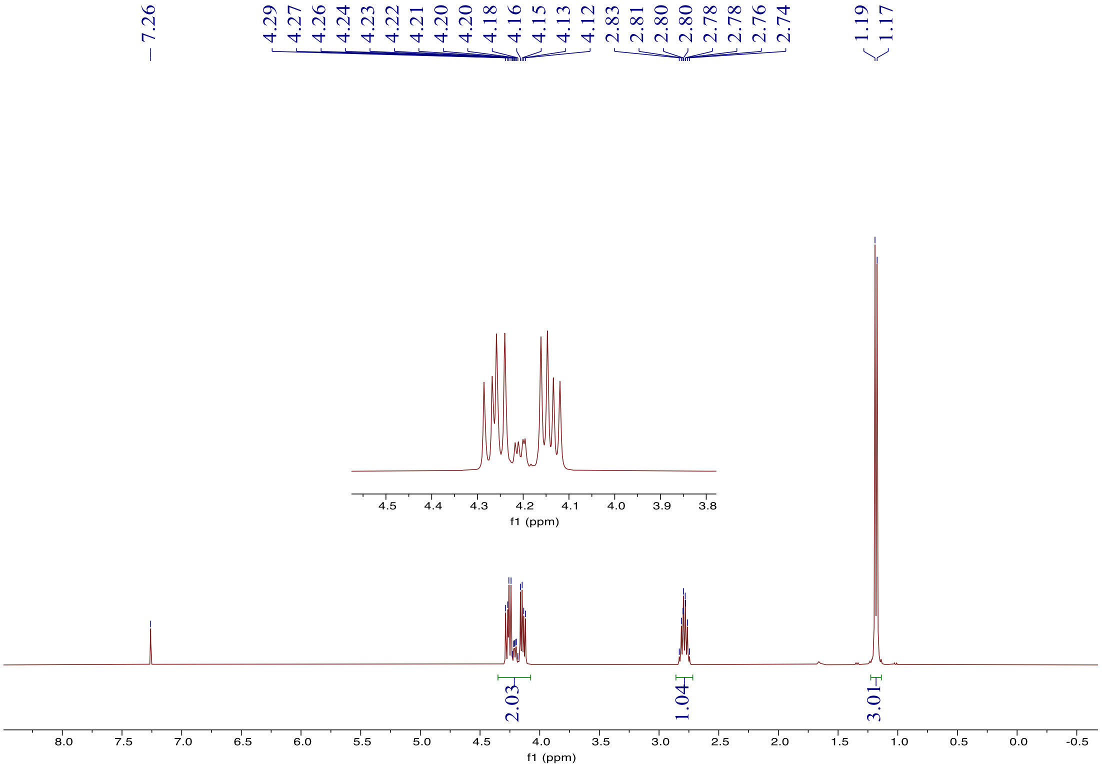

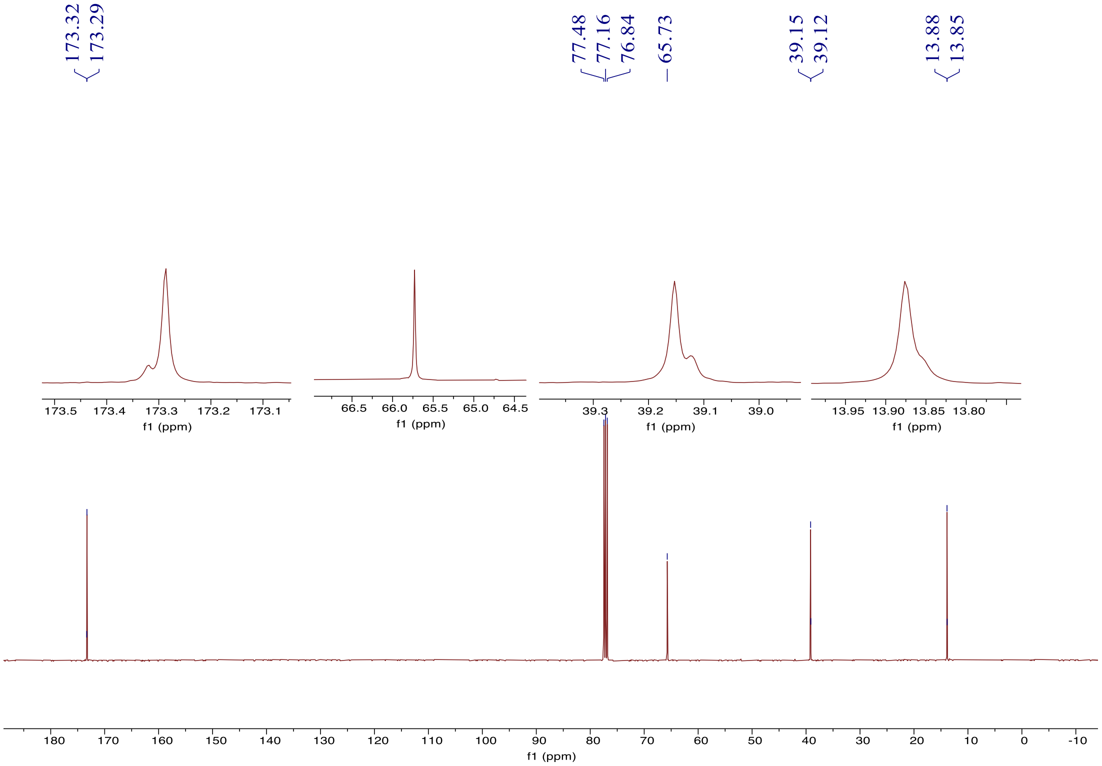

Figure S16. ^{1}H NMR and ^{13}C NMR ($CDCl_3$, 25 °C) spectra of P3H2MP (P_r = 0.80).

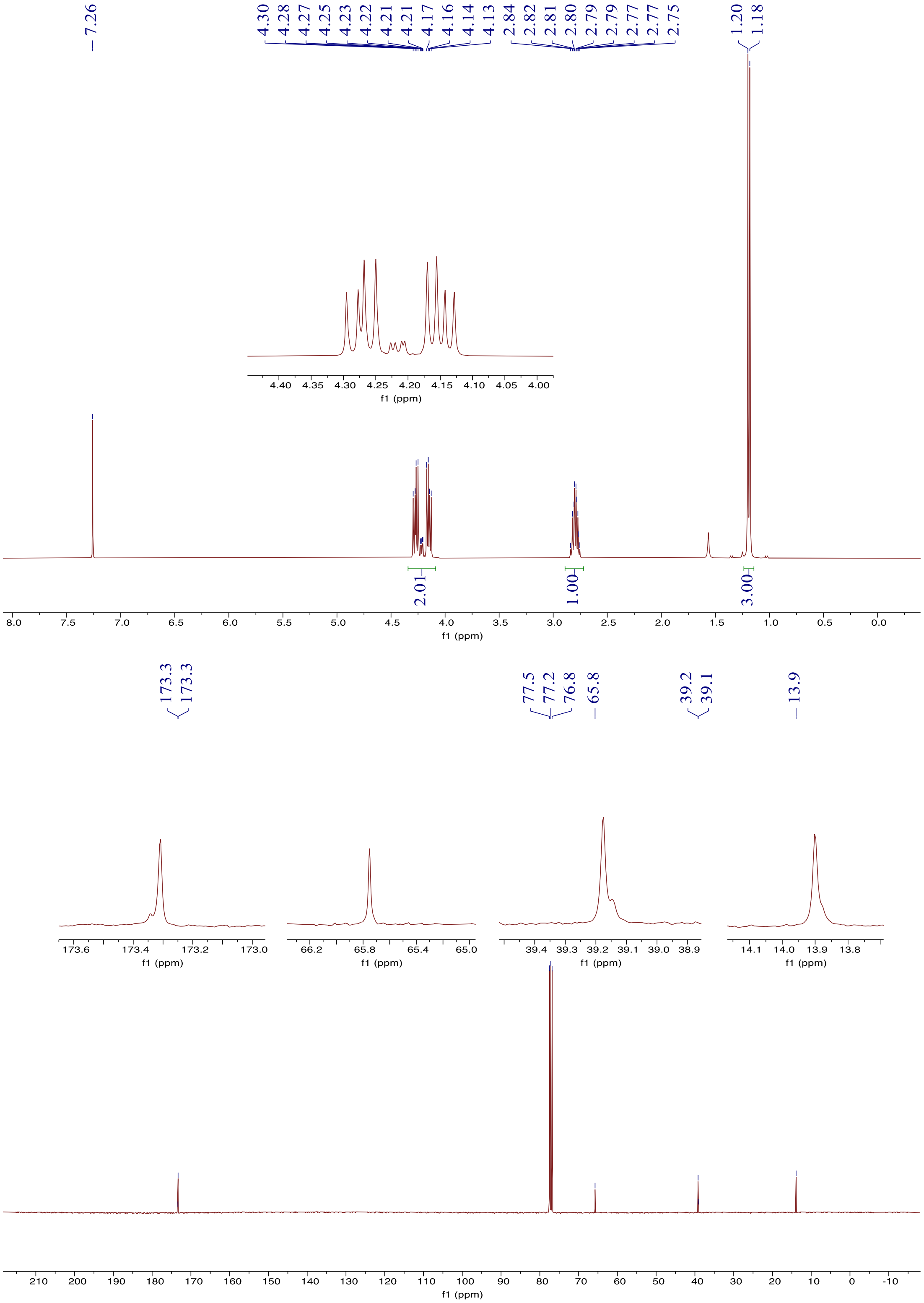

Figure S17. ^{1}H NMR and ^{13}C NMR ($CDCl_3$, 25 °C) spectra of P3H2MP (P_r = 0.88).

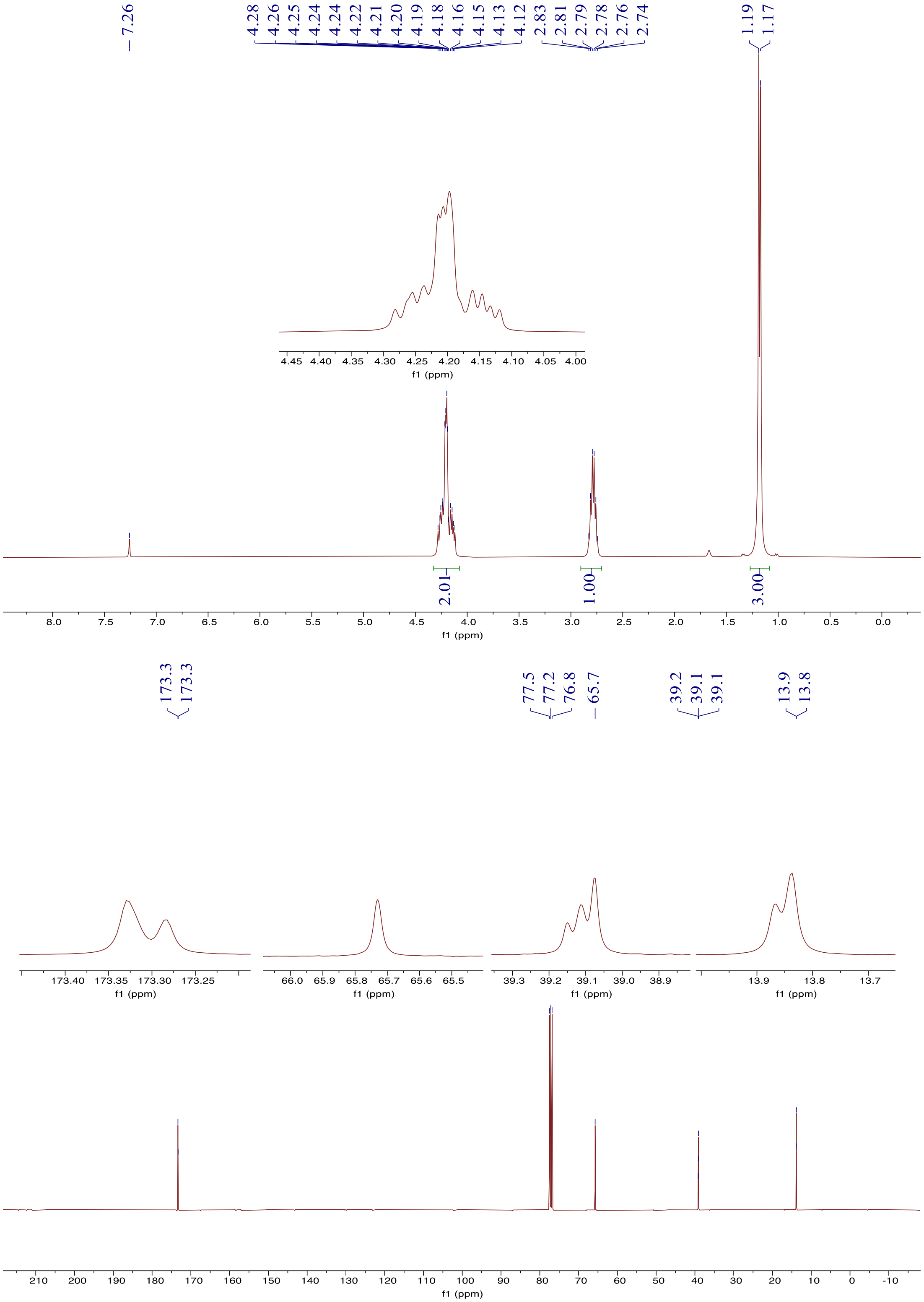

Figure S18. ^{1}H NMR and ^{13}C NMR ($CDCl_3$, 25 °C) spectra of P3H2MP ($P_m = 0.62$).

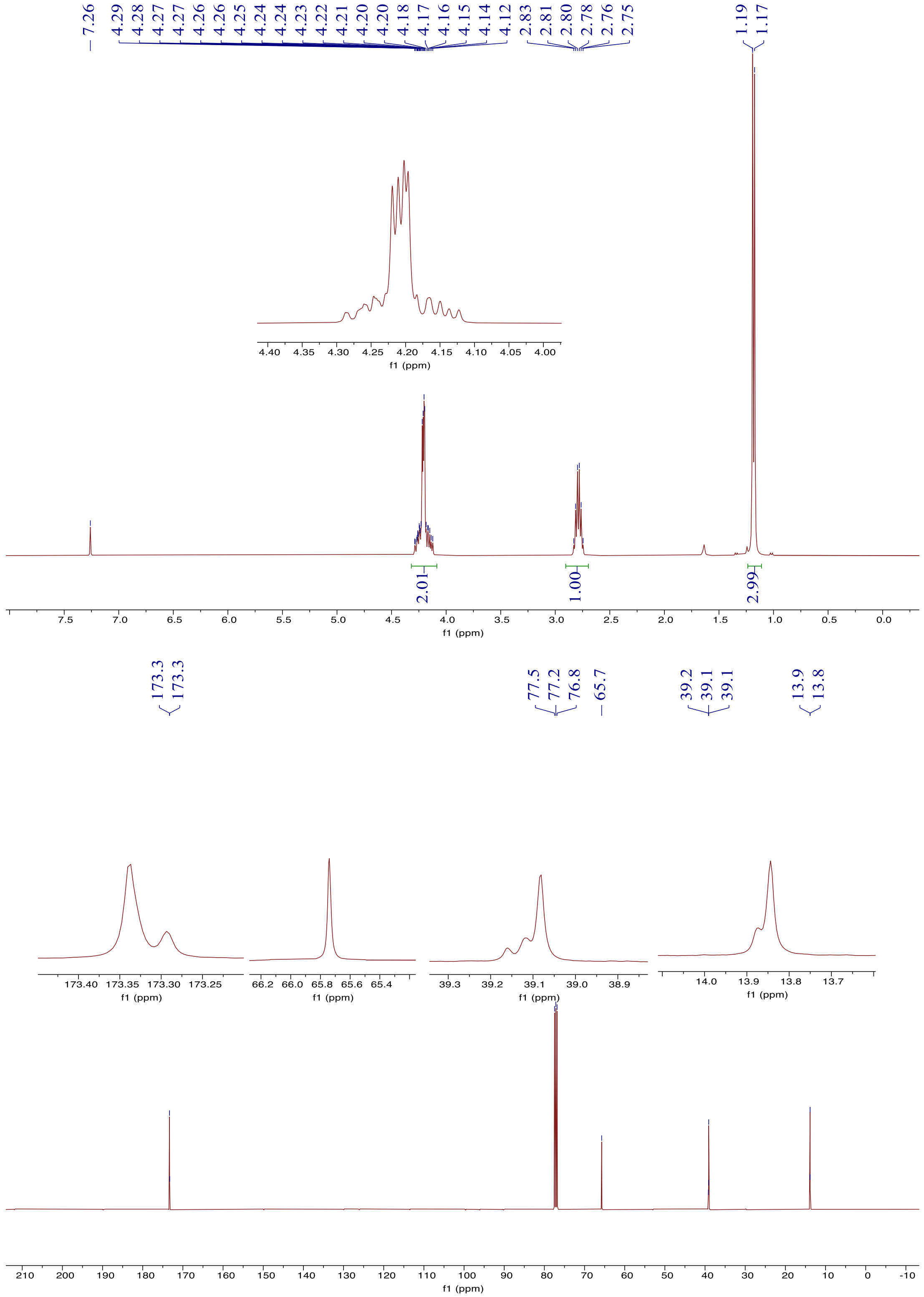

Figure S19. ^{1}H NMR and ^{13}C NMR ($CDCl_3$, 25 °C) spectra of P3H2MP (P_m = 0.75).

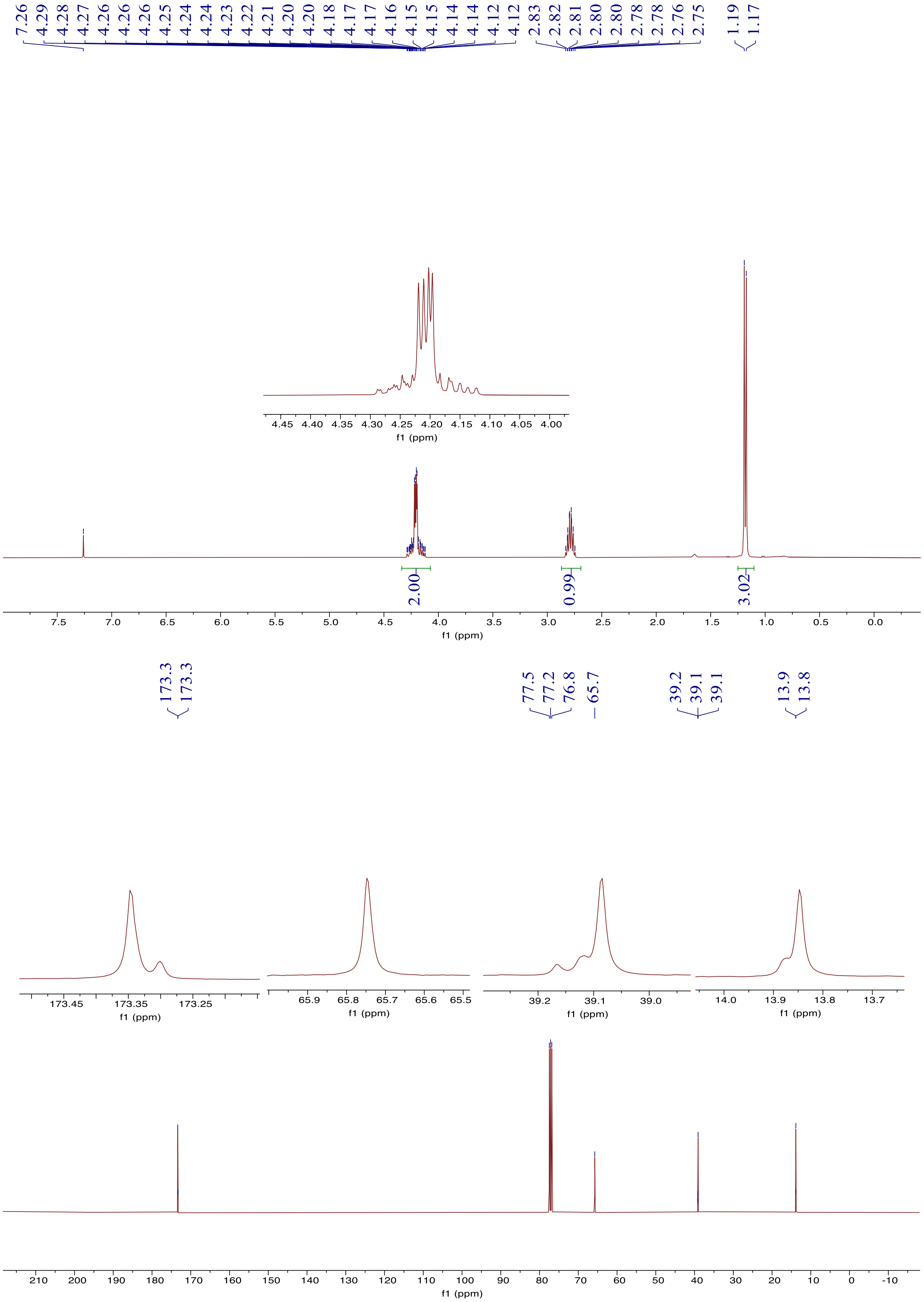

Figure S20. ^{1}H NMR and ^{13}C NMR ($CDCl_3$, 25 °C) spectra of P3H2MP (P_m = 0.80).

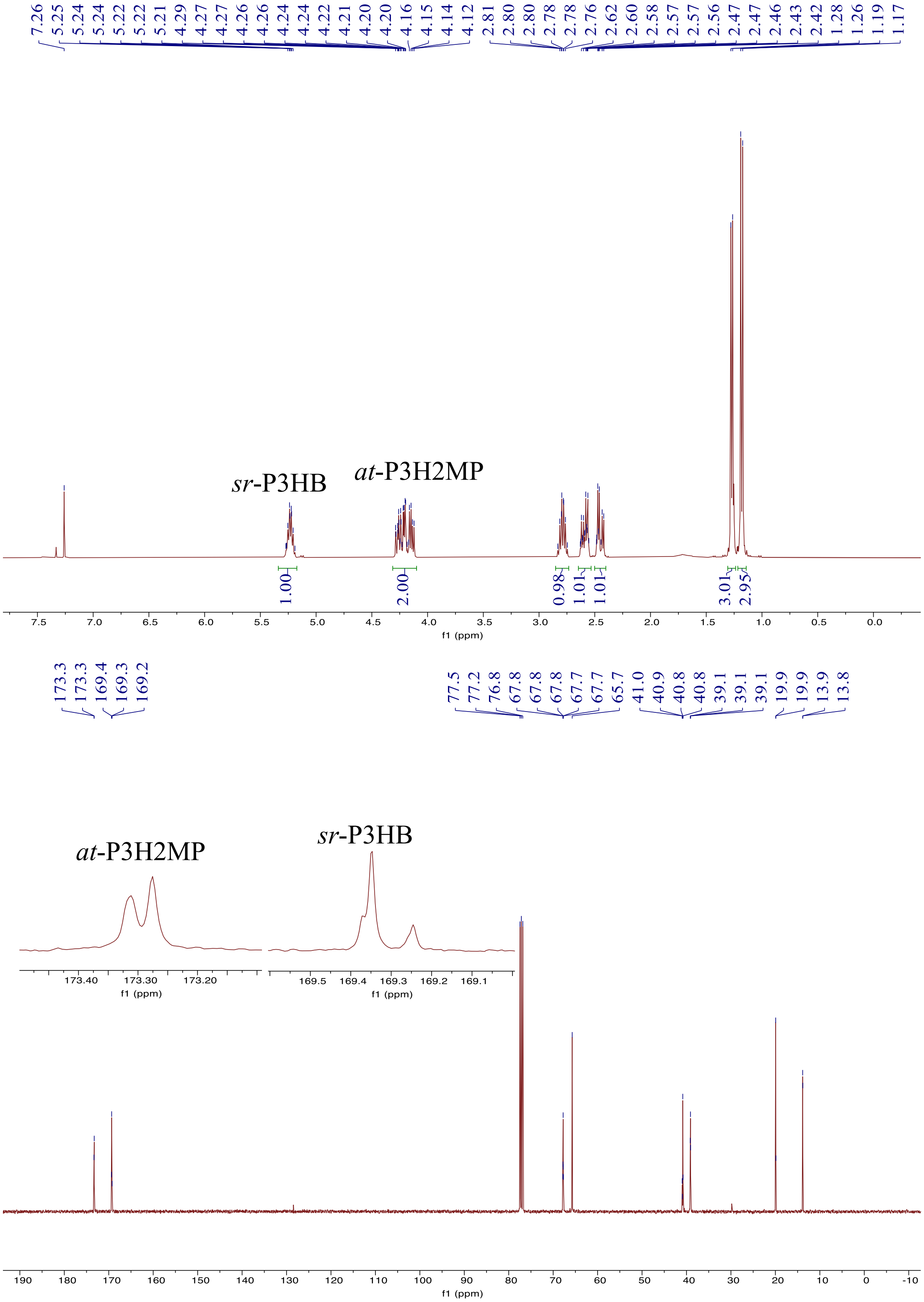

Figure S21. ^{1}H NMR and ^{13}C NMR ($CDCl_3$, 25 °C) spectra of triblock copolymer *sr*-P3HB$_{50}$−*at*-P3H2MP$_{100}$−*sr*-P3HB$_{50}$.

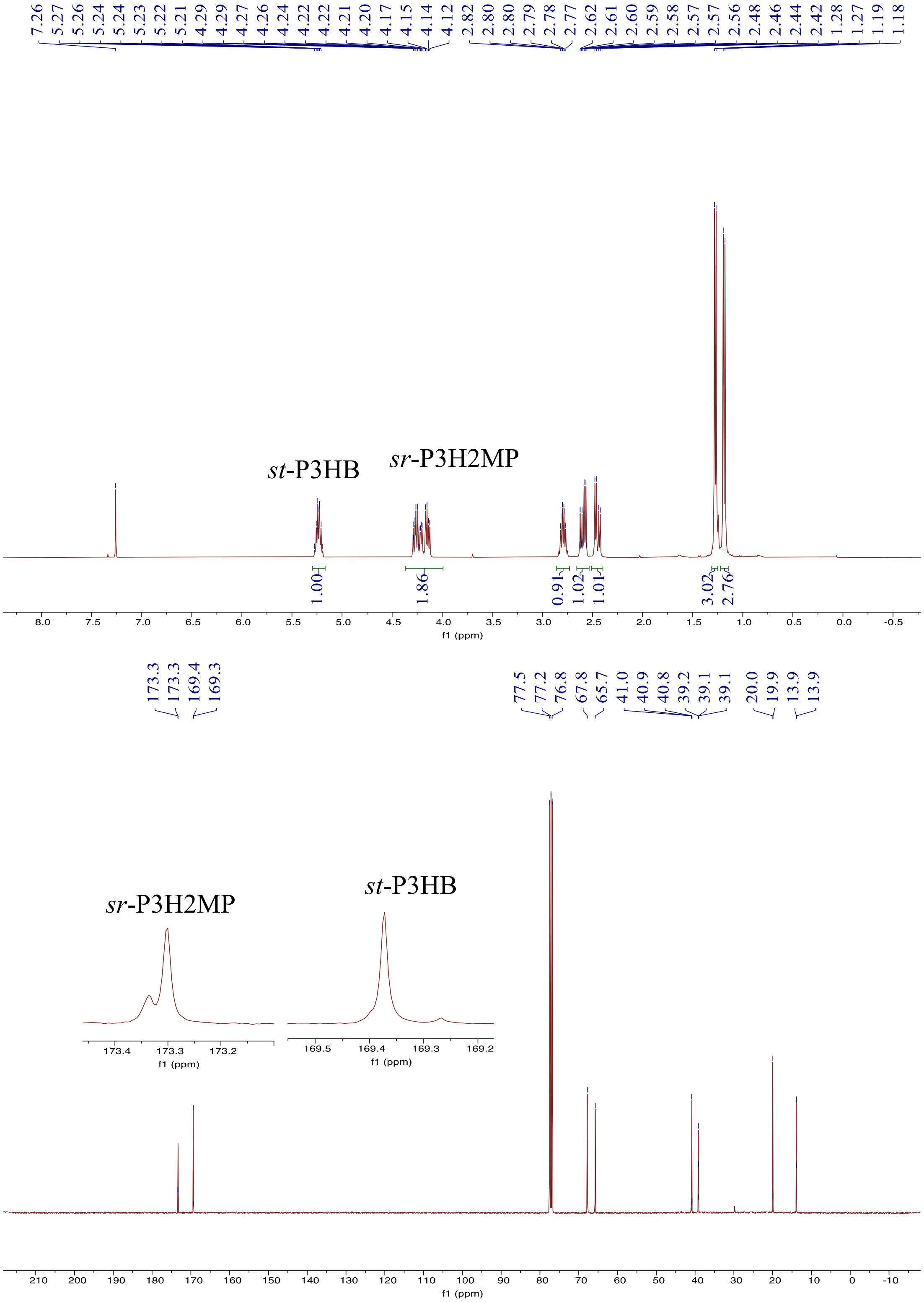

Figure S22. ^{1}H NMR and ^{13}C NMR ($CDCl_3$, 25 °C) spectra of triblock copolymer *st*-P3HB$_{50}$–*sr*-P3H2MP$_{100}$–*st*-P3HB$_{50}$.

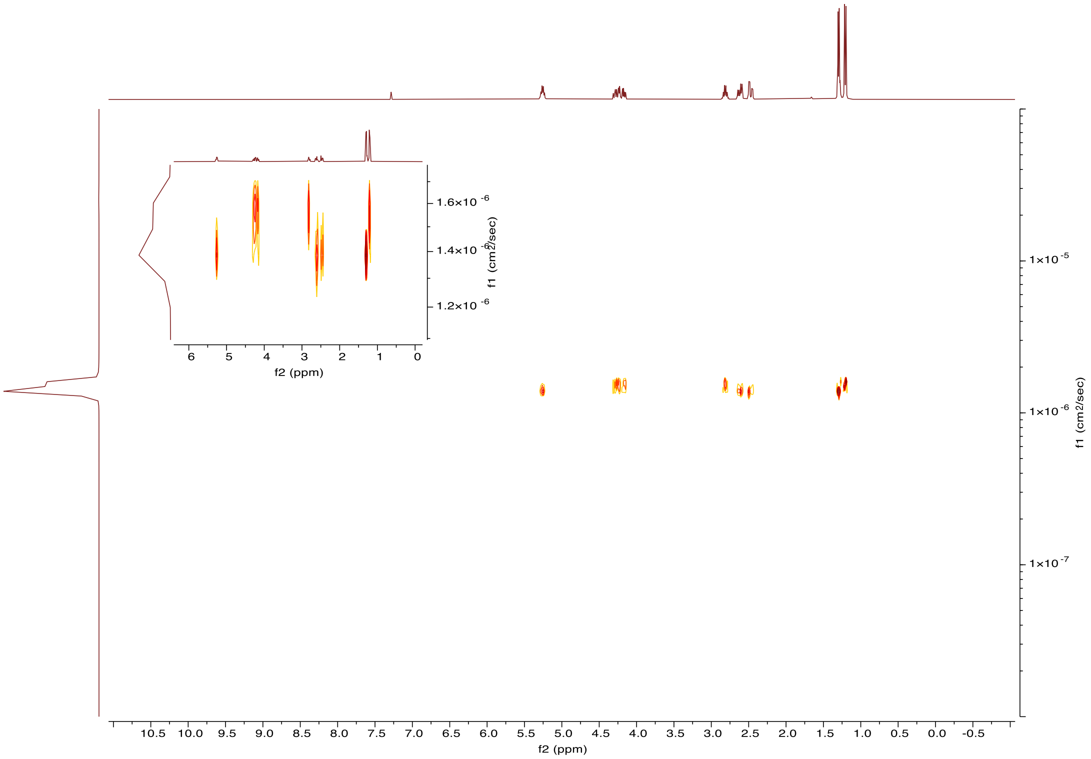

Figure S23. DOSY ^{1}H NMR spectra of the physical blend of P3H2MP and P3HB.

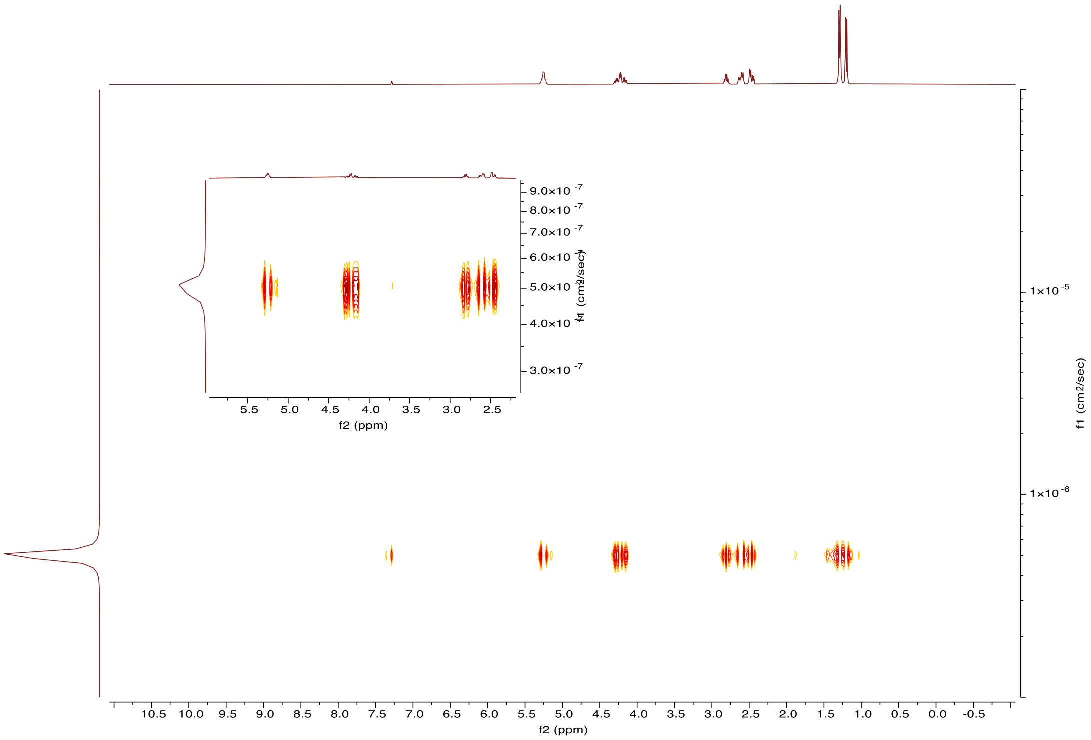

Figure S24. DOSY ^{1}H NMR spectrum of triblock copolymer *sr*-P3HB$_{50}$–*at*-P3H2MP$_{100}$–*sr*-P3HB$_{50}$ prepared with **Y1**.

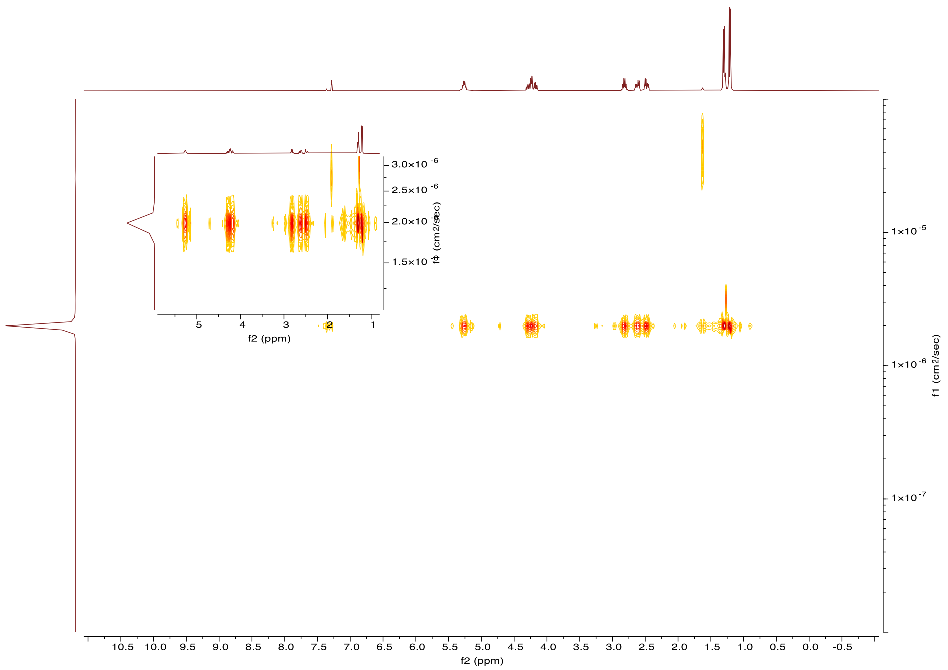

Figure S25. DOSY ^{1}H NMR spectrum of triblock copolymer *sr*-P3HB$_{50}$–*at*-P3H2MP$_{100}$–*sr*-P3HB$_{50}$ prepared with **Y2**.

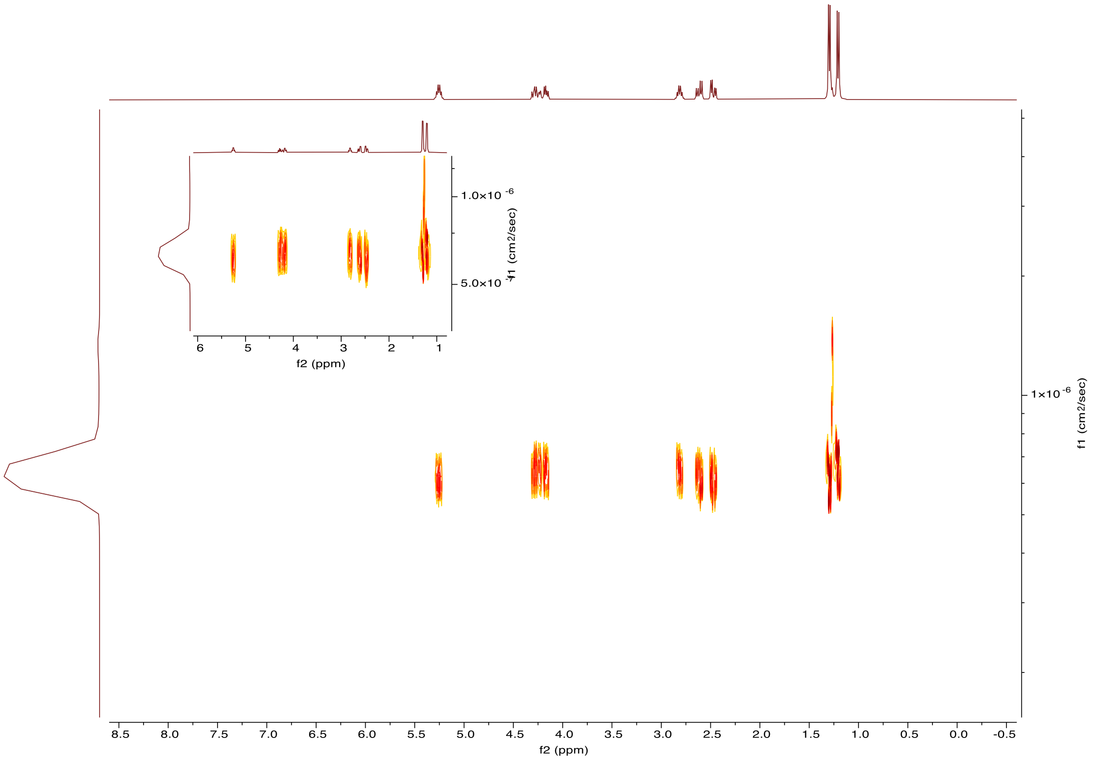

Figure S26. DOSY ^{1}H NMR spectrum of triblock copolymer *st*-P3HB$_{50}$–*sr*-P3H2MP$_{100}$–*st*-P3HB$_{50}$ prepared with **Y3**.

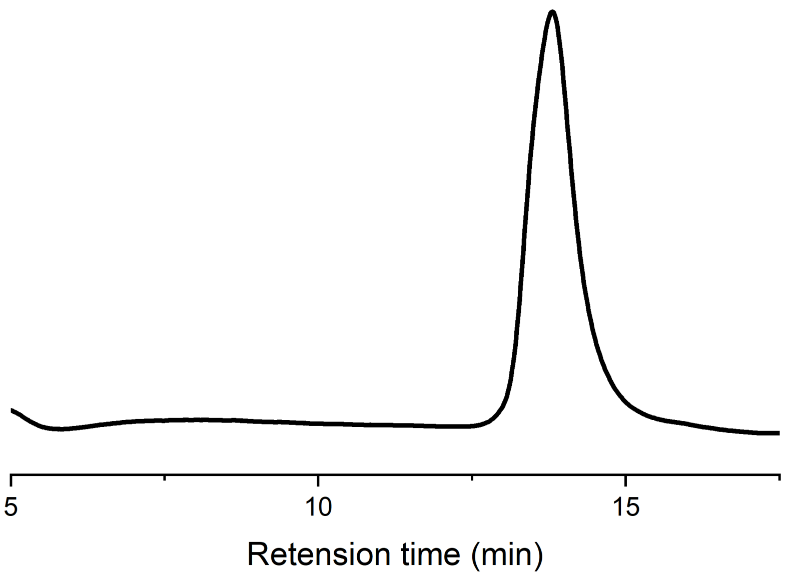

Figure S27. SEC trace of P3H2MP (M_n = 189 kDa, $Đ$ = 1.07, P_r = 0.70).

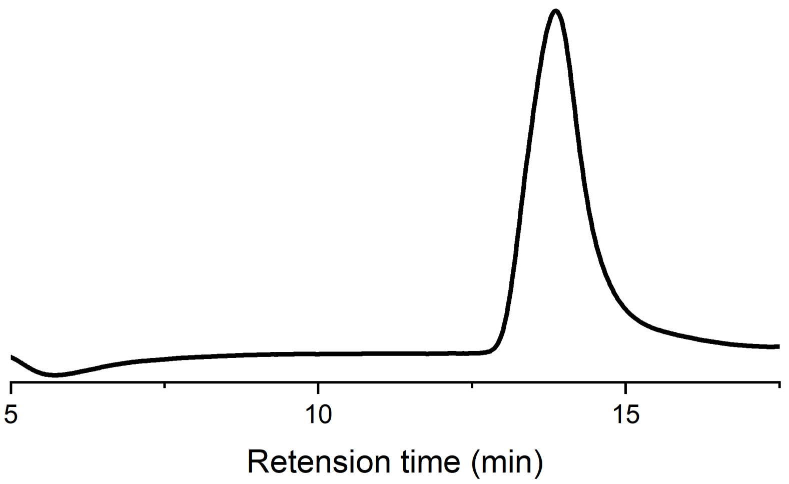

Figure S28. SEC trace of P3H2MP (M_n = 175 kDa, $Đ$ = 1.09, P_r = 0.88).

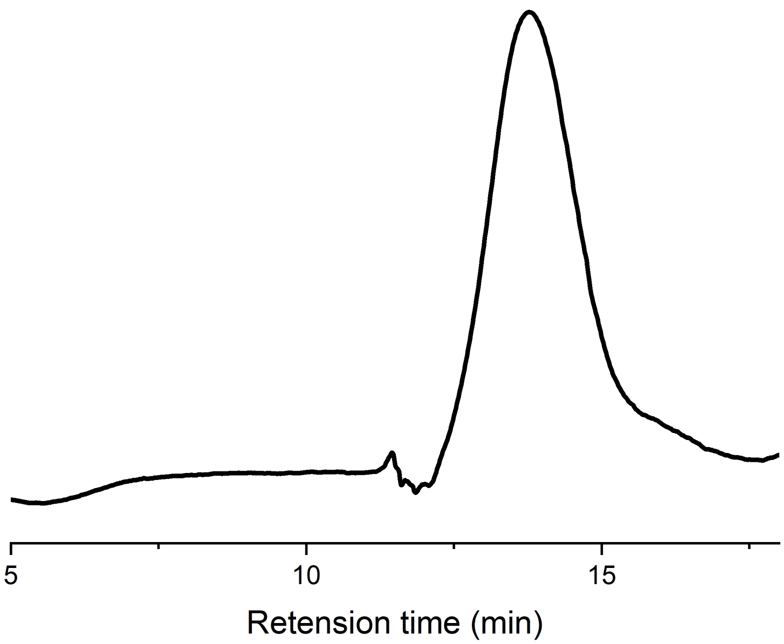

Figure S29. SEC trace of P3H2MP (M_n = 419 kDa, $Đ$ = 1.79, P_m = 0.74).

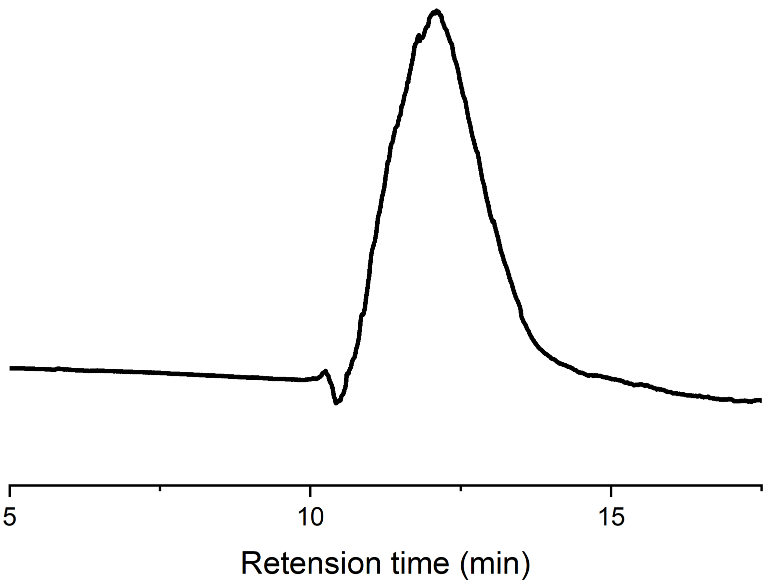

Figure S30. SEC trace of P3H2MP (M_n = 973 kDa, $Đ$ = 1.42, P_m = 0.80).

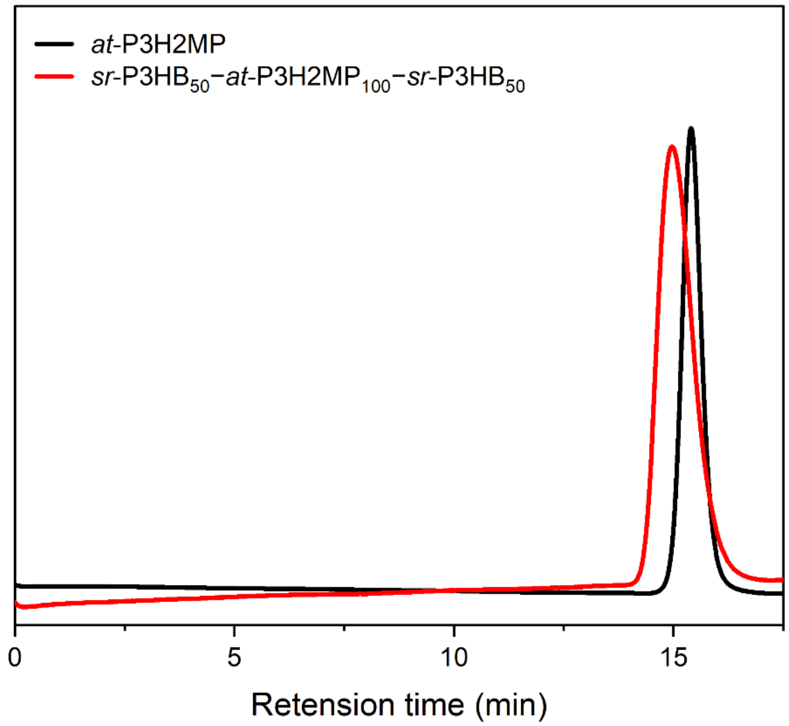

Figure S31. SEC traces of *at*-P3H2MP macroinitiator (M_n = 19.8 kDa, $Đ$ = 1.03) and *sr*-P3HB$_{50}$–*at*-P3H2MP$_{100}$–*sr*-P3HB$_{50}$ triblock copolymer (M_n = 29.6 kDa, $Đ$ = 1.19) prepared with **Y1** in one pot.

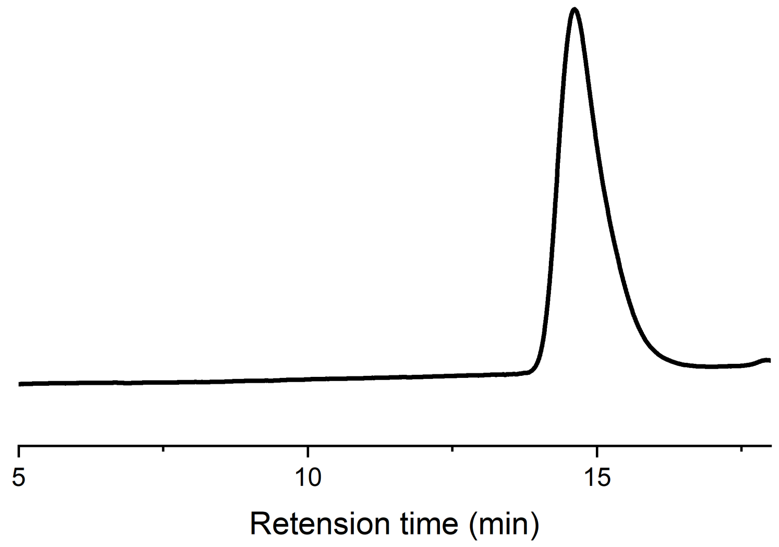

Figure S32. SEC trace of *sr*-P3HB$_{50}$–*at*-P3H2MP$_{100}$–*sr*-P3HB$_{50}$ prepared with [*rac*-MPL]/[*rac*-BBL]/[**Y2**]/[BDM] = 100:100:1:0.5 (M_n = 23.8 kDa, $Đ$ = 1.30).

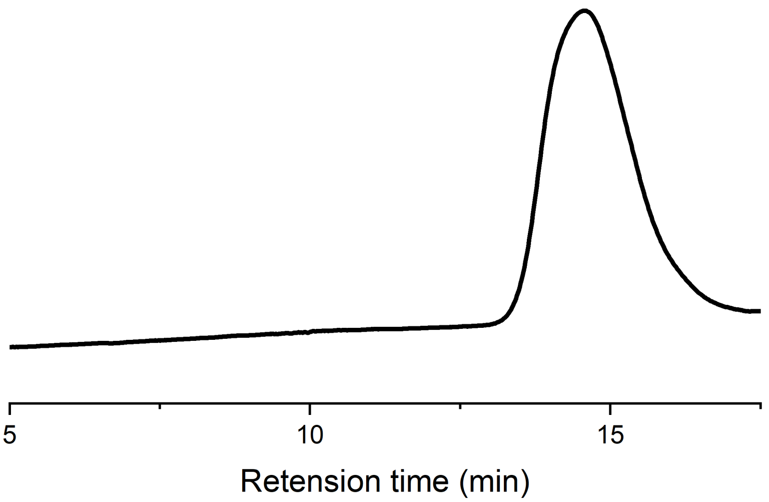

Figure S33. SEC trace of *st*-P3HB$_{50}$–*sr*-P3H2MP$_{100}$–*st*-P3HB$_{50}$ prepared with [*rac*-MPL]/[*rac*-BBL]/[**Y3**]/[BDM]= 100:100:1:0.5 (M_n = 26.9 kDa, *Đ* = 1.34).

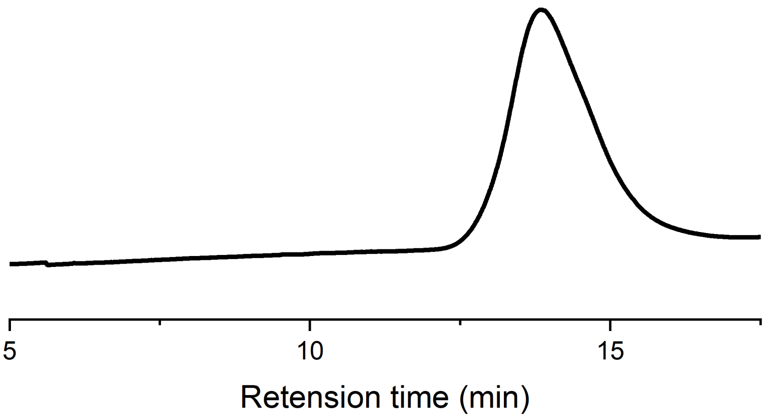

Figure S34. SEC trace of P(*st*-3HB$_{50}$-*co*-3H2MP$_{50}$) prepared with [*rac*-MPL]/[*rac*-BBL]/[*rac*-**L2**+**Y6**] = 100:100:1 (M_n = 56.4 kDa, *Đ* = 1.49).

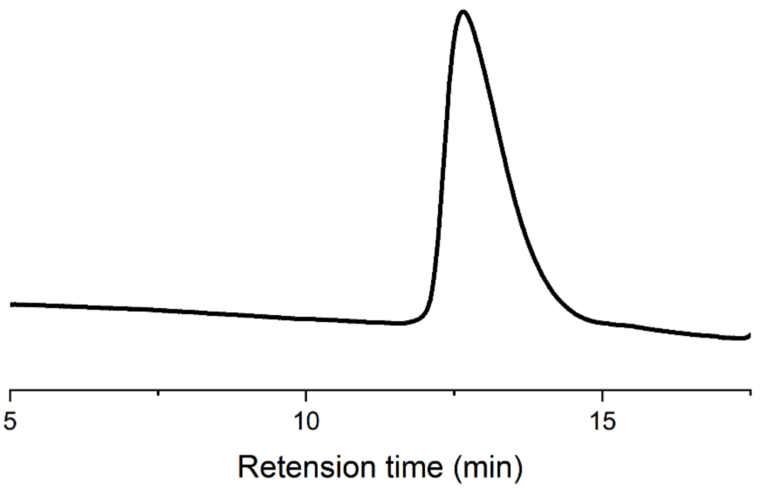

Figure S35. SEC trace of P(*st*-3HB$_{72}$-*co*-3H2MP$_{28}$) prepared with [*rac*-MPL]/[*rac*-BBL]/[*rac*-**L2**+**Y6**] = 2000:500:1 (M_n = 293 kDa, $Đ$ = 1.18).

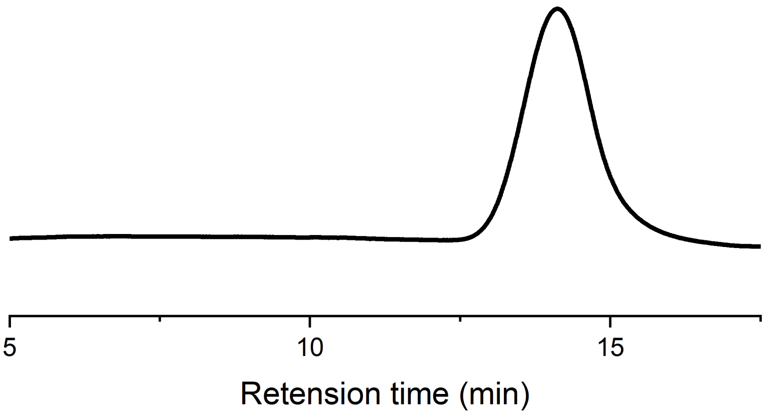

Figure S36. SEC trace of P(*R*-3HB$_{47}$-*co*-3H2MP$_{53}$) prepared with [*rac*-MPL]/[(*R*)-BBL]/[*rac*-**L1**+**Y6**] = 100:100:1 (M_n = 50.1 kDa, $Đ$ = 1.25).

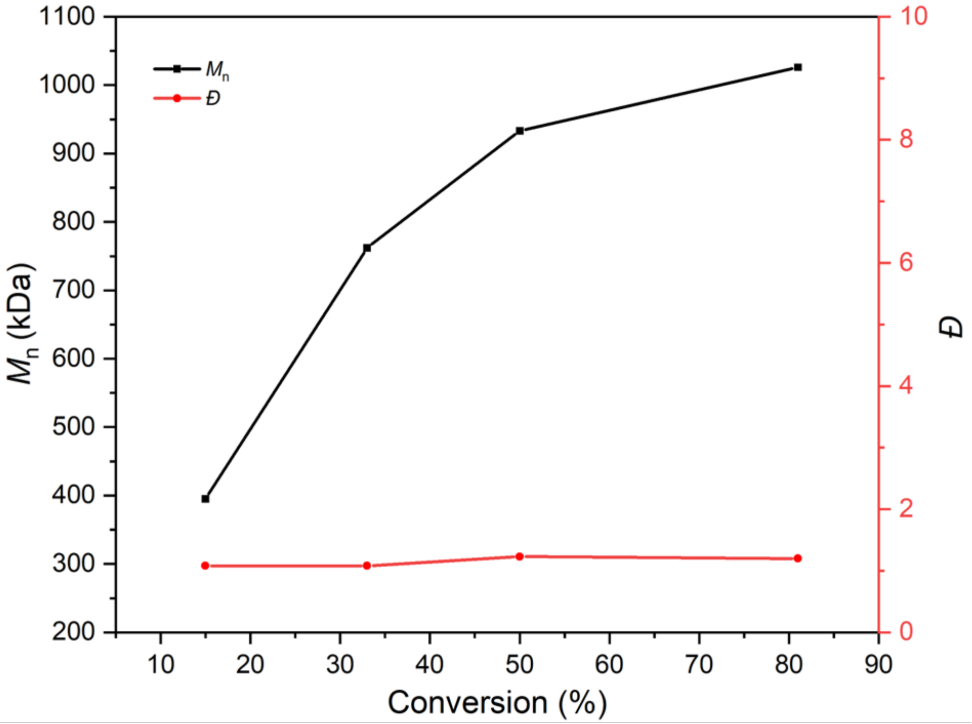

Entry	Conversion (%)	M_n (kDa)	*Đ*
1	15	395	1.08
2	33	762	1.08
3	50	933	1.23
4	81	1026	1.20

Figure S37. Molar mass (M_n) and dispersity (*Đ*) of *ir*-P3H2MP at various monomer conversions ([*rac*-MPL]:[**L6**+**Y6**] = 200:1 at 23 °C), showing controlled chain propagation (*Đ* = 1.08–1.23) with low catalyst initiation efficiency.

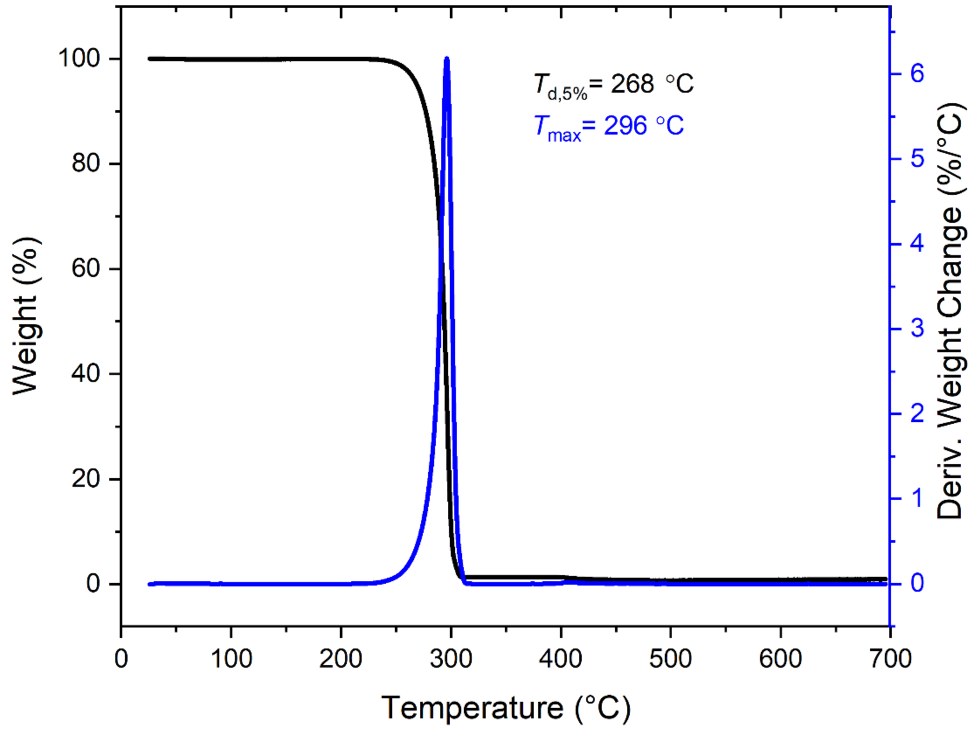

Figure S38. TGA and DTG curves of P3H2MP (M_n = 189 kDa, $Đ$ = 1.07, P_r = 0.70).

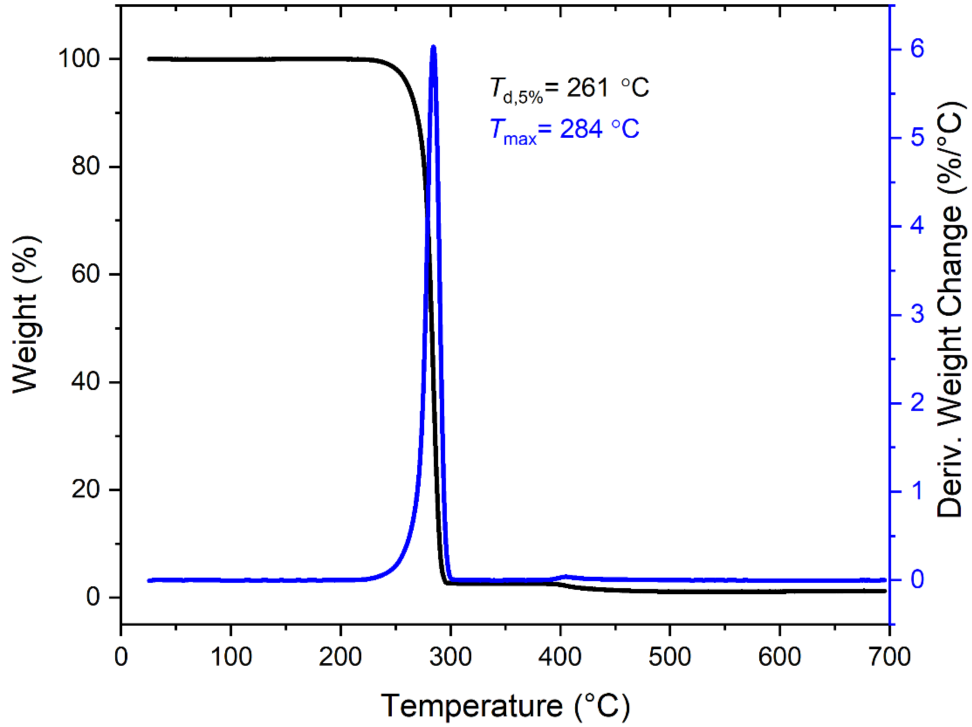

Figure S39. TGA and DTG curves of P3H2MP (M_n = 175 kDa, $Đ$ = 1.09, P_r = 0.88).

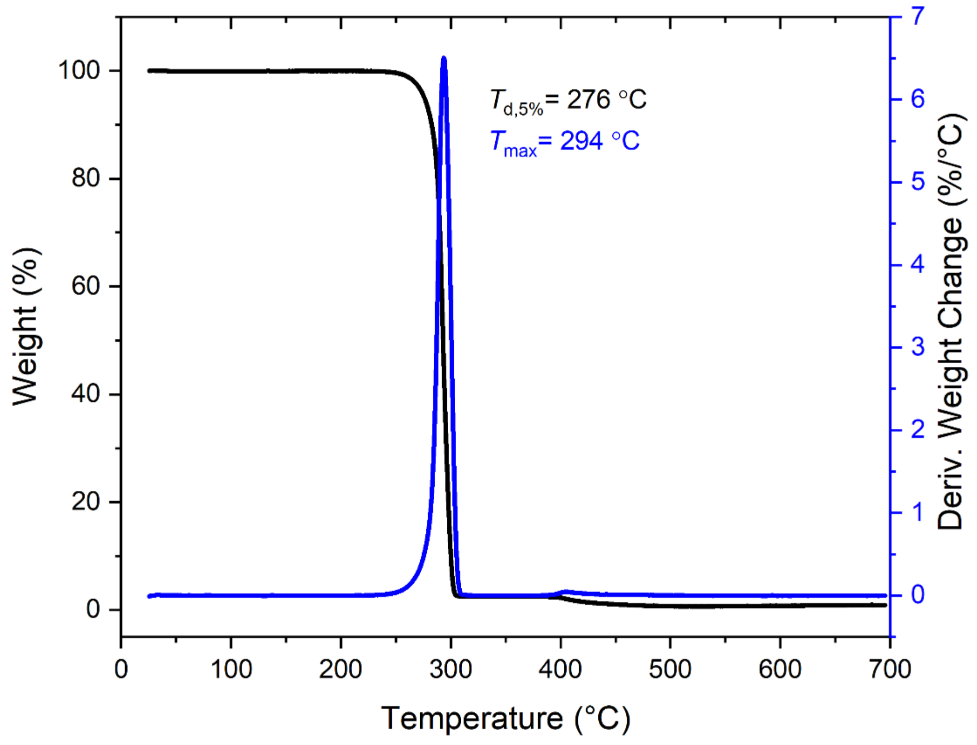

Figure S40. TGA and DTG curves of P3H2MP (M_n = 287 kDa, $Đ$ = 2.49, P_m = 0.72).

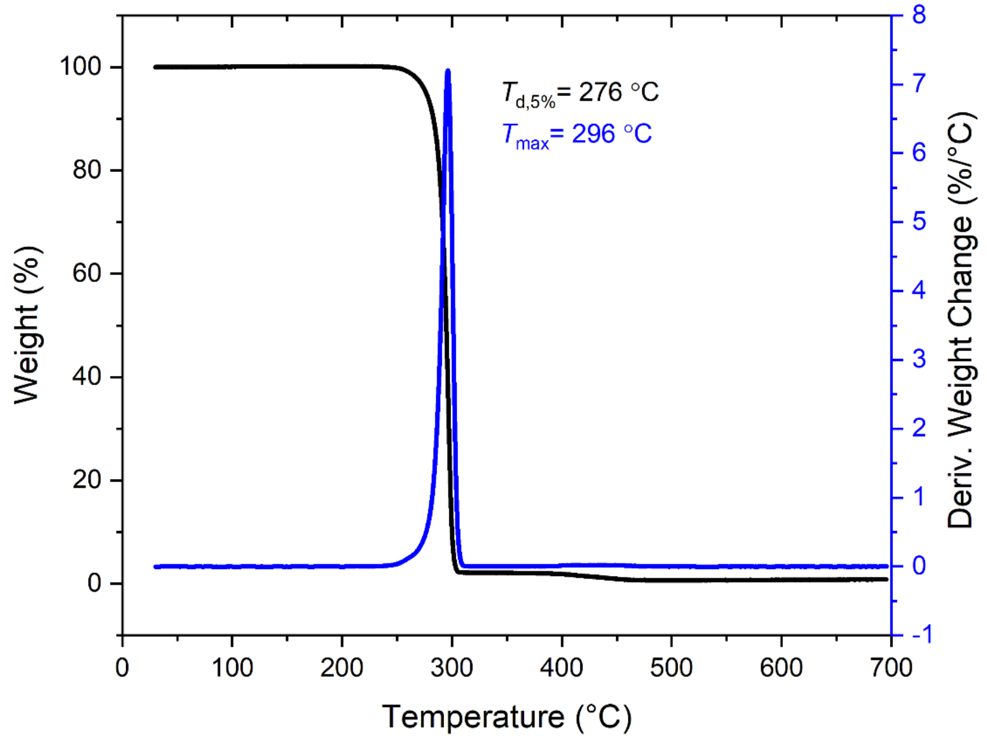

Figure S41. TGA and DTG curves of P3H2MP (M_n = 868 kDa, $Đ$ = 1.93, P_m = 0.80).

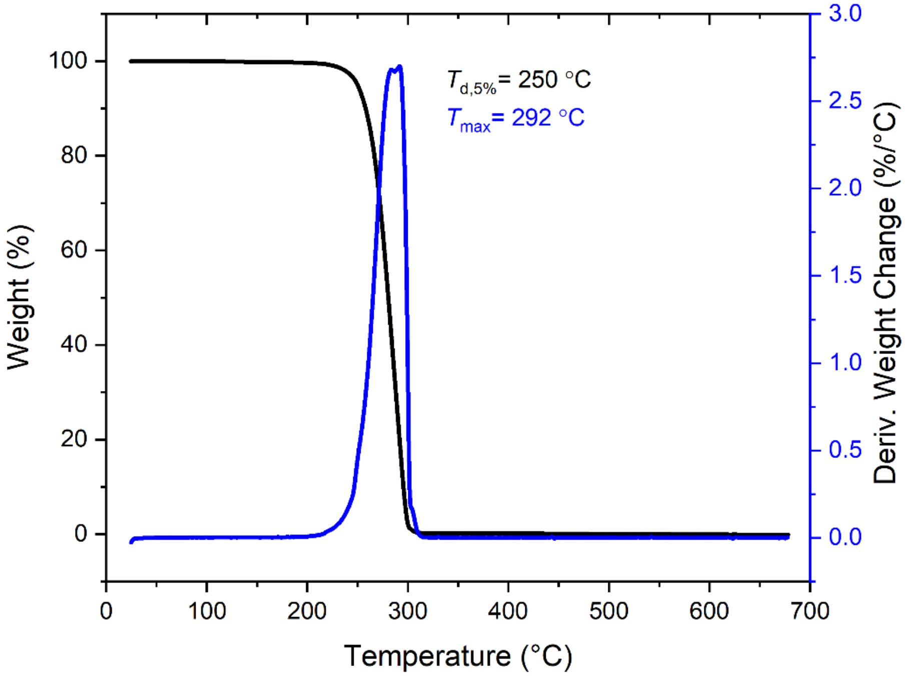

Figure S42. TGA and DTG curves of triblock copolymer *sr*-P3HB$_{50}$–*at*-P3H2MP$_{100}$–*sr*-P3HB$_{50}$ prepared with [*rac*-MPL]/[*rac*-BBL]/[**Y2**]/[BDM]= 100:100:1:0.5 (M_n = 23.8 kDa, $Đ$ = 1.30).

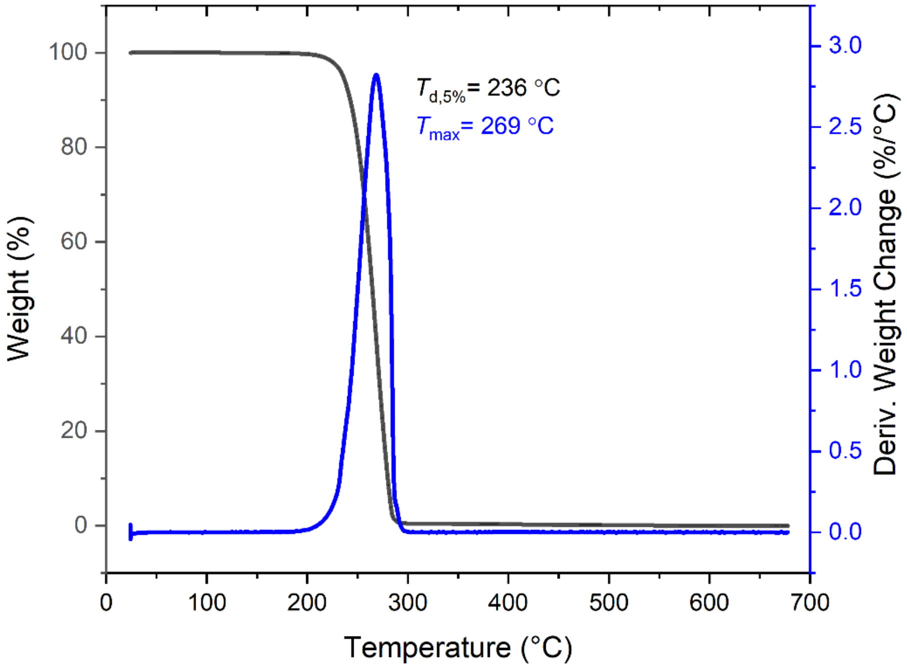

Figure S43. TGA and DTG curves of triblock copolymer *st*-P3HB$_{50}$–*sr*-P3H2MP$_{100}$–*st*-P3HB$_{50}$ prepared with [*rac*-MPL]/[*rac*-BBL]/[**Y3**]/[BDM]= 100:100:1:0.5 (M_n = 26.9 kDa, $Đ$ = 1.34).

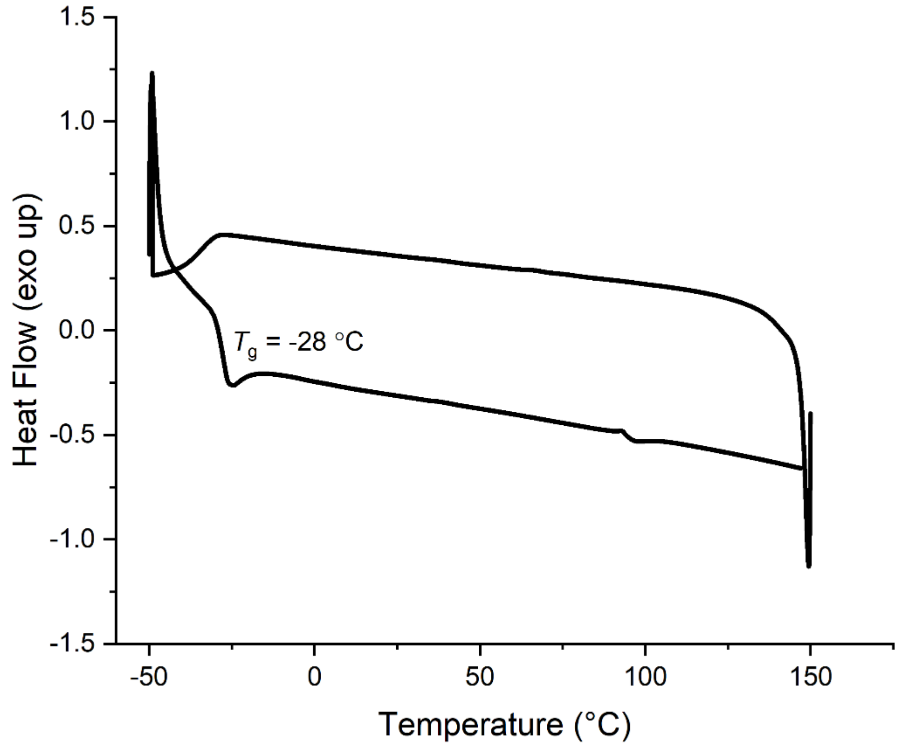

Figure S44. DSC curves of 1st cooling and 2nd heating scans of P3H2MP prepared with [MPL]/[La[N(SiMe$_3$)$_2$]$_3$]/[^{i}PrOH] = 1000:1:1 at 23 °C (M_n =37.5 kDa, $Đ$ =1.32, P_m = 0.52). Scan rate: 10 °C/min.

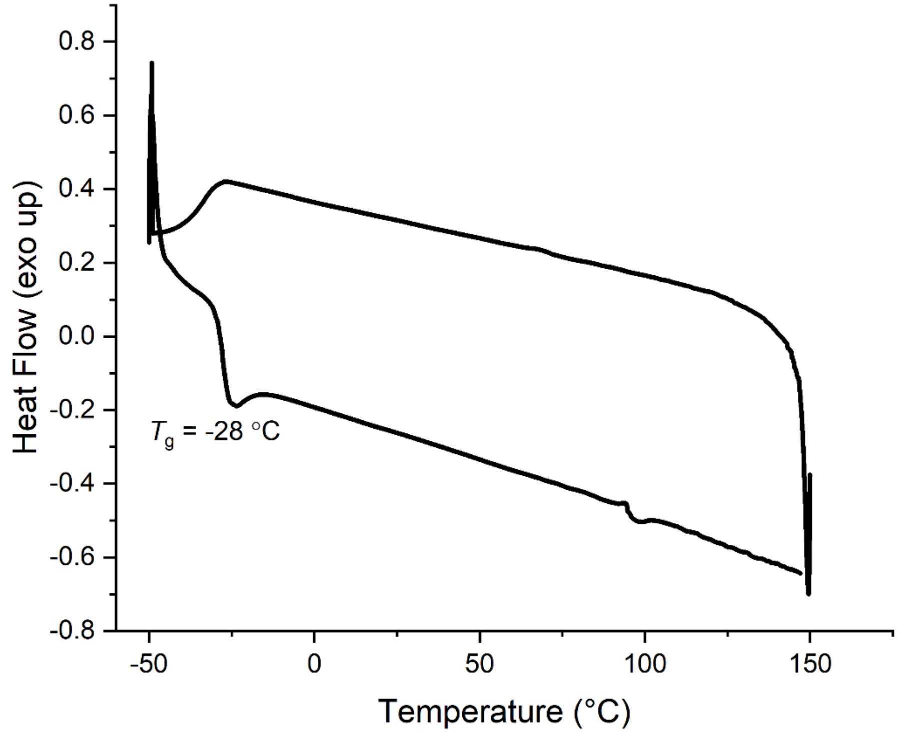

Figure S45. DSC curves of P3H2MP prepared with [MPL]/[**Y2**]/[^{i}PrOH] = 200:1:1 at 23 °C in toluene (M_n = 43.9 kDa, $Đ$ = 1.20, P_r = 0.61). Scan rate: 10 °C/min.

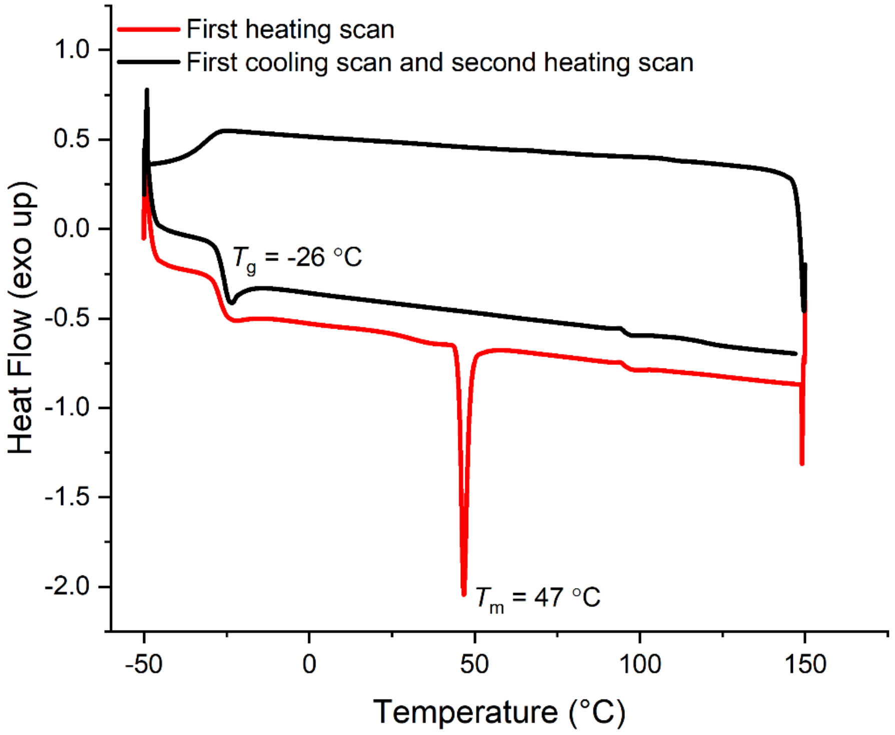

Figure S46. DSC curves of P3H2MP prepared with [MPL]/[**Y3**]/[^{i}PrOH] = 200:1:1 at 23 °C in DCM (M_n = 28.6 kDa, $Đ$ = 1.19, P_r = 0.64). Scan rate: 10 °C/min.

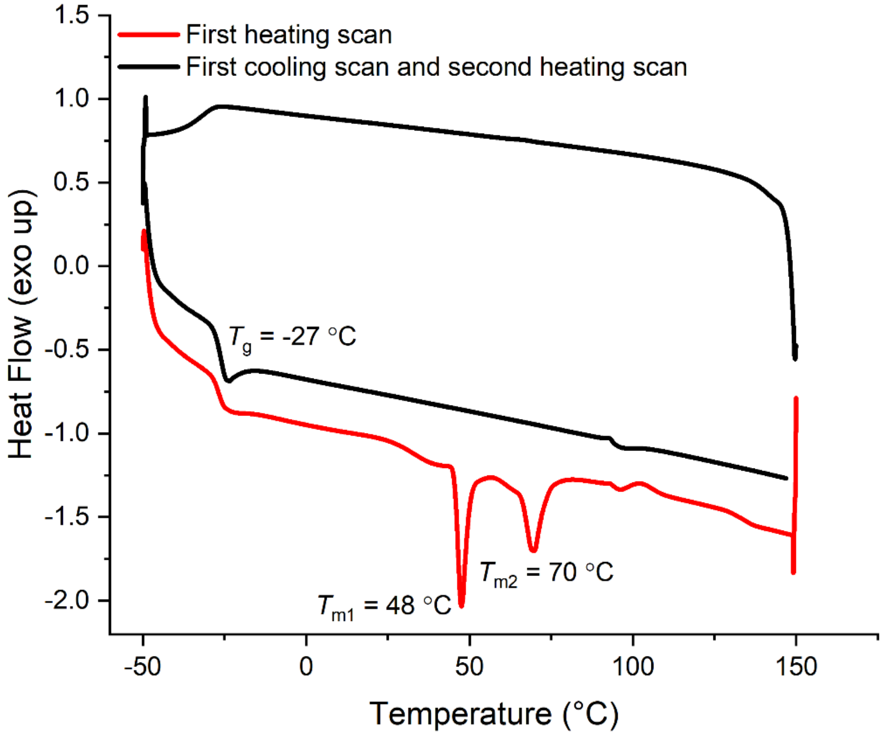

Figure S47. DSC curves of P3H2MP prepared with [MPL]/[**Y3**]/[^{i}PrOH] = 200:1:1 at 23 °C in toluene (M_n = 39.1 kDa, $Đ$ = 1.66, P_r = 0.72). Scan rate: 10 °C/min.

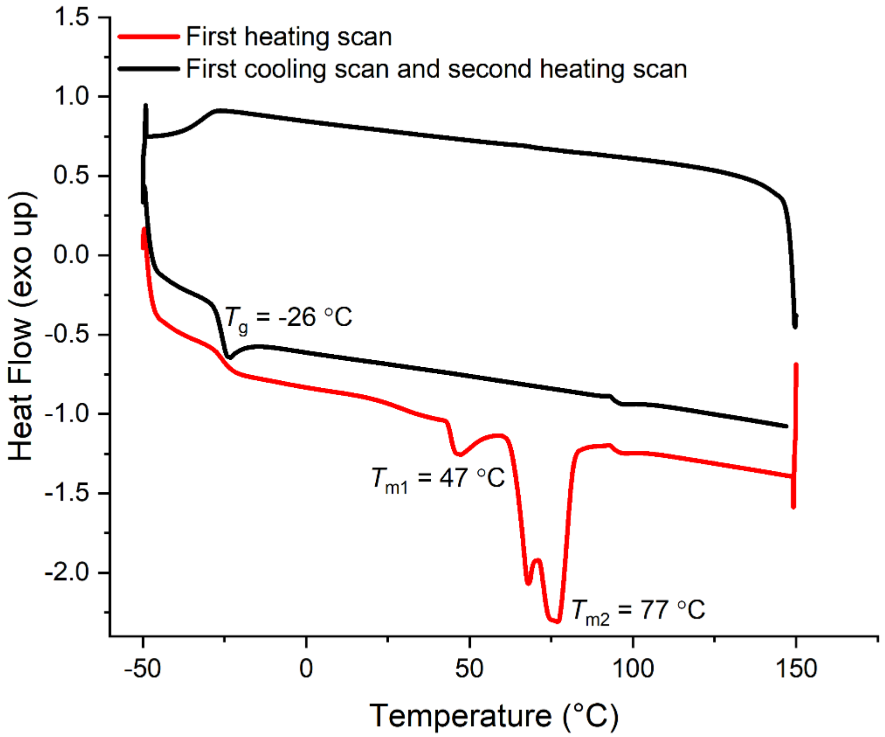

Figure S48. DSC curves of P3H2MP prepared with [MPL]/[**Y3**]/[^{i}PrOH] = 200:1:1 at -30 °C in toluene (M_n = 43.1 kDa, $Đ$ = 1.34, P_r = 0.76). Scan rate: 10 °C/min.

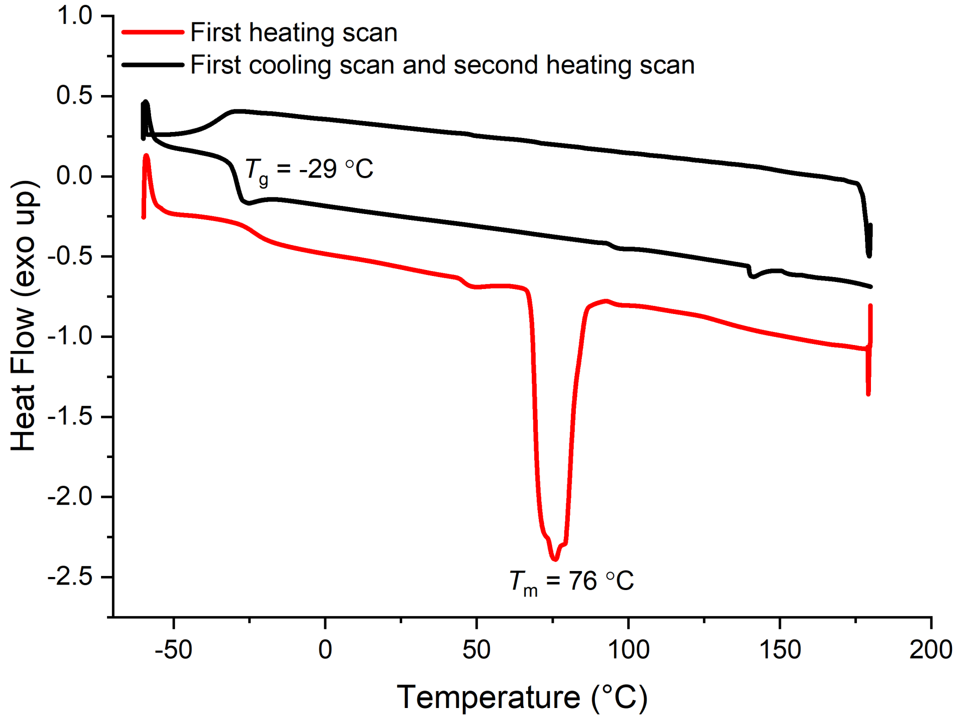

Figure S49. DSC curves of P3H2MP prepared with [MPL]/[**Y3**]/[^{i}PrOH] = 200:1:1 at -50 °C in toluene (M_n = 19.3 kDa, $Đ$ = 1.25, P_r = 0.80). Scan rate: 10 °C/min.

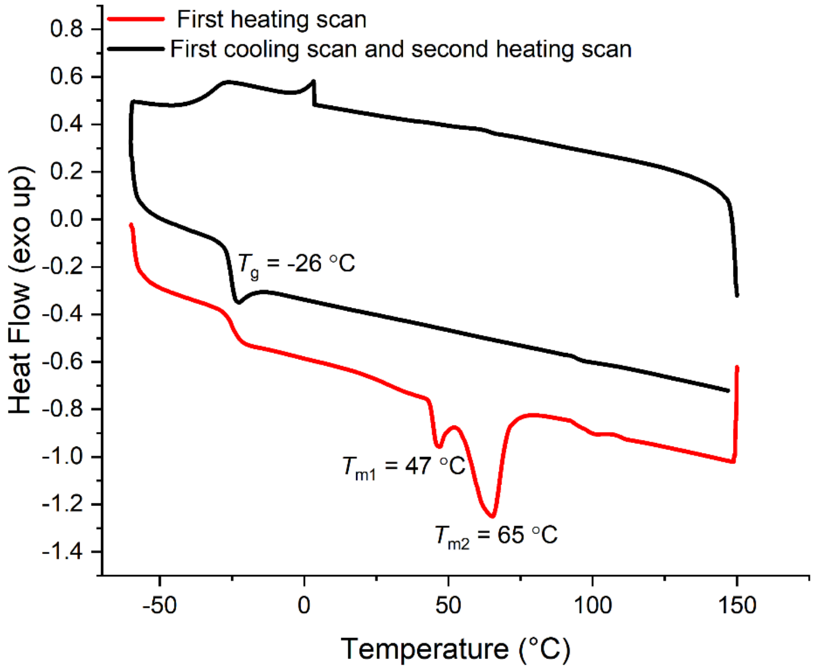

Figure S50. DSC curves of P3H2MP prepared with [MPL]/[**Y3**]/[^{i}PrOH] = 1600:0.7:1 at -50 °C in toluene (M_n = 189 kDa, $Đ$ = 1.07, P_r = 0.70). Scan rate: 10 °C/min.

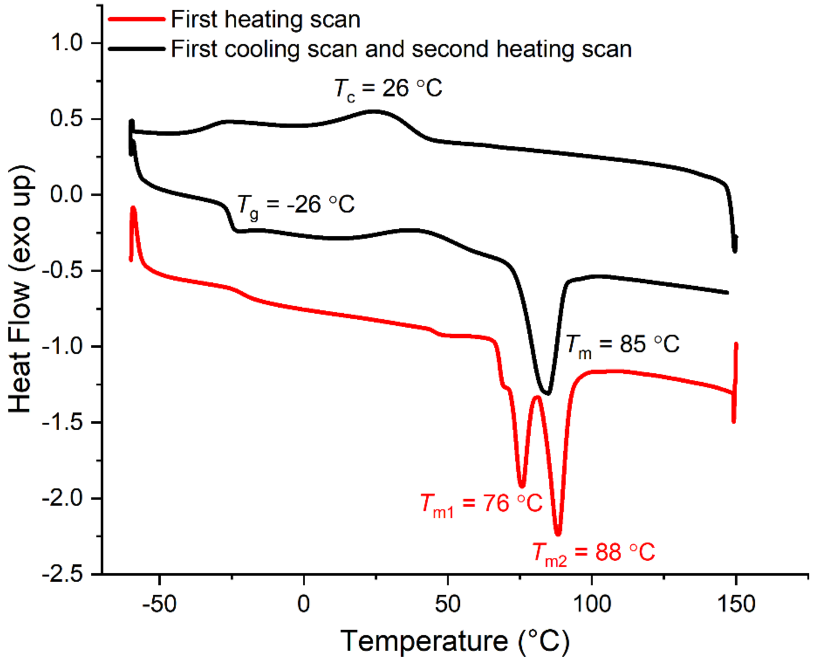

Figure S51. DSC curves of P3H2MP prepared with [MPL]/[**Y3**]/[^{i}PrOH] = 200:0.7:1 at -78 °C in toluene (M_n = 36.8 kDa, $Đ$ = 1.03, P_r = 0.87). Scan rate: 10 °C/min.

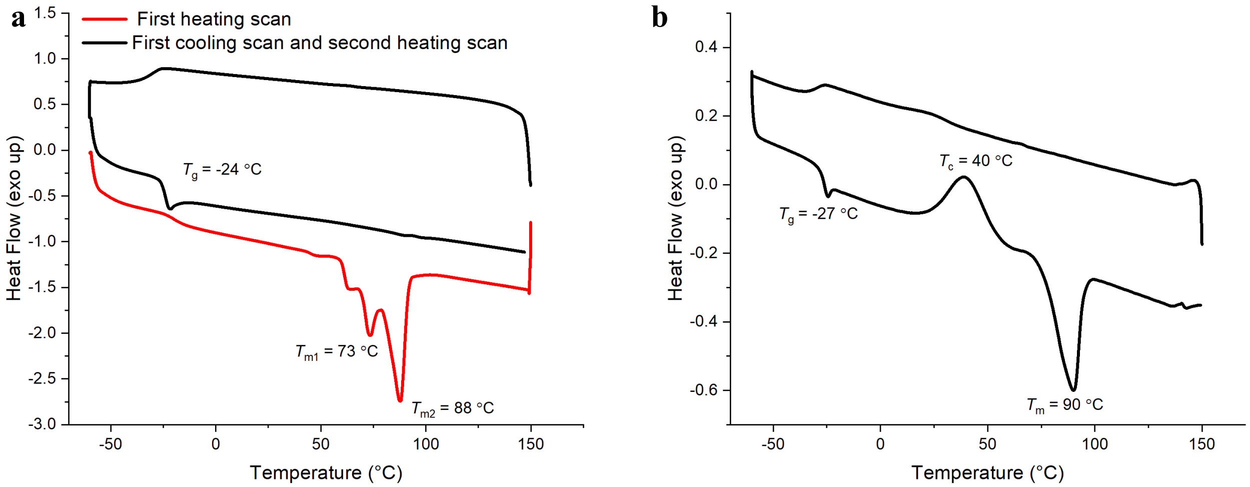

Figure S52. DSC curves of P3H2MP prepared with [MPL]/[**Y3**]/[^{i}PrOH] = 1600:0.7:1 at -78 °C in toluene. (M_n = 175 kDa, $Đ$ = 1.09, P_r = 0.88). **a**: Scan rate: 10 °C/min. **b**: Scan rate: 2 °C/min

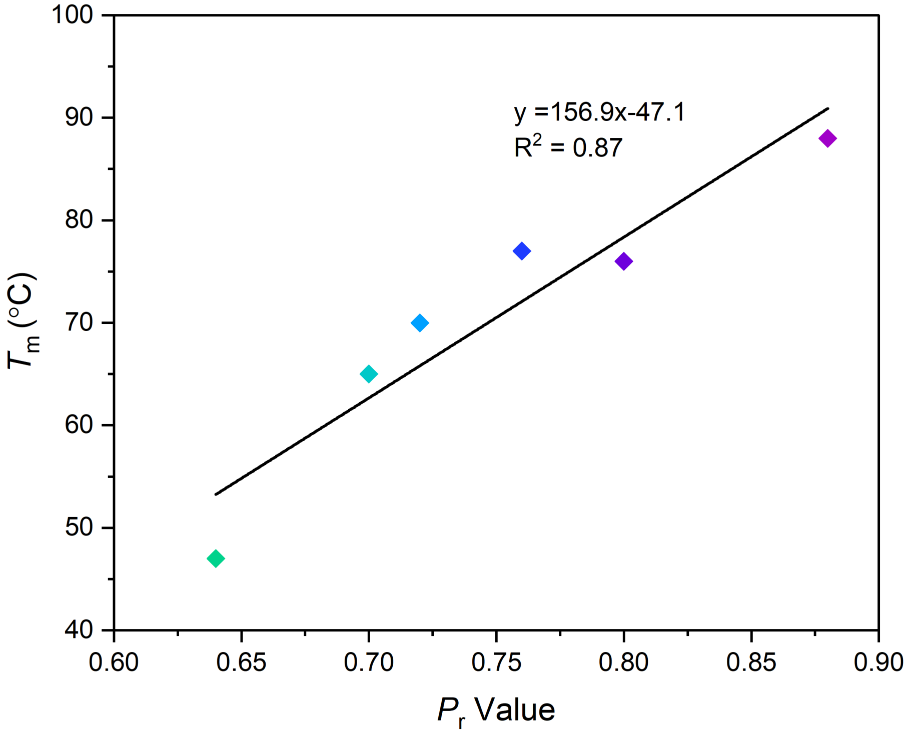

Figure S53. Plot of T_m *vs* P_r for P3H2MP. Linear fitting (R^2 = 0.87) reveals a strong positive correlation between tacticity and melting temperature. Extrapolation of the fitting suggests a theoretical T_m of 109.8 °C at P_r = 1.0.

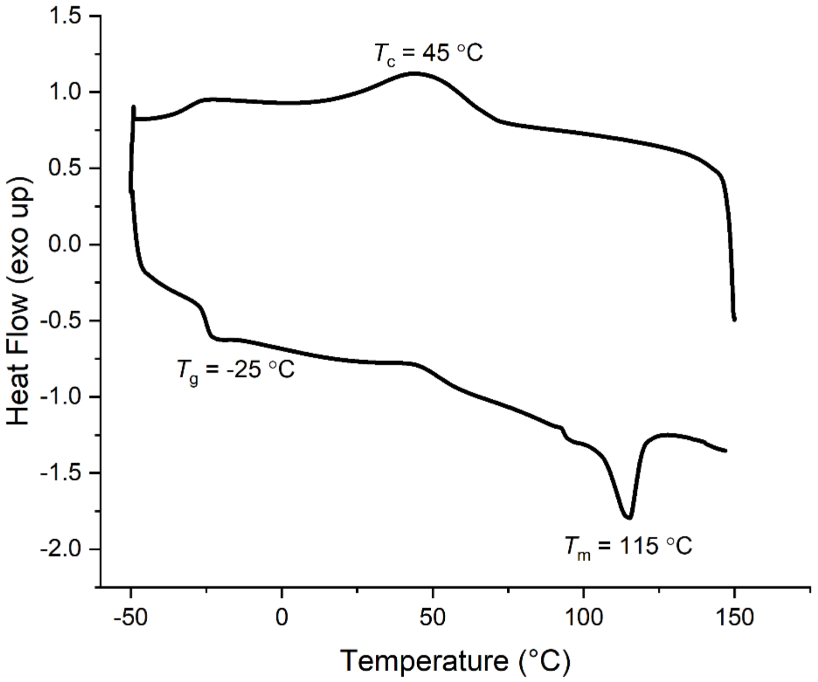

Figure S54. DSC curves of 1st cooling and 2nd heating scans of P3H2MP prepared with [MPL]/[**L6**+**Y6**] = 200:1 at 23 °C (Table S2, run 9, M_n = 51.3 kDa, $Đ$ = 2.48, P_m = 0.63). Scan rate: 10 °C/min.

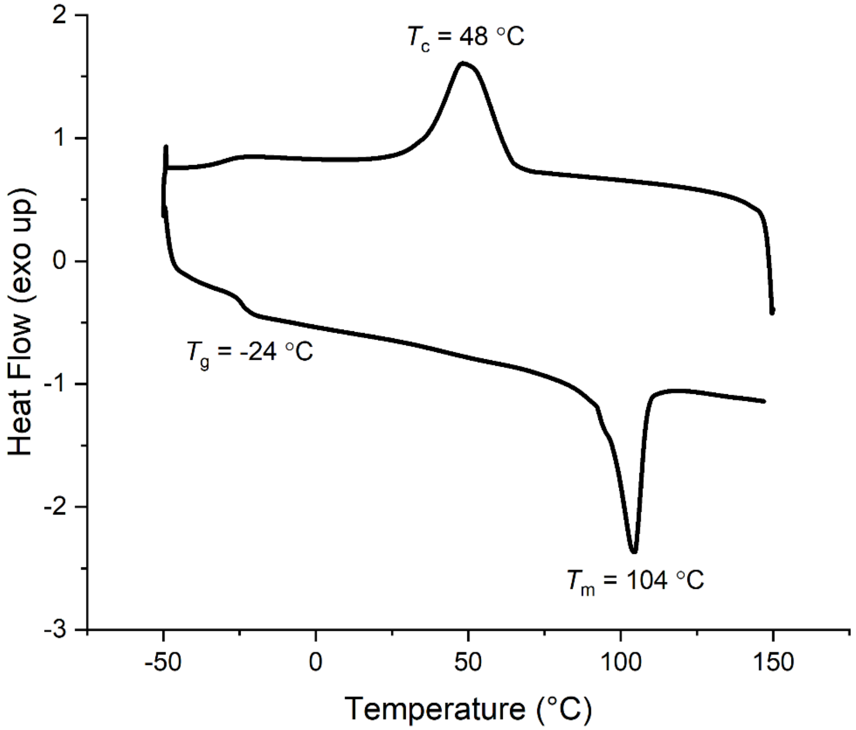

Figure S55. DSC curves of 1st cooling and 2nd heating scans of P3H2MP prepared with [MPL]/[**L6**+**La2**] = 500:1 at 23 °C (Table S2, run 23, M_n = 287 kDa, $Đ$ = 2.49, P_m = 0.72). Scan rate: 10 °C/min.

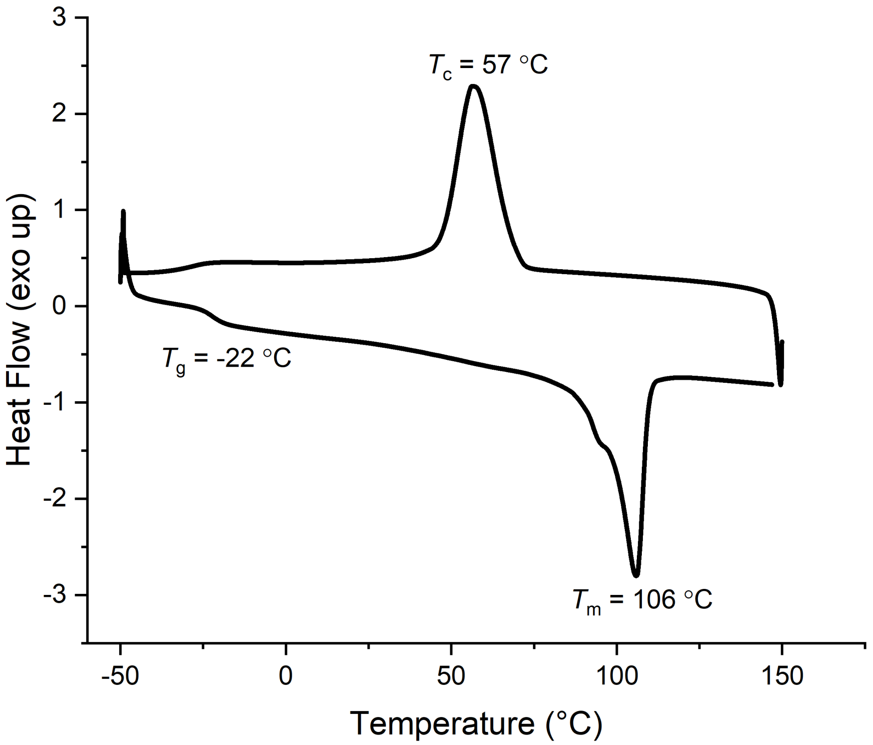

Figure S56. DSC curves of 1st cooling and 2nd heating scans of P3H2MP prepared with [MPL]/[**L6**+**La2**] = 500:1 at 23 °C (Table S2, run 22, M_n = 345 kDa, $Đ$ = 2.85, P_m = 0.75). Scan rate: 10 °C/min.

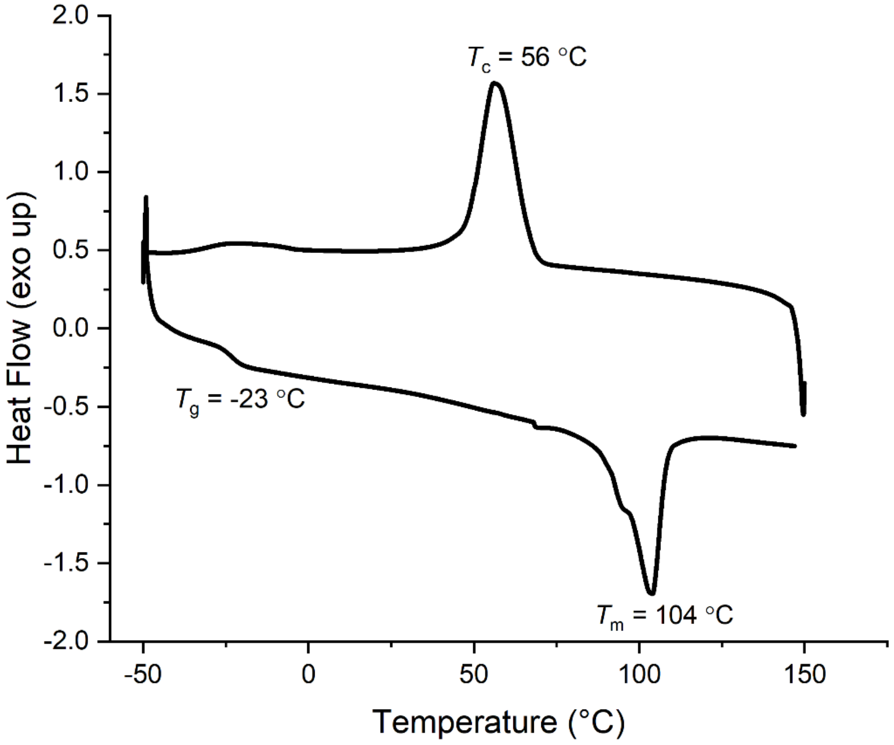

Figure S57. DSC curves of 1st cooling and 2nd heating scans of P3H2MP after fractionation (M_n = 419 kDa, $Đ$ = 1.79, P_m = 0.74). Scan rate: 10 °C/min.

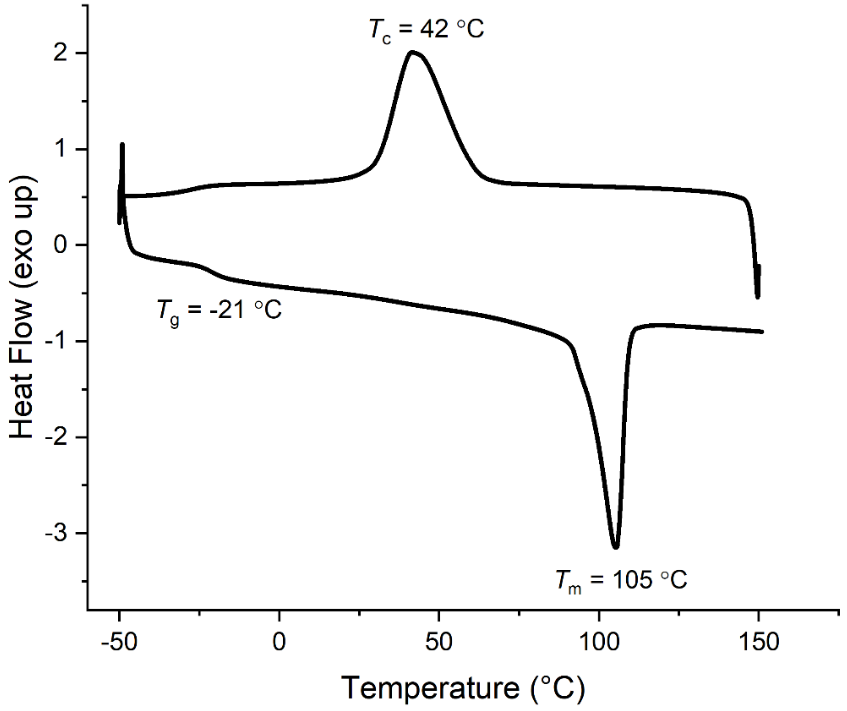

Figure S58. DSC curves of 1st cooling and 2nd heating scans of P3H2MP prepared with [MPL]/[**L6**+**Y6**] = 200:1 at 23 °C (Table S2, run 10, M_n = 868 kDa, $Đ$ = 1.93, P_m = 0.80). Scan rate: 10 °C/min.

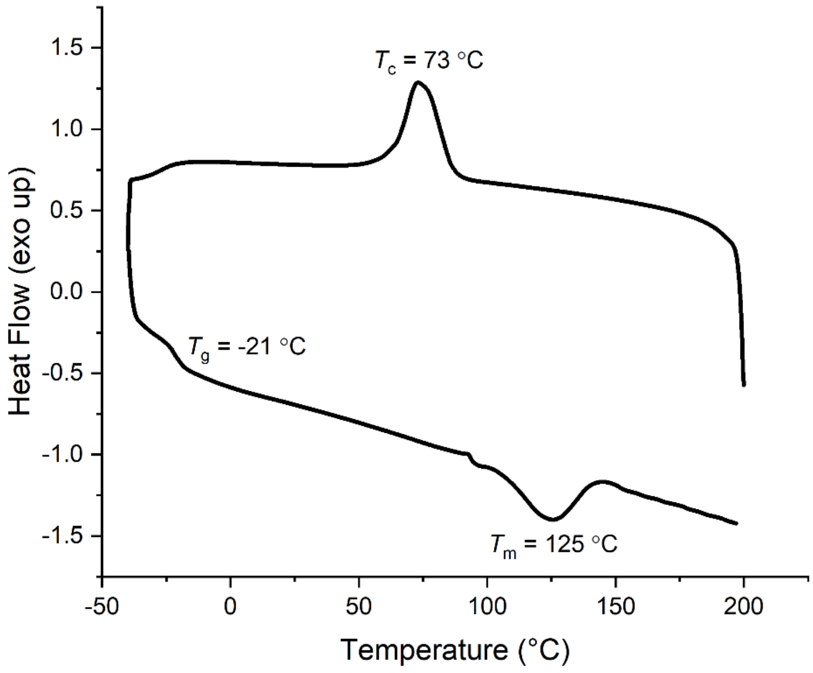

Figure S59. DSC curves of 1st cooling and 2nd heating scans of triblock copolymer *sr*-$P3HB_{50}$–*at*-$P3H2MP_{100}$–*sr*-$P3HB_{50}$ prepared with [*rac*-MPL]/[*rac*-BBL]/[**Y1**]/[BDM]= 100:100:1:0.5 at 23 °C (M_n = 21.8 kDa, $Đ$ = 1.08). Scan rate: 10 °C/min.

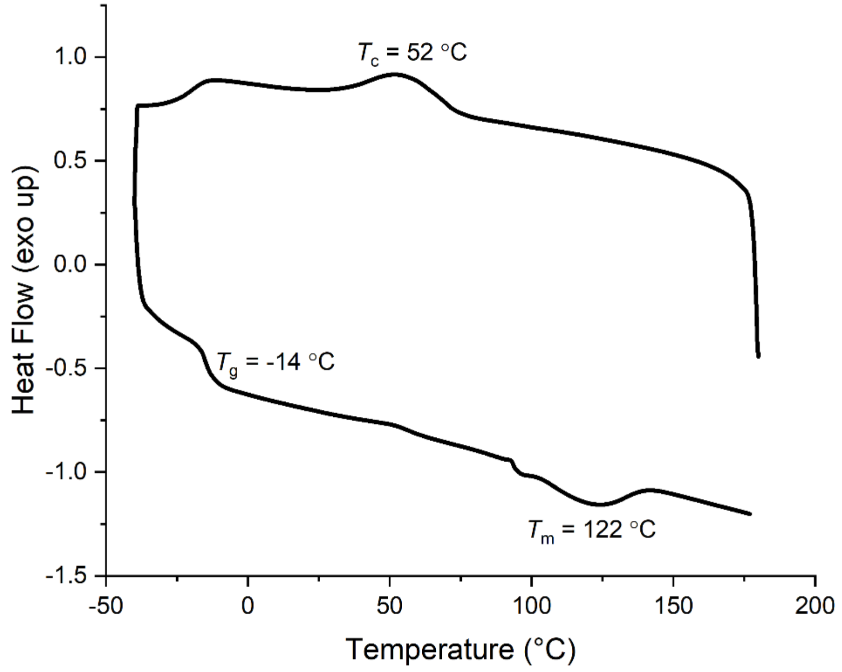

Figure S60. DSC curves of 1st cooling and 2nd heating scans of statistical copolymer P(3HB-*co*-3H2MP) prepared with [*rac*-MPL]/[*rac*-BBL]/[**Y2**]/[BDM]= 100:100:1:0.5 at 23 °C (M_n = 30.4 kDa, $Đ$ = 1.14). Scan rate: 10 °C/min.

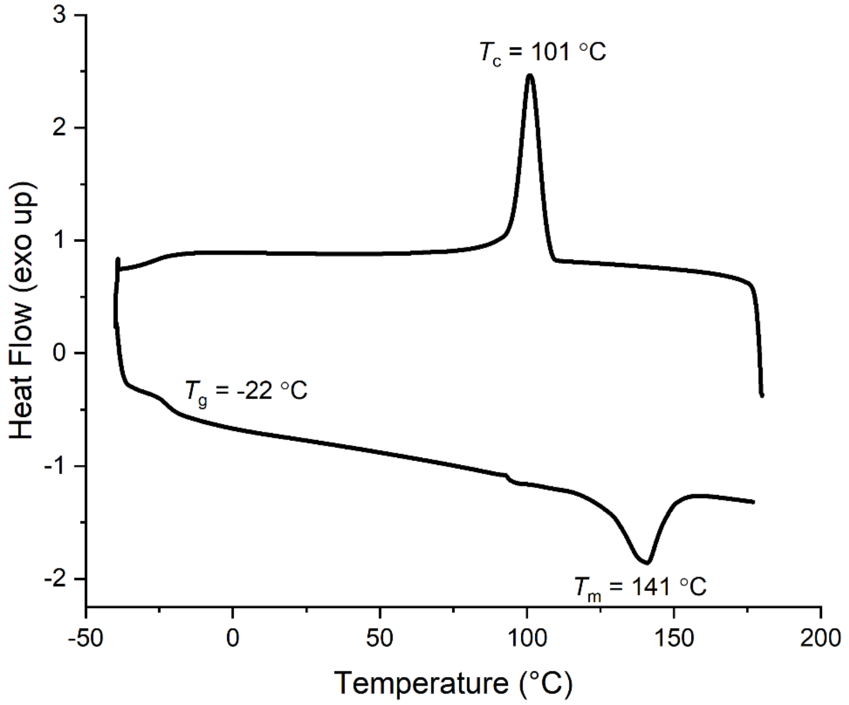

Figure S61. DSC curves of 1st cooling and 2nd heating scans of triblock copolymer *sr*-$P3HB_{50}$–*at*-$P3H2MP_{100}$–*sr*-$P3HB_{50}$ prepared with [*rac*-MPL]/[*rac*-BBL]/[**Y2**]/[BDM]= 100:100:1:0.5 at 23 °C (M_n = 23.8 kDa, $Đ$ = 1.30). Scan rate: 10 °C/min.

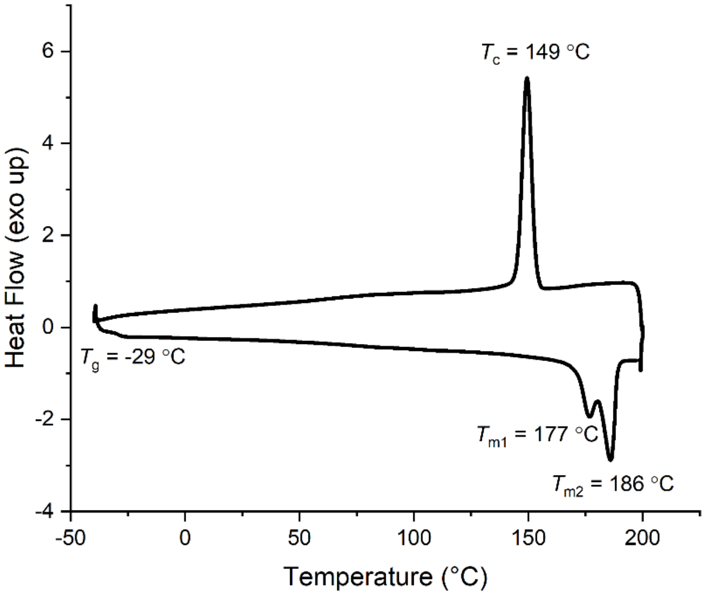

Figure S62. DSC curves of 1st cooling and 2nd heating scans of triblock copolymer *st*-P3HB$_{50}$–*sr*-P3H2MP$_{100}$–*st*-P3HB$_{50}$ prepared with [*rac*-MPL]/[*rac*-BBL]/[**Y3**]/[BDM]= 100:100:1:0.5 at 23 °C (M_n = 26.9 kDa, *Đ* = 1.34). Scan rate: 10 °C/min.

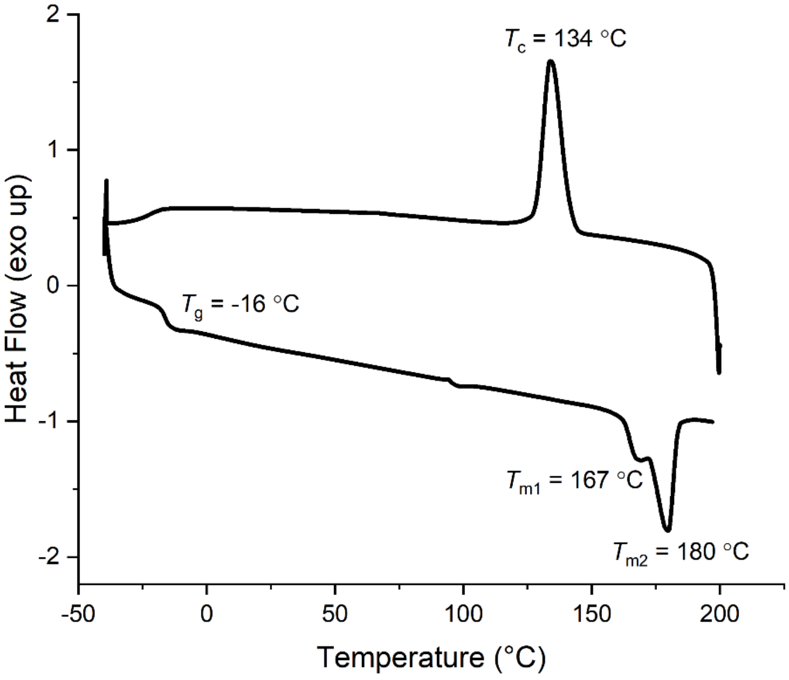

Figure S63. DSC curves of 1st cooling and 2nd heating scans of statistical copolymer P(*st*-3HB$_{50}$-*co*-3H2MP$_{50}$) prepared with [*rac*-MPL]/[*rac*-BBL]/[**L2**+**Y6**] = 100:100:1 at 23 °C (M_n = 56.4 kDa, *Đ* = 1.49). Scan rate: 10 °C/min.

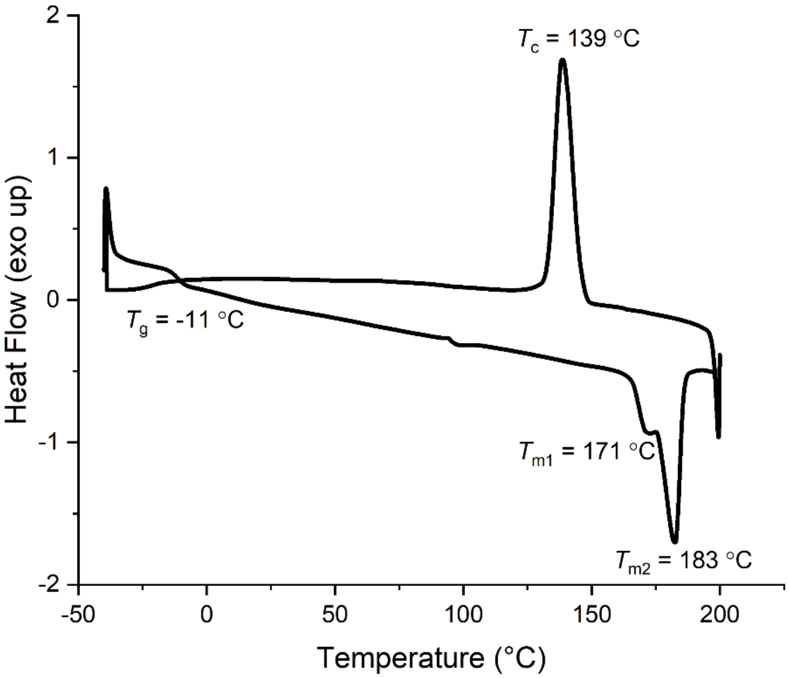

Figure S64. DSC curves of 1st cooling and 2nd heating scans of statistical copolymer P(*st*-3HB$_{71}$-*co*-3H2MP$_{29}$) prepared with [*rac*-MPL]/[*rac*-BBL]/[**L2**+**Y6**] = 60:140:1 at 23 °C (M_n = 77.1 kDa, $Đ$ = 1.52). Scan rate: 10 °C/min.

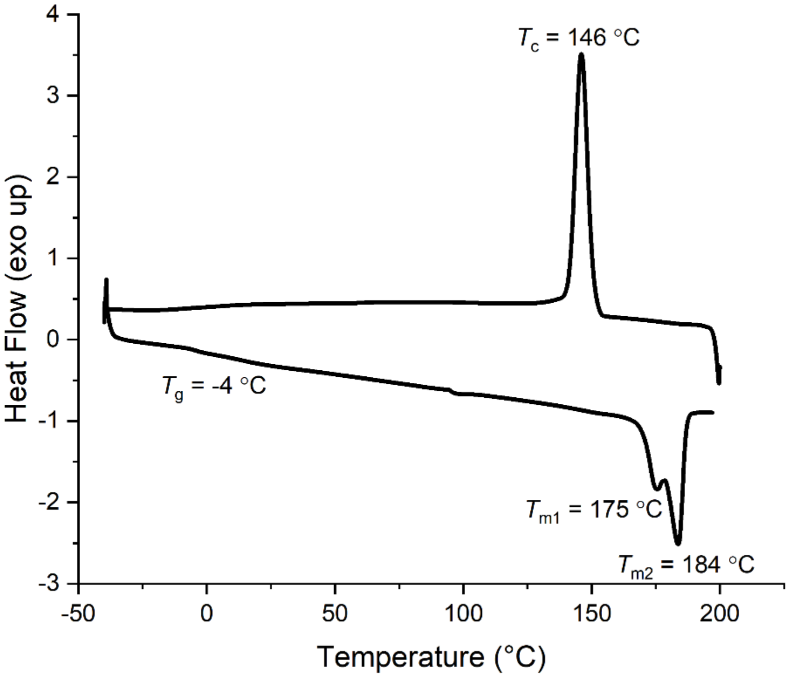

Figure S65. DSC curves of 1st cooling and 2nd heating scans of statistical copolymer P(*st*-3HB$_{89}$-*co*-3H2MP$_{11}$) prepared with [*rac*-MPL]/[*rac*-BBL]/[**L2**+Y6] = 20:180:1 at 23 °C (M_n = 76.7 kDa, $Đ$ = 1.84). Scan rate: 10 °C/min.

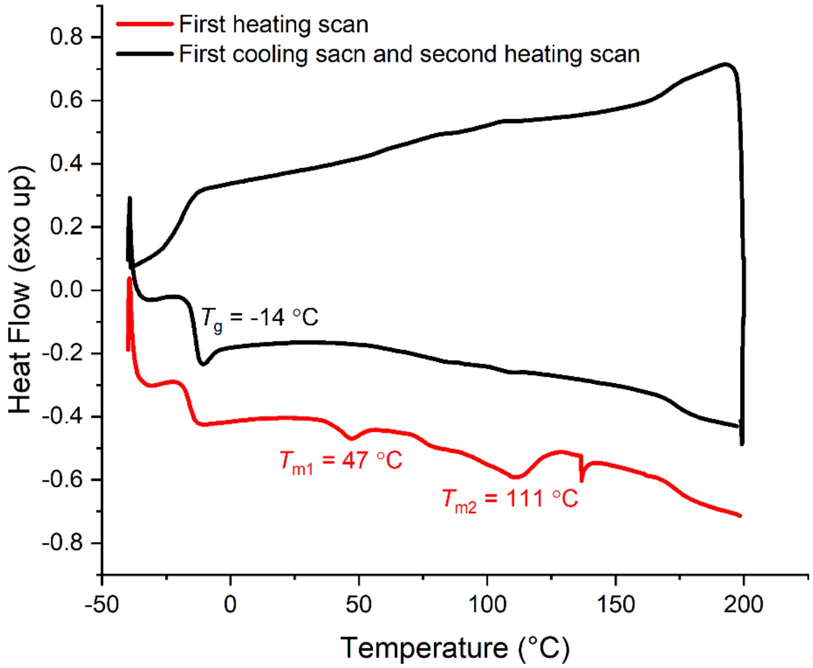

Figure S66. DSC curves of 1st cooling and 2nd heating scans of statistical copolymer P(*R*-3HB$_{47}$-*co*-3H2MP$_{53}$) prepared with [*rac*-MPL]/[(*R*)-BBL]/[**L1**+**Y6**] = 100:100:1 at 23 °C (M_n = 50.1 kDa, *Đ* = 1.25). Scan rate: 10 °C/min.

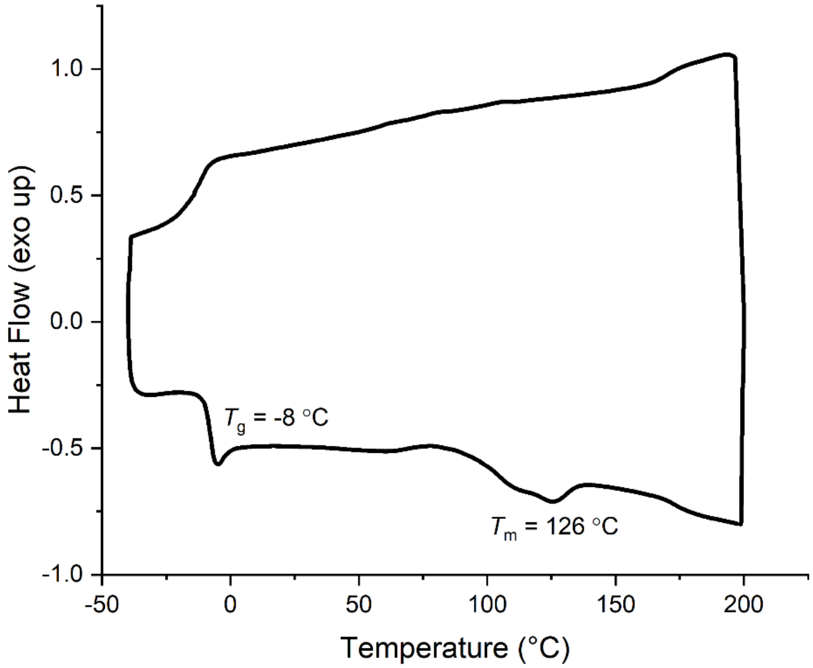

Figure S67. DSC curves of 1st cooling and 2nd heating scans of statistical copolymer P(*R*-3HB$_{68}$-*co*-3H2MP$_{32}$) prepared with [*rac*-MPL]/[(*R*)-BBL]/[**L1**+**Y6**] = 60:140:1 at 23 °C (M_n = 56.9 kDa, *Đ* = 1.29). Scan rate: 10 °C/min.

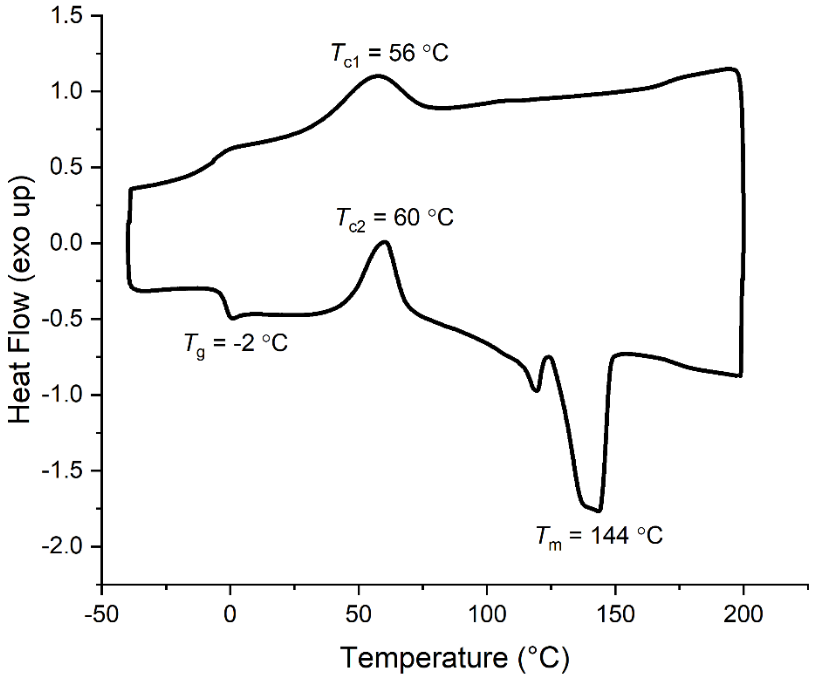

Figure S68. DSC curves of 1st cooling and 2nd heating scans of statistical copolymer P(R-3HB$_{88}$-co-3H2MP$_{12}$) prepared with [rac-MPL]/[(R)-BBL]/[**L1**+**Y6**] = 20:180:1 at 23 °C (M_n = 50.1 kDa, $Đ$ = 1.55). Scan rate: 10 °C/min.

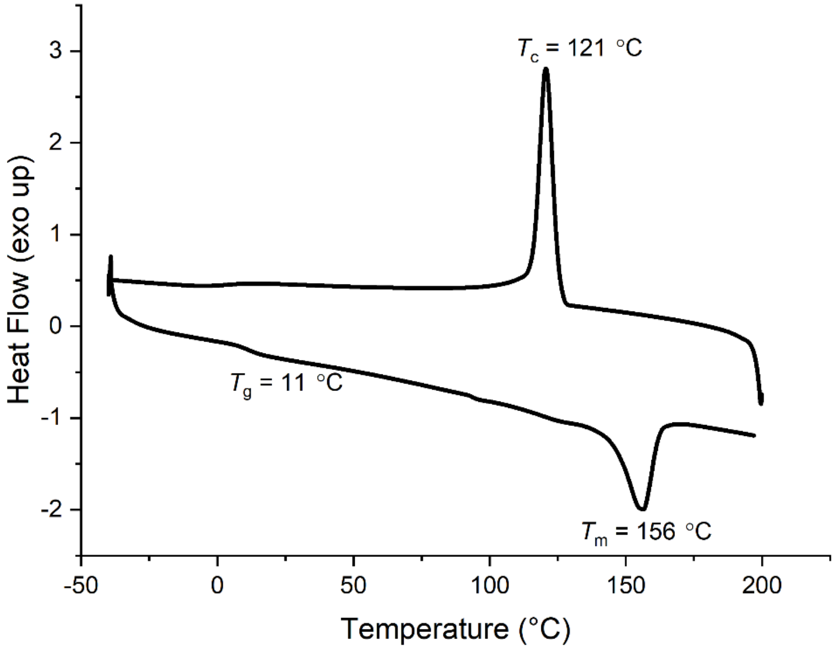

Figure S69. DSC curves of 1st cooling and 2nd heating scans of P3HB (M_n = 56.0 kDa, $Đ$ = 1.06, P_r = 0.84). Scan rate: 10 °C/min.

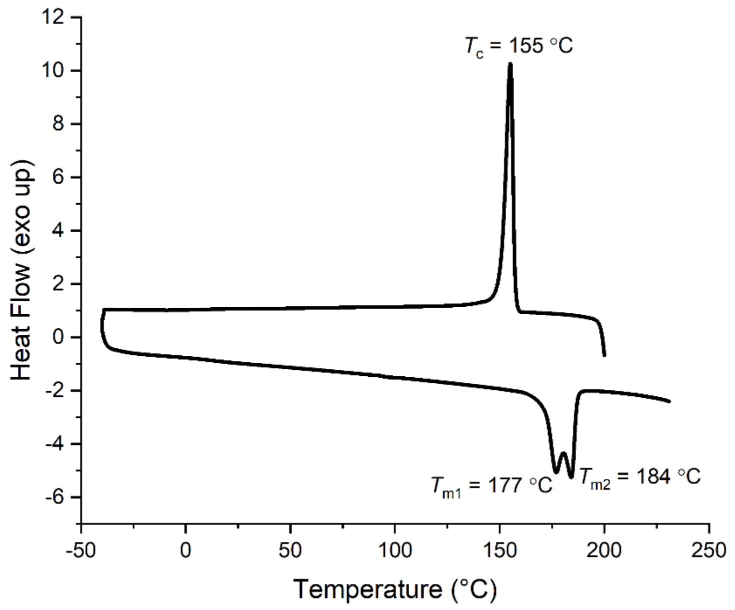

Figure S70. DSC curves of 1st cooling and 2nd heating scans of P3HB (M_n = 11.3 kDa, $Đ$ = 1.20, P_r = 0.91). Scan rate: 10 °C/min.

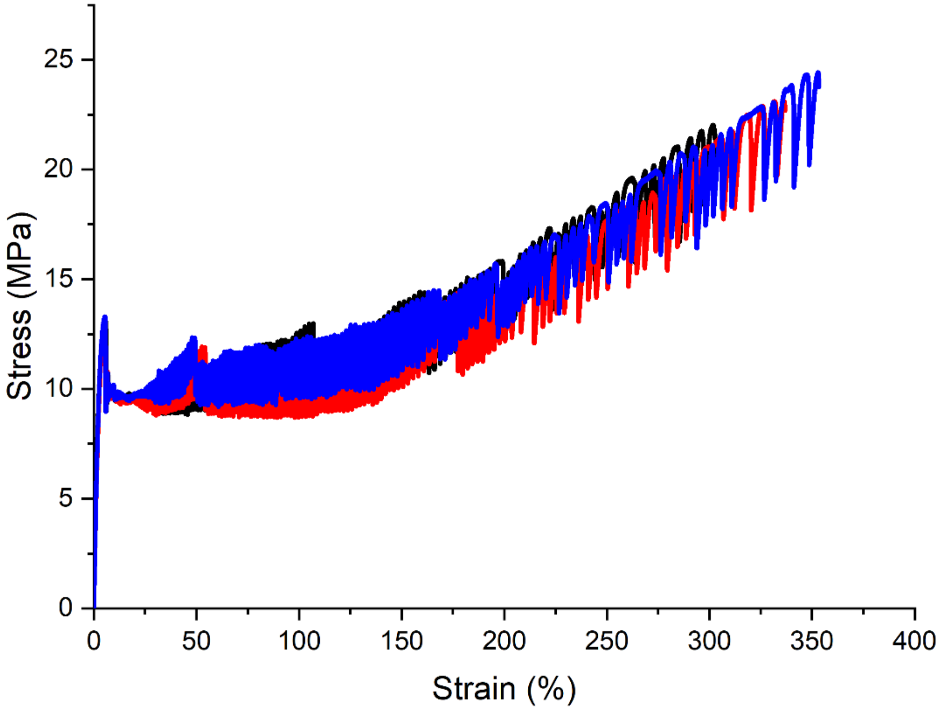

Figure S71. Tensile curves for *st*-P3H2MP (M_n = 175 kDa, $Đ$ = 1.09, P_r = 0.88), strain rate = 5 mm/min, ambient condition.

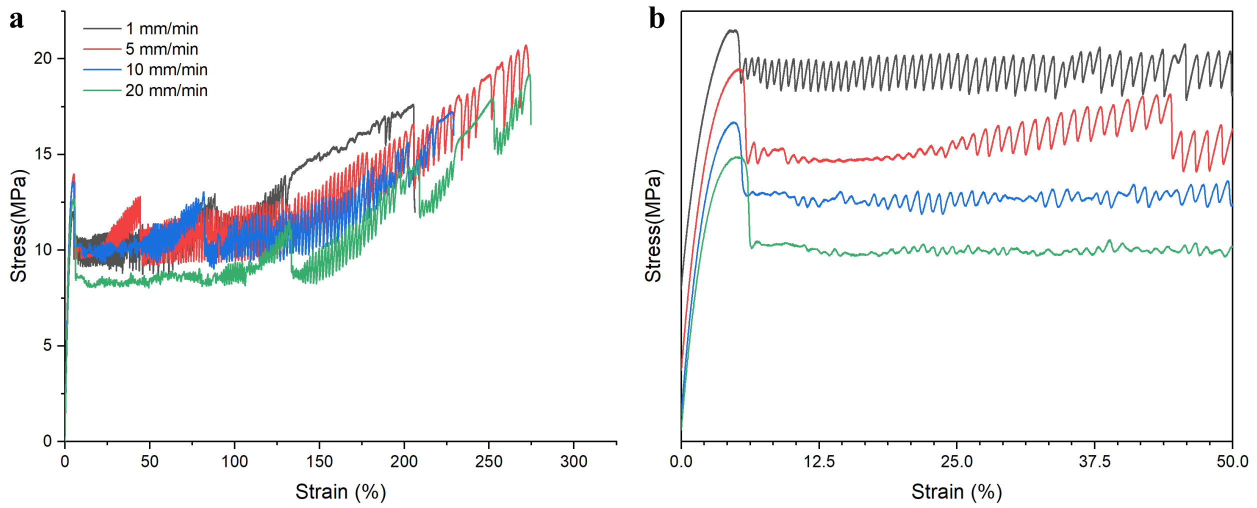

Figure S72. a: Tensile curves for *st*-P3H2MP (M_n = 175 kDa, $Đ$ = 1.09, P_r = 0.88) with different strain rates, ambient condition. **b**: Partial enlarged detail of tensile curves.

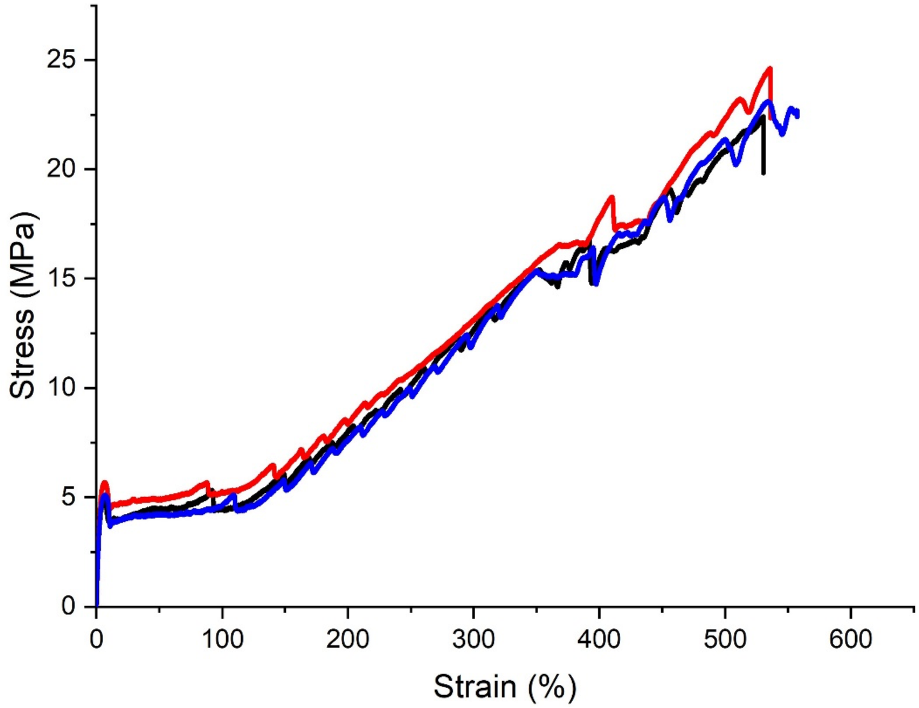

Figure S73. Tensile curves for *sr*-P3H2MP (M_n = 189 kDa, $Đ$ = 1.07, P_r = 0.70), strain rate = 5 mm/min, ambient condition.

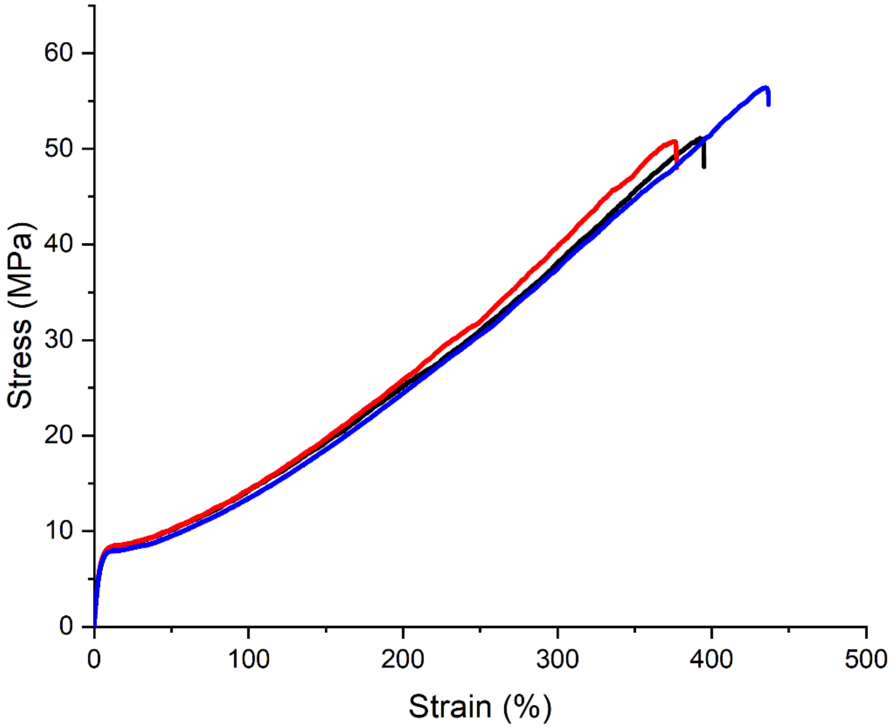

Figure S74. Tensile curves for *ir*-P3H2MP (M_n = 287 kDa, $Đ$ = 2.49, P_m = 0.72), strain rate = 5 mm/min, ambient condition.

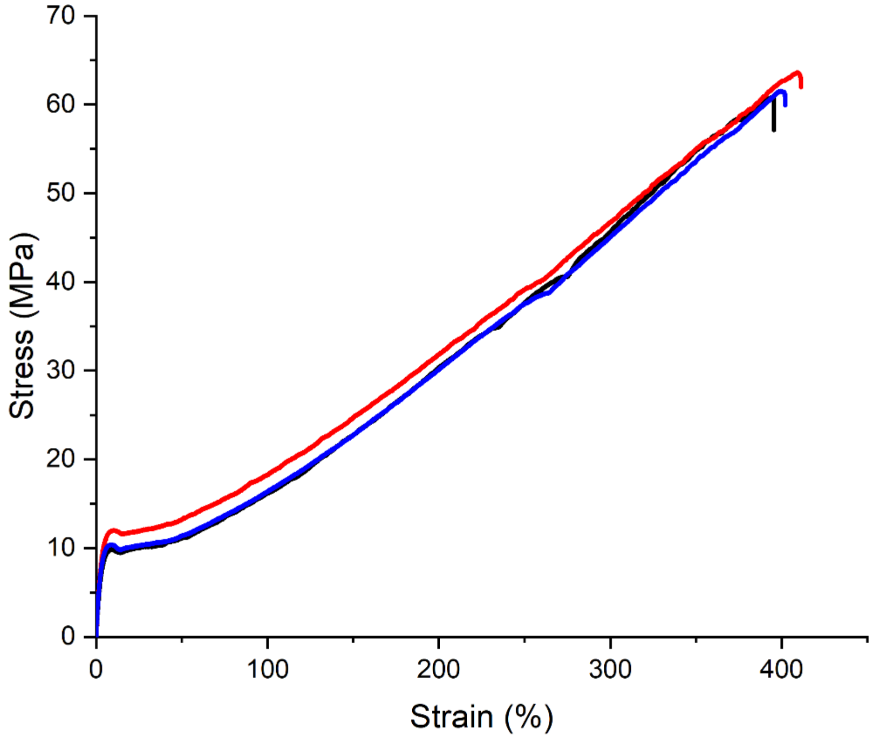

Figure S75. Tensile curves for *ir*-P3H2MP obtained after fractionation (M_n = 419 KDa, $Đ$ = 1.79, P_m = 0.74), strain rate = 5 mm/min, ambient condition.

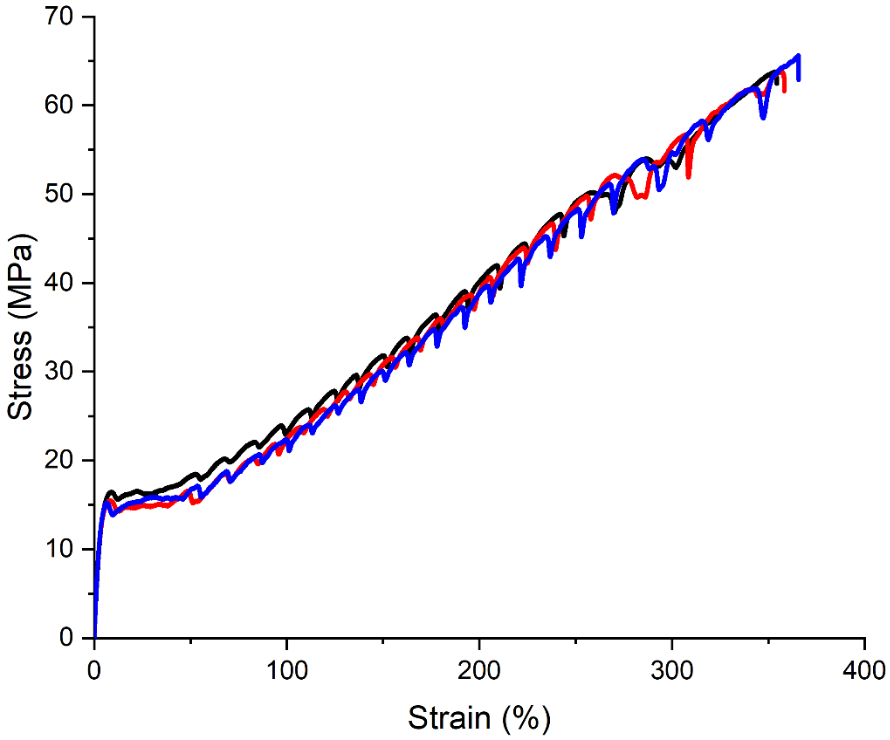

Figure S76. Tensile curves for *ir*-P3H2MP (M_n = 973 KDa, $Đ$ = 1.42, P_m = 0.80), strain rate = 5 mm/min, ambient condition.

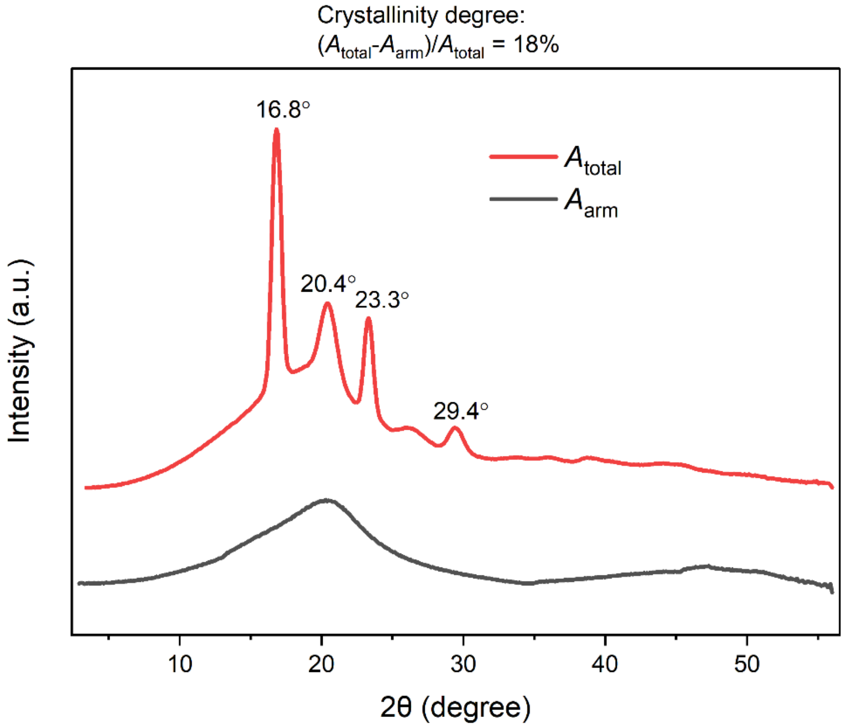

Figure S77. WAXS profiles of semicrystalline (P_r = 0.70) and amorphous P3H2MP.

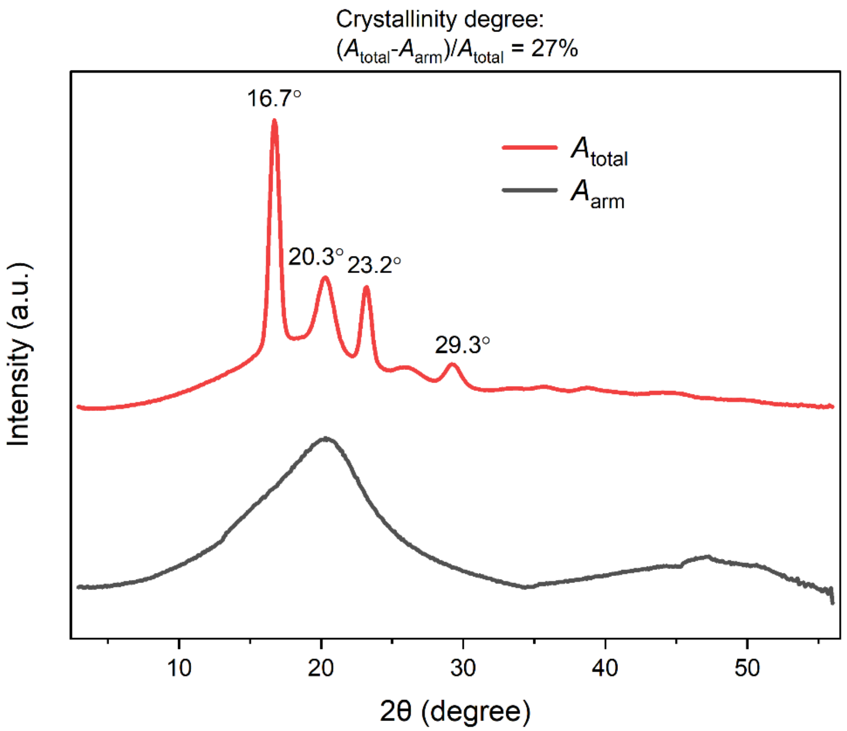

Figure S78. WAXS profiles of semicrystalline (P_r = 0.88) and amorphous P3H2MP.

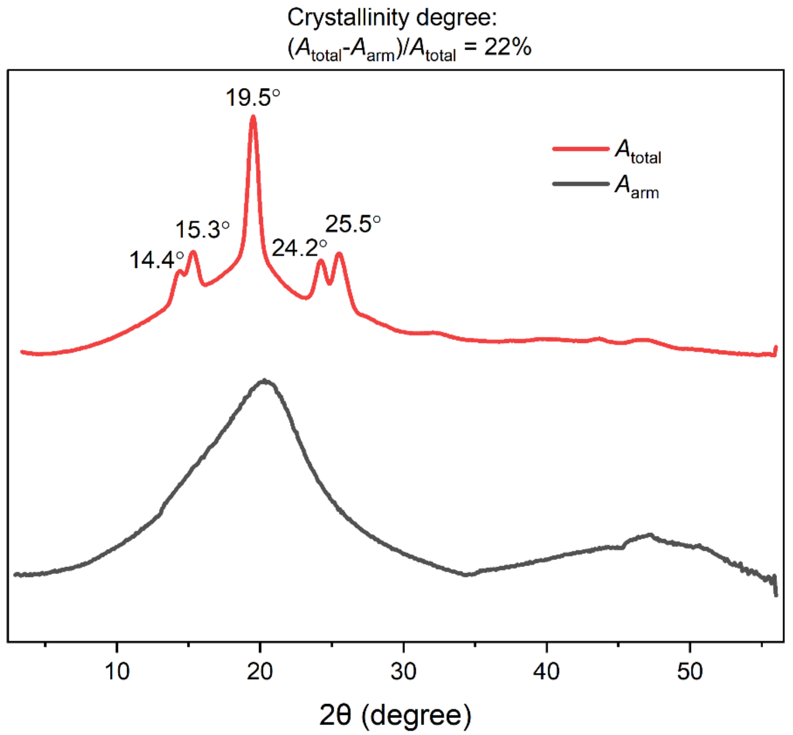

Figure S79. WAXS profiles of semicrystalline (P_m = 0.63) and amorphous P3H2MP.

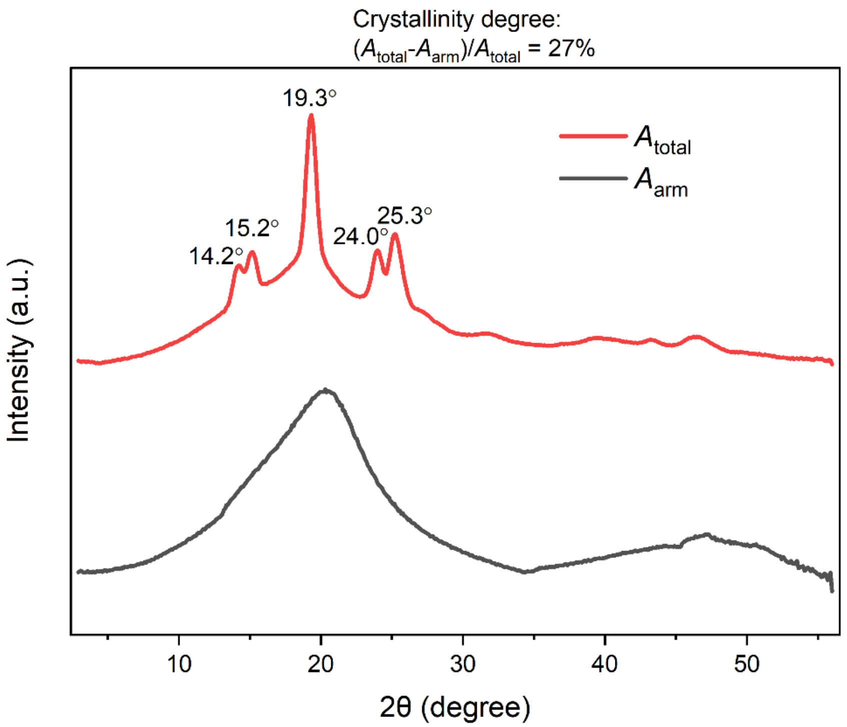

Figure S80. WAXS profiles of semicrystalline (P_m = 0.72) and amorphous P3H2MP.

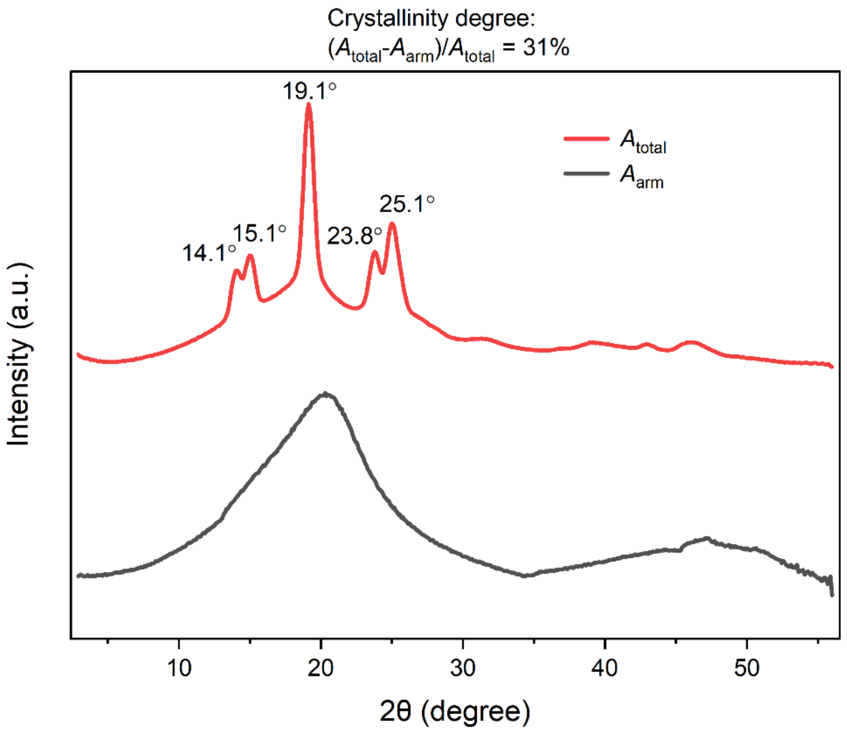

Figure S81. WAXS profiles of semicrystalline (P_m = 0.80) and amorphous P3H2MP.

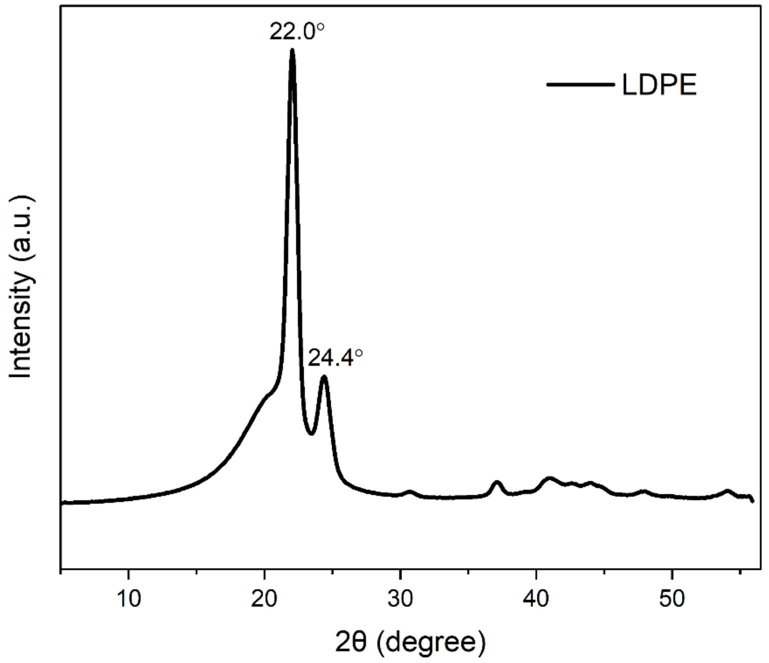

Figure S82. WAXS profile of LDPE (M_w = 80.5 kDa).

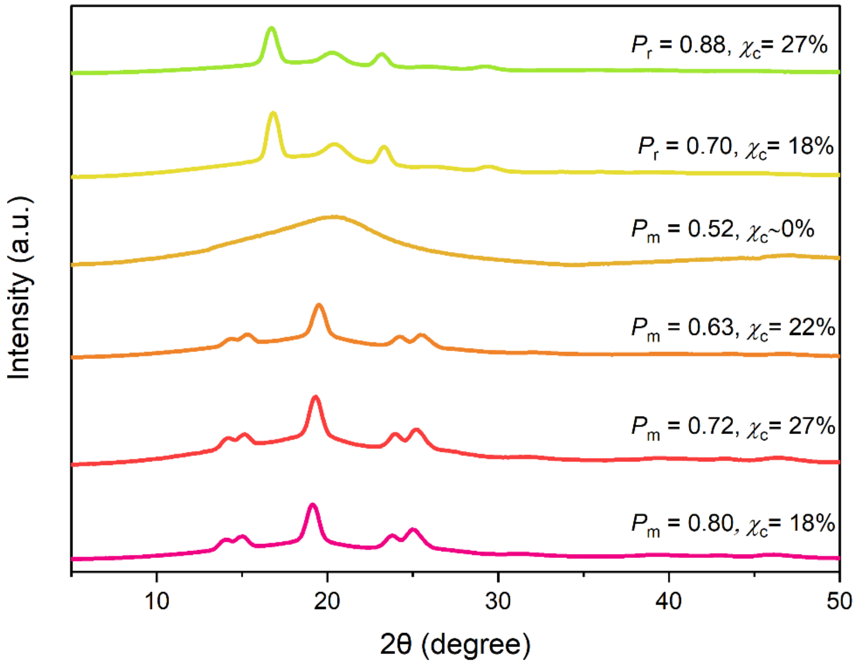

Figure S83. Overlayed WAXS profiles of *st*-P3H2MP (P_r = 0.70 and 0.88), atactic P3H2MP (P_m = 0.52), and *ir*-P3H2MP (P_m = 0.63, 0.72, and 0.80).

Supplementary Tables

Table S1. Syndioselective ring-opening polymerization of *rac*-MPL[a]

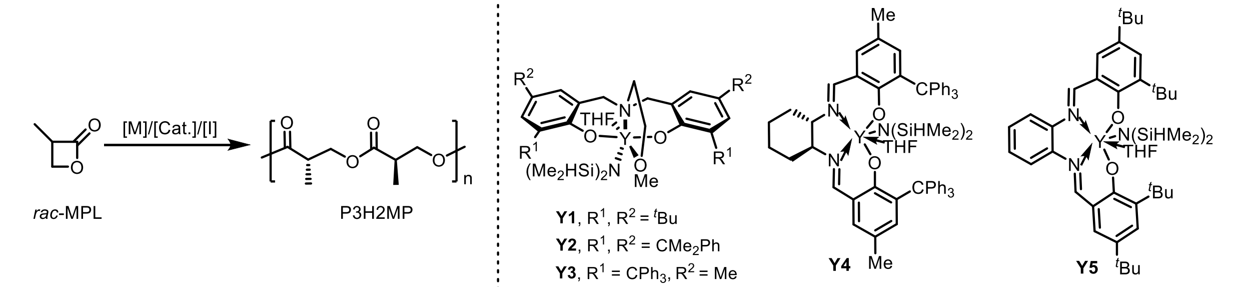

Runs	[M]/[Cat.]/[I]	[Cat.]	[I]	T/°C	Solvent	t/h	Conv. [%]	M_n[b] [kDa]	$Đ$[b]	P_r[c]
**1**	200:1:1	**Y1**	^{i}PrOH	23	Tol (1 M)	4	>99	22.0	1.16	0.53
**2**	200:1:1	**Y2**	^{i}PrOH	23	Tol (1 M)	4	>99	43.9	1.20	0.61
**3**	200:1:1	**Y3**	^{i}PrOH	23	Tol (1 M)	4	>99	39.1	1.66	0.72
**4**	200:1:1	**Y4**	^{i}PrOH	23	Tol (1 M)	4	>99	29.9	1.19	0.52
**5**	200:1:1	**Y5**	BnOH	23	DCM (2M)	4	93	16.8	1.55	0.48
**6**	200:1:1	**Y3**	^{i}PrOH	23	DCM (2M)	5	>99	28.6	1.19	0.64
**7**	200:1:1	**Y3**	^{i}PrOH	-30	Tol (1M)	4	>99	43.1	1.34	0.76
**8**	200:1:1	**Y3**	^{i}PrOH	-50	Tol (1M)	4	>99	19.3	1.25	0.80
**9**	200:1:1	**Y3**	^{i}PrOH	-78	DCM (1M)	5	>99	49.5	1.02	0.72
**10**	200:1:1	**Y3**	^{i}PrOH	-78	Tol (1 M)	5	>99	40.9	1.03	0.86
**11**	200:1:1	**Y3**	BnOH	-78	Tol (1 M)	3	>99	14.1	1.01	0.85
**12**	200:0.7:1	**Y3**	^{i}PrOH	-78	Tol (1 M)	5	>99	36.8	1.03	0.87
**13**	200:0.7:1	**Y3**	^{i}PrOH	-78	Tol (0.5 M)	5	>99	30.8	1.02	0.87
**14**[d]	400:0.7:1	**Y3**	^{i}PrOH	-78	Tol (1 M)	7	97	62.0	1.03	0.87
**15**[d]	800:0.7:1	**Y3**	^{i}PrOH	-78	Tol (1 M)	10	94	103	1.05	0.85
**16**[d]	1200:0.7:1	**Y3**	^{i}PrOH	-78	Tol (1 M)	24	53	154	1.03	0.88
**17**[d]	1600:0.7:1	**Y3**	^{i}PrOH	-78	Tol (1 M)	12	49	126	1.03	0.87
**18**[e]	1600:0.7:1	**Y3**	^{i}PrOH	-78	Tol (1 M)	28	51	175	1.09	0.88
**19**[f]	2000:0.7:1	**Y3**	^{i}PrOH	-78	neat	11	49	120	1.20	0.84
**20**[e]	1600:0.7:1	**Y3**	^{i}PrOH	-50	Tol (5 M)	23	>99	189	1.07	0.70

[a] Monomer conversion (Conv.) determined by ^{1}H NMR in $CDCl_3$. *rac*-MPL (1.0 mmol, 86.0 mg). [Cat.] = Catalyst. [I] = Initiator. [b] Weight-average (M_w) and number-average (M_n) molar mass and dispersity ($Đ = M_w/M_n$) determined via size exclusion chromatography (SEC) at 40 °C in $CHCl_3$ coupled with a Wyatt DAWN HELEOS II multi (18)-angle light scattering detector and a Wyatt Optilab TrEX dRI detector for absolute molar mass. [c] P_r is the probability of *racemic* linkages between **MPL** units. [d] *rac*-MPL (2.0 mmol, 172 mg). [e] *rac*-MPL (16.0 mmol, 1.38 g). [f] *rac*-MPL (10.0 mmol, 860 mg).

Table S2. Isoselective ring-opening polymerization of *rac*-MPL[a]

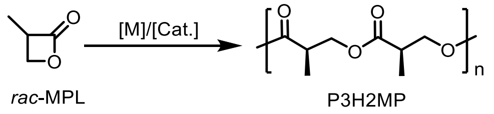

Runs	[M]/[Cat.]	[Cat.]	T/°C	Solvent	t/h	Conv. [%]	M_n[b] [kDa]	$Đ$[b]	P_m[c]
**1**	200:1	**L1**+**Y6**	23	Tol (1 M)	3	96	100	1.39	0.49
**2**	200:1	**L2**+**Y6**	23	Tol (2 M)	3	>99	87.8	1.07	0.38
**3**	200:1	**L2**+**Y6**	-30	Tol (2 M)	3	>99	171	1.54	0.27
**4**	200:1	**L2**+**Y6**	-78	Tol (2 M)	5	n.r.	-	-	-
**5**	200:1	**L2**+**Y6**	-78 to -30	Tol (2 M)	8	>99	68.4	2.18	0.24
**6**	200:1	**L3**+**Y6**	23	Tol (1 M)	3	>99	64.0	1.46	0.66
**7**	200:1	**L4**+**Y6**	23	Tol (1 M)	3	95	44.7	1.75	0.72
**8**	200:1	**L5**+**Y6**	23	Tol (1 M)	18	68	93.4	2.63	0.60
**9**	200:1	**L5**+**Y6**	23	DCM (1 M)	18	50	51.3	2.48	0.63
**10**	200:1	**L6**+**Y6**	23	Tol (1 M)	2	>99	868	1.93	0.80
**11**	200:1	**L6**+**Y6**	-30	Tol (2 M)	24	60	601	1.40	0.73
**12**[d]	500:1	**L6**+**Y6**	23	Tol (1 M)	5	>99	851	1.46	0.80
**13**[e]	500:1	**L6**+**Y6**	23	Tol (1 M)	16	91	973	1.42	0.80
**14**	200:1	**L7**+**Y6**	23	Tol (2 M)	17	91	623	1.11	0.56
**15**	200:1	**L8**+**Y6**	23	Tol (2 M)	20	95	107	1.93	0.62
**16**	200:1	**L9**+**Y6**	23	Tol (2 M)	24	65	59.6	1.49	0.58
**17**	200:1	**L10**+**Y6**	23	Tol (2 M)	20	>99	107	1.24	0.53
**18**	200:1	**L11**+**Y6**	23	Tol (2 M)	20	>99	107	1.89	0.52
**19**	200:1	**L5**+**La2**	23	Tol (2 M)	1.5	99	110	2.43	0.59
**20**	200:1	**L6**+**La2**	23	Tol (2 M)	4	>99	161	2.90	0.74
**21**	200:1	**L6**+**La1**	23	Tol (2 M)	12	>99	119	1.91	0.59
**22**[d]	500:1	**L6**+**La2**	23	Tol (2 M)	18	>99	345	2.85	0.75
**23**[e]	500:1	**L6**+**La2**	23	Tol (2 M)	18	>99	287	2.49	0.72
**24**	200:1	**L6**+**La2**	23	Tol (1 M)	4	>99	123	4.52	0.72
**25**	200:1	**L6**+**La2**	23	Tol (0.5 M)	4	>99	162	6.33	0.74
**26**	200:1	**L6**+**La2**	-30	Tol (2 M)	12	92	138	3.35	0.62
**27**	200:1	**L7**+**La2**	23	Tol (2 M)	1.5	99	106	1.80	0.52
**28**	200:1	**L6**+**Zn**	23	Tol (2 M)	12	76	90.1	1.35	0.62
**29**	200:1	**Y6**	23	Tol (1 M)	2	31	-	-	0.54
**30**	200:1	**La1**	23	Tol (1 M)	2	17	-	-	-

**31**	200:1	**La2**	23	Tol (1 M)	2	85	-	-	0.56

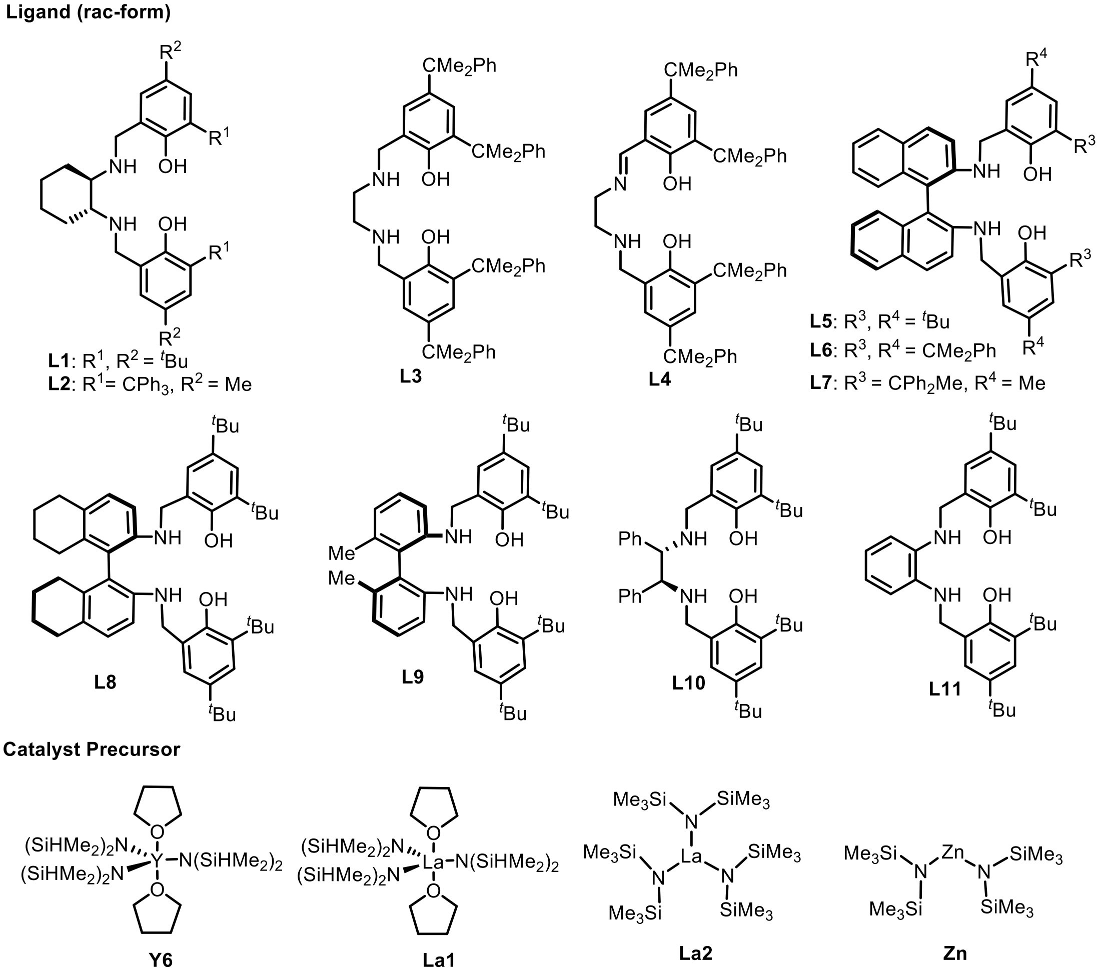

[a] Monomer conversion (Conv.) determined by ^{1}H NMR in $CDCl_3$. *rac*-MPL (1.0 mmol, 86.0 mg), [Cat.] = Catalyst. n.r. = no reaction. [b] Weight-average (M_w) and number-average (M_n) molar mass and dispersity ($Đ = M_w/M_n$) determined via size exclusion chromatography (SEC) at 40 °C in $CHCl_3$ coupled with a Wyatt DAWN HELEOS II multi (18)-angle light scattering detector and a Wyatt Optilab TrEX dRI detector for absolute molar mass. [c] P_m is the probability of *meso* linkages between **MPL** units. [d] *rac*-MPL (1.25 mmol, 107.5 mg). [e] *rac*-MPL (12.5 mmol, 1.07 g).

Table S3. Ring-opening polymerization of MPL with an initiator[a]

Runs	[M]/[Cat.]/[I]	[Cat.]	[I]	T/°C	Solvent	t/h	Conv. [%]	M_n[b] [kDa]	$Đ$[b]	P_m[c]
**1**	200:1:1	**L3**+**Y6**	^{i}PrOH	23	Tol (1 M)	12	>99	27.7	1.07	0.51
**2**	200:1:1	**L3**+**Y6**	BnOH	23	Tol (1 M)	12	>99	23.7	1.04	0.50
**3**	200:1:1	**L4**+**Y6**	BnOH	23	Tol (1 M)	12	>99	23.9	1.07	0.50
**4**	200:1:1	**L4**+**La2**	^{i}PrOH	23	Tol (2 M)	12	>99	41.4	1.14	0.48
**5**[d]	1000:1:1	**La2**	BnOH	23	Tol (2.5 M)	12	90	37.5	1.32	0.52

[a] Monomer conversion (Conv.) determined by ^{1}H NMR in $CDCl_3$. *rac*-MPL (1.0 mmol, 86.0 mg). [Cat.] = Catalyst. [I] = Initiator. [b] Weight-average (M_w) and number-average (M_n) molar mass and dispersity ($Đ$ = M_w/M_n) determined via size exclusion chromatography (SEC) at 40 °C in $CHCl_3$ coupled with a Wyatt DAWN HELEOS II multi (18)-angle light scattering detector and a Wyatt Optilab TrEX dRI detector for absolute molar mass. [c] P_m is the probability of *meso* linkages between MPL units. [d] *rac*-MPL (2.5 mmol, 215 mg)

Table S4. Ring-opening polymerization of *rac*-MPL with aluminum complexes [a]

Runs	[M]/[Cat.]	[Cat.]	T/°C	Solvent	t/h	Conv. [%]	M_n [kDa][b]	$Đ$[b]	P_m[c]
**1**	200:1	**Al-1**	23	Tol (2 M)	12	n.r	-	-	
**2**	200:1	**Al-1**	60	Tol (2 M)	12	>99	10.0	1.03	0.54
**3**	200:1	**Al-2**	23	Tol (2 M)	24	n.r.	-	-	-
**4**	200:1	**Al-2**	50	Tol (2 M)	24	<5	-	-	-

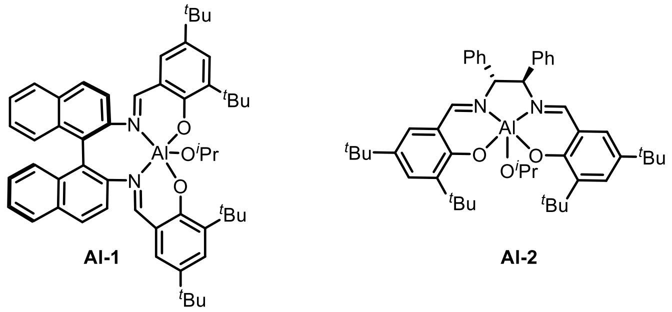

[a] Monomer conversion (Conv.) determined by ^{1}H NMR in $CDCl_3$. *rac*-MPL (1.0 mmol, 86.0 mg), [Cat.] = Catalyst. n.r.= no reaction. [b] Weight-average (M_w) and number-average (M_n) molar mass and dispersity ($Đ$ = M_w/M_n) determined via size exclusion chromatography (SEC) at 40 °C in $CHCl_3$ coupled with a Wyatt DAWN HELEOS II multi (18)-angle light scattering detector and a Wyatt Optilab TrEX dRI detector for absolute molar mass. [c] P_m is the probability of *meso* linkages between **MPL** units.

Table S5. Synthesis of Triblock Copolymer from *rac*-β-Butyrolactone and *rac*-MPL[a]

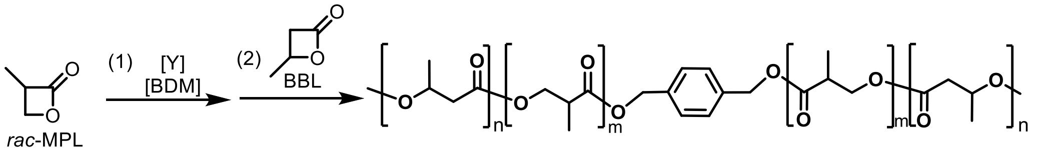

Runs	[*rac*-BBL]/[*rac*-MPL][Cat.]/[I]	[*rac*-BBL] Conv.[%]	[*rac*-MPL] Conv.[%]	Cat.	*rac*-MPL Content[b] [%]	M_n[c] [kDa]	$Đ$[c]
**1**	100:100:1:0.5	>99	>99	**Y1**	45	21.8	1.08
**2**	100:100:1:0.5	>99	>99	**Y2**	50	23.8	1.30
**3**	100:100:1:0.5	>99	>99	**Y3**	43	26.9	1.34

[a] Monomer conversion (Conv.) determined by ^{1}H NMR in $CDCl_3$, 23 °C, *rac*-BBL (0.5 mmol, 43 mg), *rac*-MPL (0.5 mmol, 43 mg) in toluene (2 M). [b] The content of *rac*-MPL was determined by ^{1}H NMR analysis of the isolated copolymer. [c] Weight-average (M_w) and number-average (M_n) molar mass and dispersity ($Đ$ = M_w/M_n) determined via size exclusion chromatography (SEC) at 40 °C in $CHCl_3$ coupled with a Wyatt DAWN HELEOS II multi (18)-angle light scattering detector and a Wyatt Optilab TrEX dRI detector for absolute molar mass.

Table S6. Synthesis of Statistical Copolymer from *rac*-β-Butyrolactone and *rac*-MPL[a]

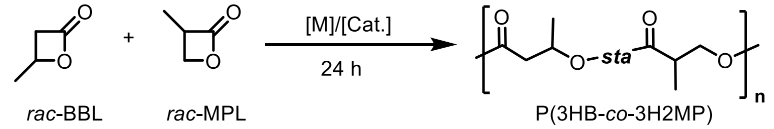

Runs	[*rac*-BBL]/[*rac*-MPL][Cat.]/[I]	[*rac*-BBL] Conv.[%]	[*rac*-MPL] Conv.[%]	Cat.	*rac*-MPL Content[b] [%]	M_n[c] [kDa]	$Đ$[c]
**1**	100:100:1:0	>99	>99	**L2+Y6**	50	56.4	1.49
**2**	140:60:1:0	>99	>99	**L2+Y6**	29	77.1	1.52
**3**	180:20:1:0	>99	>99	**L2+Y6**	11	76.7	1.84
**4**[d]	500:55:1:0	99	>99	**L2+Y6**	11	70.0	0.73
**5**[e]	2000:500:1:0	65	>99	**L2+Y6**	28	293	1.18
**6**[d]	2000:220:1:0	81	99	**L2+Y6**	13	184	1.26
**7**	100:100:1:0.5	>99	>99	**Y2**	50	30.4	1.14

[a] Monomer conversion (Conv.) determined by ^{1}H NMR in $CDCl_3$, 23 °C, [Cat.] = Catalyst. Initiator [I] = 1,4-benzenedimethanol (BDM), [*rac*-BBL] + [*rac*-MPL] = 1.0 mmol in toluene (2 M). [b] The content of *rac*-MPL was determined by ^{1}H NMR analysis of the isolated copolymer. [c] Weight-average (M_w) and number-average

(M_n) molar mass and dispersity ($Đ = M_w/M_n$) determined via size exclusion chromatography (SEC) at 40 °C in $CHCl_3$ coupled with a Wyatt DAWN HELEOS II multi (18)-angle light scattering detector and a Wyatt Optilab TrEX dRI detector for absolute molar mass. [d] [*rac*-BBL] (860 mg,10.0 mmol), [*rac*-MPL] (95.5 mg, 1.11 mmol) [e] [*rac*-BBL] (860 mg,10.0 mmol), [*rac*-MPL] (215 mg, 2.5 mmol).

Table S7. Synthesis of Statistical Copolymer of (*R*)-β-Butyrolactone and *rac*-MPL[a]

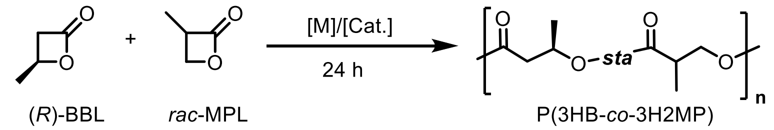

Runs	[(*R*)-BBL]/[*rac*-MPL][Cat.]	[(*R*)-BBL] Conv.[%]	[*rac*-MPL] Conv.[%]	Cat.	*rac*-MPL Content[b] [%]	M_n[c] [kDa]	*Đ*[c]
**1**	100:100:1:0	99	>99	**L1+Y6**	53	50.1	1.25
**2**	140:60:1:0	94	>99	**L1+Y6**	32	56.9	1.29
**3**	180:20:1:0	89	>99	**L1+Y6**	12	50.1	1.55
**4[d]**	500:55:1	89	>99	**L1+Y6**	12	79.1	1.54
**5[e]**	500:125:1	90	>99	**L1+Y6**	22	88.2	1.44

[a] Monomer conversion (Conv.) determined by ^{1}H NMR in $CDCl_3$, 24 h, 23 °C, [Cat.] = Catalyst. [(*R*)-BBL]+[*rac*-MPL] = 1.0 mmol in toluene (2 M). [b] The content of *rac*-MPL was determined by ^{1}H NMR analysis of the isolated copolymer. [c] Weight-average (M_w) and number-average (M_n) molar mass and dispersity ($Đ = M_w/M_n$) determined via size exclusion chromatography (SEC) at 40 °C in $CHCl_3$ coupled with a Wyatt DAWN HELEOS II multi (18)-angle light scattering detector and a Wyatt Optilab TrEX dRI detector for absolute molar mass. [d] [(*R*)-BBL] (860 mg,10.0 mmol), [*rac*-MPL] (95.5 mg, 1.11 mmol) [e] [(*R*)-BBL] (860 mg,10.0 mmol), [*rac*-MPL] (215 mg, 2.5 mmol).

Table S8. Ring-opening polymerization of *rac*-β-butyrolactone[a]

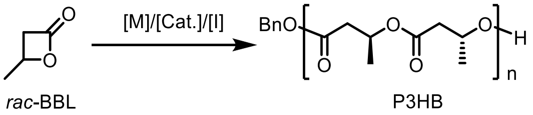

Runs	[*rac*-BBL]/[Cat.]/[I]	[Cat.]	Conv.[%]	Solvent	M_n[b] [kDa]	*Đ*[b]	P_r[c]
**1**[d]	1000:1:1	**Y2**	96	DCM (5 M)	56.0	1.06	0.84
**2**[e]	200:1:1	**Y3**	>99	DCM (2 M)	11.3	1.20	0.91

[a] Monomer conversion (Conv.) determined by ^{1}H NMR in $CDCl_3$, [Cat.] = Catalyst. Initiator (I) = BnOH, 12 h, 40 °C. [b] Weight-average (M_w) and number-average (M_n) molar mass and dispersity ($Đ = M_w/M_n$) determined via size exclusion chromatography (SEC) at 40 °C in $CHCl_3$ coupled with a Wyatt DAWN HELEOS II multi (18)-angle light scattering detector and a Wyatt Optilab TrEX dRI detector for absolute molar mass. [c] P_r is the probability of racemic enchainment between *rac*-BBL units [d] [*rac*-BBL] (860 mg, 10 mmol), 24 h. [e] [*rac*-BBL] (86.0 mg,1.0 mmol).

Table S9. Diad and triad statistical parameters for evaluating Bernoullian and Markov propagation models[17]

P_r	P_m	Diad[a]		Triad[b]			B[c]	E_1[d]	E_2[d]
		m	*r*	*mm*	*rr*	*mr*+*rm*			
0.53	0.47	0.47	0.53	0.21	0.26	0.52	0.81	0.81	1.51
0.61	0.39	0.39	0.61	0.16	0.40	0.44	1.32	0.73	1.99
0.64	0.36	0.36	0.64	0.10	0.46	0.44	0.95	0.45	4.75
0.70	0.30	0.30	0.70	0.06	0.54	0.40	0.81	0.30	9.98
0.72	0.28	0.28	0.72	0.07	0.55	0.38	1.07	0.37	7.59
0.76	0.24	0.24	0.76	0.04	0.63	0.33	0.93	0.24	16.2
0.42	0.58	0.58	0.42	0.28	0.20	0.52	0.83	0.77	1.65
0.40	0.60	0.60	0.40	0.34	0.23	0.43	1.69	1.07	0.86
0.37	0.63	0.63	0.37	0.40	0.24	0.36	2.96	1.33	0.41
0.34	0.66	0.66	0.34	0.48	0.19	0.33	3.35	1.15	0.63
0.28	0.72	0.72	0.28	0.54	0.14	0.32	2.95	0.88	1.47
0.28	0.72	0.72	0.28	0.57	0.12	0.31	2.85	0.77	2.06
0.27	0.73	0.73	0.27	0.57	0.13	0.30	3.29	0.87	1.55
0.25	0.75	0.75	0.25	0.66	0.11	0.23	5.48	0.95	1.21
0.20	0.80	0.80	0.20	0.70	0.07	0.23	3.71	0.61	4.47

[a] Measured by integrating the components of the carbonyl resonance (~173 ppm). [b] Measured by integrating the components of the methine resonance (~39 ppm). [c] Bernoulli model triad test B, where $B = 4(mm)(rr)/[(rm)+(mr)]^2$. [d] Enantiomorphic site model triad test parameters: For isoselective polymerizations, E_1 and E_2 were calculated using rr as the dominant homo-triad. where $E_1 = 2(rr)/[(rm)+(mr)]$, $E_2 = 1–4/\{[(rm)+(mr)]+2(rr)\}+1/(rr)$. For syndioselective polymerizations, E_1 and E_2 were calculated using mm as the dominant homo-triad. where $E_1 = 2(mm)/[(rm)+(mr)]$, $E_2 = 1–4/\{[(rm)+(mr)]+2(mm)\}+1/(mm)$. For syndiotactic P3H2MP with $P_r > 0.80$, the mm resonance was not observable within the ^{13}C NMR resolution, rendering the statistical parameters B, E_1, and E_2 not meaningfully determinable.

Table S10. Tensile stress-strain data of P3H2MP (M_n = 175 kDa, $Đ$ = 1.09, P_r = 0.88) dog-bone-shaped specimens

Entry	Young's modulus (MPa)	elongation at break (%)	yield stress (MPa)	ultimate tensile strength (MPa)	toughness (MJ m^{-3})
1	505	353.1	13.3	24.4	51.9
2	500	336.9	12.7	23.1	45.9
3	518	302.0	13.2	21.9	40.9
Average	507	330.7	13.1	23.1	46.2
Standard deviation	9.3	26.1	0.3	1.3	5.5

Table S11. Tensile stress-strain data of P3H2MP (M_n = 189 kDa, $Đ$ = 1.07, P_r = 0.70) dog-bone-shaped specimens

Entry	Young's modulus (MPa)	elongation at break (%)	yield stress (MPa)	ultimate tensile strength (MPa)	toughness (MJ m^{-3})
1	156	536.1	5.7	24.5	65.4
2	169	557.2	5.1	22.5	65.4
3	195	530.8	4.9	22.2	60.0
Average	174	541.4	5.2	23.1	63.6
Standard deviation	18.6	13.9	0.4	1.3	3.1

Table S12. Tensile stress-strain data of P3H2MP (M_n = 287 kDa, $Đ$ = 2.49, P_m = 0.72) dog-bone-shaped specimens

Entry	Young's modulus (MPa)	elongation at break (%)	ultimate tensile strength (MPa)	toughness (MJ m^{-3})
1	14.8	394.9	50.9	103.6
2	15.7	375.9	50.7	98.0
3	14.7	436.2	56.2	123.6
Average	15.1	402.3	52.6	108.4
Standard deviation	0.6	30.8	3.1	23.5

Table S13. Tensile stress-strain data of P3H2MP (M_n = 419 kDa, $Đ$ =1.79, P_m = 0.74) dog-bone-shaped specimens

Entry	Young's modulus (MPa)	elongation at break (%)	yield stress (MPa)	ultimate tensile strength (MPa)	toughness (MJ m^{-3})
1	18.2	395.2	9.9	60.9	123.6
2	141	409.9	11.9	63.5	139.4
3	17.2	401.3	10.4	61.4	127.0
Average	58.8	398.3	10.7	61.9	130.0
Standard deviation	71.1	4.3	1.0	1.4	8.3

Table S14. Tensile stress-strain data of P3H2MP (M_n = 973 kDa, $Đ$ = 1.42, P_m = 0.80) dog-bone-shaped specimens

Entry	Young's modulus (MPa)	elongation at break (%)	yield stress (MPa)	ultimate tensile strength (MPa)	toughness (MJ m^{-3})
1	392	353.6	16.4	63.7	129.9
2	399	356.7	15.5	63.8	129.5
3	400	365.6	15.3	65.6	133.3
Average	397	358.6	15.7	64.4	130.9
Standard deviation	4.4	6.2	0.6	1.1	2.1

References

(1) Lin, M.-H.; RajanBabu, T. V. Ligand-Assisted Rate Acceleration in Transacylation by a Yttrium−Salen Complex. Demonstration of a Conceptually New Strategy for Metal-Catalyzed Kinetic Resolution of Alcohols. *Org. Lett.* **2002**, *4*, 1607–1610.

(2) Anwander, R.; Runte, O.; Eppinger, J.; Gerstberger, G.; Herdtweck, E.; Spiegler, M. Synthesis and Structural Characterisation of Rare-Earth Bis(dimethylsilyl) Amides and their Surface Organometallic Chemistry on Mesoporous MCM-41. *J. Chem. Soc., Dalton Trans.*, **1998**, 847–858.

(3) Amgoune, A.; Thomas, C. M.; Roisnel, T.; Carpentier, J.-F. Ring-Opening Polymerization of Lactide with Group 3 Metal Complexes Supported by Dianionic Alkoxy-Amino-Bisphenolate Ligands: Combining High Activity, Productivity, and Selectivity. *Chem. Eur. J.* **2006**, *12*, 169–179.

(4) Bouyahyi, M.; Ajellal, N.; Kirillov, E.; Thomas, C. M.; Carpentier, J.-F. Exploring Electronic versus Steric Effects in Stereoselective Ring-Opening Polymerization of Lactide and β-Butyrolactone with Amino-alkoxy-bis (phenolate)–Yttrium Complexes. *Chem. Eur. J.* **2011**, *17*, 1872–1883.

(5) Tang, X.; Chen, E. Y.-X. Chemical Synthesis of Perfectly Isotactic and High Melting Bacterial Poly(3-Hydroxybutyrate) from Bio-Sourced Racemic Cyclic Diolide. *Nat. Commun.* **2018**, *9*, 2345.

(6) Li, K.; Cheng, J. L.; Wang, M.-Y.; Xiong, W.; Huang, H.-Y.; Feng, L.-W.; Cai, Z.; Zhu, J.-B. Kinetic Resolution Polymerization Enabled Chemical Synthesis of Perfectly Isotactic Polythioesters. *Angew. Chem., Int. Ed.* **2024**, *63*, e202405382

(7) Maudoux, N.; Roisnel, T.; Dorcet, V.; Carpentier, J.-F.; Sarazin, Y. Chiral (1,2)-Diphenylethylene-Salen Complexes of Triel Metals: Coordination Patterns and Mechanistic Considerations in the Isoselective ROP of Lactide. *Chem. Eur. J.* **2014**, *20*, 6131–6147.

(8) Li, Y. T.; Yu, H. Y.; Li, W. B.; Liu, Y.; Lu, X. B. Recyclable Polyhydroxyalkanoates via a Regioselective Ring-opening Polymerization of α, β-Disubstituted β-Lactone Monomers. *Macromolecules* **2021**, *54*, 4641−4648.

(9) Huang, H. Y.; Xiong, W.; Huang, Y. T.; Li, K.; Cai, Z.; Zhu, J. B. Spiro-Salen Catalysts Enable the Chemical Synthesis of Stereoregular Polyhydroxyalkanoates. *Nat. Catal.* **2023**, *6*, 720−728.

(10) Gao, Y., Westworth, X., Quinn, E. C., Nam, J., Chen, E. Y.-X.. Topological Universal Dynamic Compatibilization Enhances Recycling of Mixed Plastics. *Nat. Sustain.* **2026**, *9*, 260–270.

(11) Xu, J.; McCarthy, S. P.; Gross, R. A. Racemic α-Methyl-β-propiolactone Polymerization by Organometallic Catalyst Systems. *Macromolecules* **1996**, *29*, 4565–4571.

(12) Xu, J.; Gross, R. A.; Kaplan, D. L.; Swift, G. Chemoenzymatic Synthesis and Study of Poly (α-methyl-β-propiolactone) Stereocopolymers. *Macromolecules* **1996**, 29, 4582−4590.

(13) Bruckmoser, J.; Pongratz, S.; Stieglitz, L.; Rieger, B. Highly Isoselective Ring-Opening Polymerization of rac-β-Butyrolactone: Access to Synthetic Poly(3-hydroxybutyrate) with Polyolefin-like Material Properties. *J. Am. Chem. Soc.* **2023**, *145*, 11494−11498.

(14) Hörl, S.; Chiorescu, I.; Bruckmoser, J.; Futter, J.; Rieger, B. Influence of Ligand Design and Non-Covalent Interactions on the Isoselective Ring-Opening Polymerization of *rac*-β-Butyrolactone Using Salan and Salalen Rare-Earth Metal Catalysts. *Angew. Chem., Int. Ed.* **2025**, *64*, e202504513.

(15) Yang, F.; Zhao, J.; Tang, X.; Wu, Y.;Yu, Z.; Meng, Q. Visible Light-Induced Salan-Copper (II)-Catalyzed Enantioselective Aerobic α-Hydroxylation of β-Keto Esters. *Adv. Synth. Catal.* **2019**, *361*, 1673–1677.

(16) Wagner, N. J.; Tang, W.-C.; Wagner, J. K.; Nguyen, B. T.; Lam, J. Y.; Gibbons-Stovall, S. K.; Matias, A. C.; Martinez,S. E.; Trieu-Tran, T.; Clabaugh, G. M.; Navarro, C. A.; Abboud, I.; Flores, F. X.; Nicholas, K. M.; John, A. Molybdenum Catalysts Based on Salan Ligands for the Deoxydehydration Reaction. *Catal. Sci. Technol.*, **2024**, *14*, 3660–3673.

(17) Hocking, P. J.; Marchessault, R. H. Microstructure of Poly[(*R*,*S*)-β-hydroxybutyrate] by ^{13}C NMR. *Macromolecules* **1995**, *28*, 6401–6409.
